# The utility of generative artificial intelligence Chatbot (ChatGPT) in generating teaching and learning material for anesthesiology residents

**DOI:** 10.3389/frai.2025.1582096

**Published:** 2025-05-21

**Authors:** Zhaosheng Jin, Ramon Abola, Vincent Bargnes, Alexandra Tsivitis, Sadiq Rahman, Jonathon Schwartz, Sergio D. Bergese, Joy E. Schabel

**Affiliations:** Department of Anesthesiology, Stony Brook University Hospital, Stony Brook, New York, NY, United States

**Keywords:** artificial intelligence, graduate medical education, large language model, anesthesiology residency, generative AI

## Abstract

The popularization of large language chatbots such as ChatGPT has led to growing utility in various biomedical fields. It has been shown that chatbots can provide reasonably accurate responses to medical exam style questions. On the other hand, chatbots have known limitations which may hinder their utility in medical education. We conducted a pragmatically designed study to evaluate the accuracy and completeness of ChatGPT generated responses to various styles of prompts, based on entry-level anesthesiology topics. Ninety-five unique prompts were constructed using topics from the Anesthesia Knowledge Test 1 (AKT-1), a standardized exam undertaken by US anesthesiology residents after 1 month of specialty training. A combination of focused and open-ended prompts was used to evaluate its ability to present and organize information. We also included prompts for journal references, lecture outlines, as well as biased (medically inaccurate) prompts. The responses were independently scored using a 3-point Likert scale, by two board-certified anesthesiologists with extensive experience in medical education. Fifty-two (55%) responses were rated as completely accurate by both evaluators. For longer responses prompts, most of the responses were also deemed complete. Notably, the chatbot frequently generated inaccurate responses when asked for specific literature references and when the input prompt contained deliberate errors (biased prompts). Another recurring observation was the conflation of adjacent concepts (e.g., a specific characteristic was attributed to the wrong drug under the same pharmacological class). Some of the inaccuracies could potentially result in significant harm if applied to clinical situations. While chatbots such as ChatGPT can generate medically accurate responses in most cases, its reliability is not yet suited for medical and clinical education. Content generated by ChatGPT and other chatbots will require validation prior to use.

## Introduction

ChatGPT is a large language model (LLM)-based chatbot that has garnered much attention over the last few years. Notably, it responds to prompts on biomedical topics, and can structure the response in a manner appropriate for an academic discussion (Chang, 2020). The potential utility of this in research, education and clinical practice has become a topic of considerable interest.

According to the latest Accreditation Council for Graduate Medical Education (ACGME) guidelines, the curriculum for anesthesiology residency “must contain instruction that encompasses clinical anesthesiology and basic science topics” (ACGME, 2022) There is however limited guidelines on how the required knowledge should be attained (Review Committee for Anesthesiology, 2023). Online resources have become an integral part of graduate medical education. Peer-reviewed publications provide reliable information on specific topics but are not always optimized for learning. Digital textbooks can be comprehensive but may not be up to date. Other modalities such as Twitter, MedEdPortal, blogs, podcasts can cater to different learning styles but vary in reliability and quality. Both learners and teachers must often delve into multiple sources for the optimal combination. The increasing utilization of digital learning resources has uncovered a growing need for information curation, filtering and organization in a personalized manner to optimize learning.

LLMs like ChatGPT and Gemini employ a conversational chatbot interface for interacting with users. LLMs generate responses to user prompts by selecting a sequence of words with the highest probability of appearing correct to the user, using a generative pre-trained transformer (GPT) (Bahl et al., 1983). To acquire such capability, LLMs are trained upon a large and diverse corpus of text (Radford et al., 2025). Reports on an earlier iteration of the GPT model listed the Common Crawl (a free-to-access archive of internet content) as the highest weighed training dataset, followed by additional web text and books (Brown et al., 2020). Additionally, each iteration of ChatGPT is also limited by the information available at the time of its training, known as knowledge cutoff. The knowledge cutoff is reported to be 2021 for ChatGPT 3.5, and 2023 for ChatGPT 4.0 (OpenAI, 2025).

In contrast to fixed content such as a textbook, advantages of using LLMs for graduate medical education include the capacity to integrate high volume of information from different sources, conversational responses that could be rephrased to enhance comprehension and flexible information organization to meet the specific need of the learner.

Despite the impressive features of chatbot LLMs, concerns over the bias and inaccuracy of its responses have been highlighted in both medical and non-medical contexts. The use of ChatGPT and other chatbots in medicine and biomedical fields has been a topic of ongoing debate. Several previous studies have reported that ChatGPT is able to generate reasonably accurate responses to specific medical questions (Gilson et al., 2023). Most recently, Gupta et al. reported that ChatGPT had an accuracy of 66.5% when promoted with Anesthesiology Continuing Education (ACE) questions (Gupta et al., 2024). In this exploratory study, we evaluated the validity of ChatGPT generated content as educational material for resident physicians during their first introduction to anesthesiology using a range of prompt formats.

## Methods

We used ChatGPT 3.5 and ChatGPT 4.0 (Open AI, San Francisco) to generate responses to anesthesiology-related prompts. This is a free-to-use model of the platform, which at the time of the testing, had a core knowledge base last updated in 2021 and 2023, respectively (OpenAI, 2025).

### Prompt design

Prompt topics were selected based on the Anesthesia Knowledge Test 1 (AKT-1), a standardized exam designed by Inter-Hospital Study Group for Anesthesia Education (2024) and used to assess anesthesiology residents after the first 4–6 week of their clinical anesthesiology training. This training milestone is selected as residents often receive extensive didactics during this period, thus potentially benefiting from AI augmented teaching material. Since the original exam questions are protected material, prompts were generated based on the “key words” released by the exam providers (Supplementary Appendix 1). Permission was obtained from IHSGAE for the use of the key words.

Several classes of prompts were designed to evaluate different aspects of the chatbot functionality (Table 1). Focused factual prompts inquire on specific knowledge point (s) and were designed to test the factual integrity of the generated content. Extended response prompts inquire on broader topics and were designed to evaluate the organization of information in addition to accuracy. A subset of these prompted for a structured lecture outline on a particular topic. These accounted for the majority of the questions.

**Table 1:** Prompt category breakdown.

Prompt type*	Number	Description	Examples (not used)
Focused prompts (S)	40	Questions amenable to concise answers with specific knowledge point (s)	What is the benefit of adding Sodium Bicarbonate to epidural top off solution?
Biased prompts (B)	10	Factually incorrect prompt	What is the advantage of using an LMA for a term parturient undergoing emergency cesarean delivery with general anesthesia?
Prompts for references (E)	10	Request for journal article references to a specific topic	Can you list some published clinical trials which support the use of tranexamic acid for postpartum hemostasis?
Extended response prompts (L)	30	Requires longer responses and multiple relevant knowledge points	A patient with a known placenta increta is scheduled for cesarean delivery, what are the anesthetic considerations?
Lecture outline prompts (M)	5	Request for a presentation design addressing a knowledge point	Can you make a 5 min presentation on the pharmacokinetics of drugs in pregnancy
Rescore	10	Resubmission of questions which generated the lowest scored responses	
Total	105		

*Individual prompts were identified according to the type of the prompt, letter abbreviation in brackets; followed by an identification number.

We also designed specific prompts to address some of the known issues of chatbots. One such issue is the generation of factually inaccurate responses to when the prompt itself contains inaccurate information (e.g., “how do house mice fly”). We therefore purposefully included prejudiced and factually incorrect prompts (from here on referred to as “biased prompts”) and evaluated the accuracy of the response. Another known issue with language models is artificial hallucination, in which a machine generates an output that does not correspond to any real-world input (Alkaissi and McFarlane, 2023). In the context of biomedical writing, this can manifest as inaccurate reference citations. To address this, we created prompts which requests for references and then scrutinized the accuracy of the references in the response.

The Chatbot prompts were written by four of the authors (AT, SR, VB, ZJ), using the key words listed in (Supplementary Appendix 1). The team members were instructed to include as many available key words as feasible, while minimizing using the same key word repeatedly for different prompts. The prompts were then reviewed by ZJ and JS for internal consistency (including the prompt format, presence of ambiguity and the minimum information required for response), those which did not pass the review process were rewritten by ZJ. A new chat session was used for each prompt, including instances where the prompts needed to be rephrased for clarity; this reduced the risk of bias and prompt injection due to previous conversations.

### Response grading

Each recorded response was then independently reviewed by two authors not involved in the prompt creation (JES, RA). The raters are board-certified anesthesiologists each with more than 10 years’ experience in anesthesiology resident education. The raters were asked to provide a single rating for focused factual prompts, since these only required one or a few essential knowledge points. A similar system was used for the biased prompts and prompts for references. Given the larger amount of essential information in response to open-ended prompts, raters were asked to rate the accuracy and completeness as separate domains. We employed a 3-point Likert scale, where 3 points represent a response comparable to textbooks or expert teachings; 2 points represent minor inaccuracies or omissions; 1 point represents major inaccuracies or omissions, thus not suitable for educational use. The evaluators were asked to follow the scoring guideline (Supplementary Appendix 2) and were given a set of test responses to familiarize themselves with the scoring criteria.

The scores from both raters were then added to generate the final score for the response (out of 6). Responses that scored 6 points were considered suitable for educational use, responses that scored 3 points or lower were considered not suitable. Those scored 4 or 5 points are considered situationally useful but highlight the need for fact checking and expert review. The scores across a domain or a group were reported as median with interquartile range (IQR).

Weighted Cohen’s kappa was used to evaluate the inter-rater reliability. Ratings for the focused factual prompts, biased prompts and prompts for references were pooled for the inter-rater reliability. The accuracy and completeness scores of the longer response prompts were analyzed separately.

Goodman et al. (2023) noted in their study that chatbot performance in answering biomedical questions improved significantly over a short period of time. We therefore identified 10 of the lowest scoring responses, submitted the same prompt again and regraded the new responses. This corresponded with the platform upgrade from version 3.5 to version 4.

## Results

A total of 95 prompts were created, covering 89 key words based on the 2022 exam. The list of finalized prompts can be found in Supplementary Appendix 1. The initial prompts set was submitted to ChatGPT 3.5.

**Figure 1:**
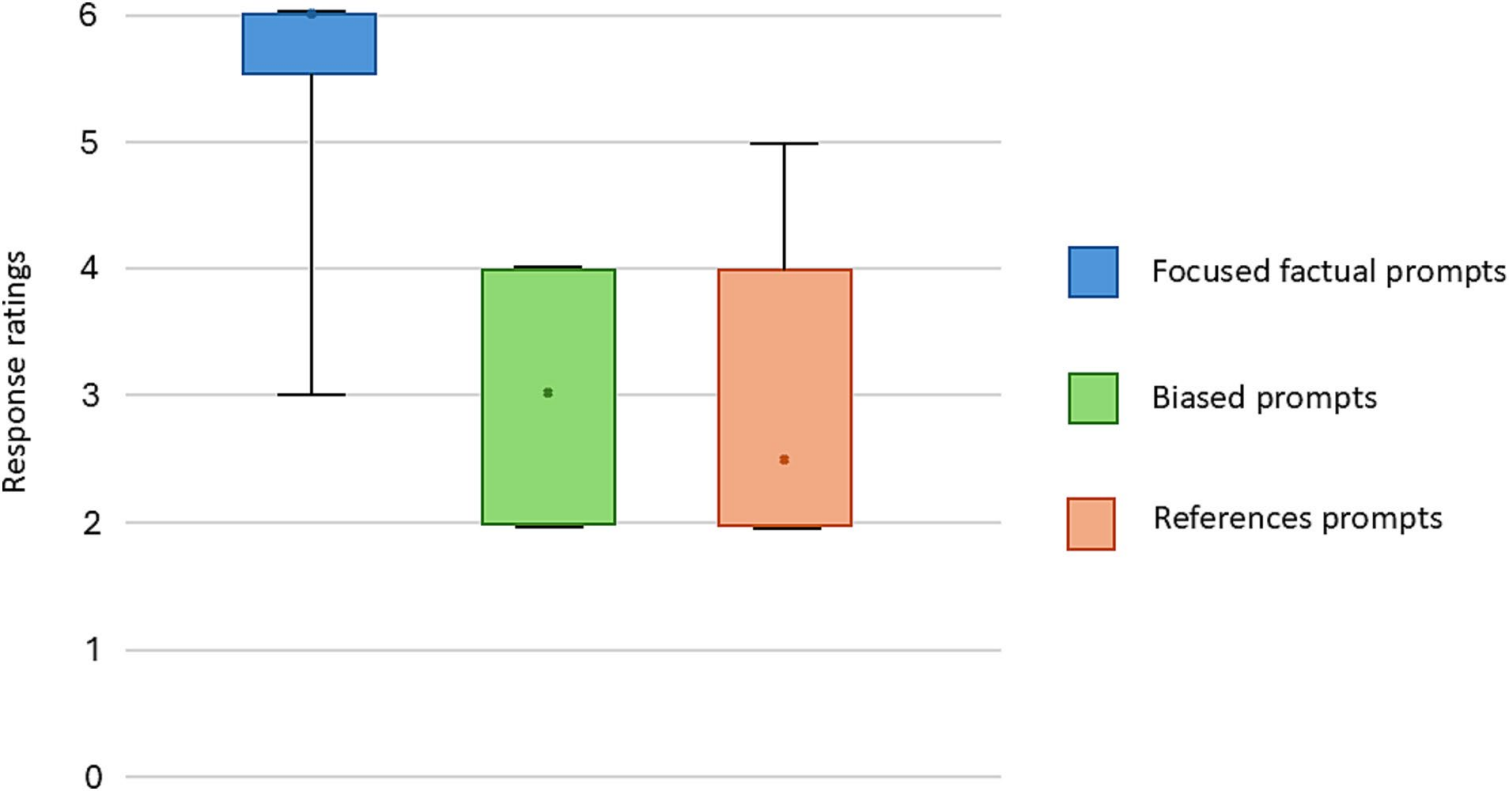
Score summary of focused factual prompts, biased prompts and prompts for references.

**Figure 2:**
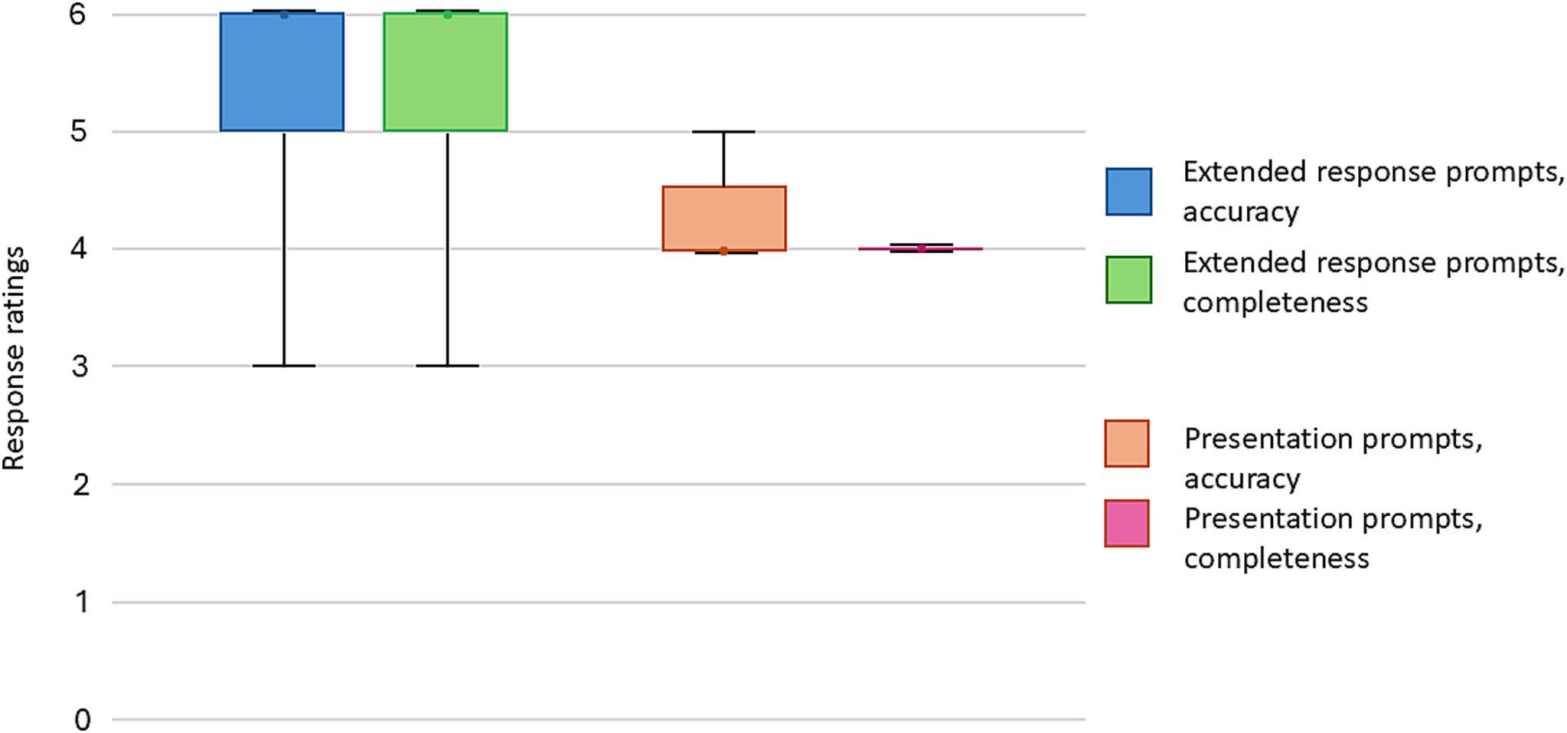
Accuracy and completeness score summary of the extended response prompts and lecture outline generation.

Forty focused factual prompts were submitted to the chatbot (see Supplementary Appendix 1), the mean response length was 143 words [standard deviation (SD): 72]. When evaluated by board certified anesthesiologists, 30 out of the 40 responses were rated as being comparable to textbook or expert lecture by both raters. The median expert evaluated score was 6 (out of 6, IQR: 5.5–6) (Figures 1, 2; Table 2). Three (7.5%) responses were noted to contain significant inaccuracies. One response stated that a patient with a recent stroke, but no other medical comorbidities is classified as ASA 2 (S23). One response listed succinylcholine among drugs most likely to cause perioperative anaphylaxis (S38), presumably due to the word association with neuromuscular blocking agents. Notably, some responses included far more information than what was requested in the prompt (Table 3).

**Table 2:** Summary of results.

Prompts type (number of prompts)	Response word count (SD)	Median score (IQR)	Score = 6 (%)	Score ≤ 3 (%)	Inter-rater reliability*
Focused factual prompts (40)	143 (73)	6 (5.5–6)	30 (75%)	3 (7.5%)	*κ* = 0.45, *p* < 0.01
Biased prompts (10)	279 (117)	3 (2–4)	0	6 (60%)	
Prompts for references (10)	233 (110)	2.5 (2–4)	0	7 (70%)	
Extended response prompts (30) Accuracy Completeness	347 (82)	6 (5–6) 6 (5–6)	22 (73%) 22 (73%)	0 1 (3.3%)	Accuracy: κ = −0.14, *p* = 0.12 Completeness: *κ* = 0.16, *p* = 0.03
Lecture outline prompts (5) Accuracy Completeness	513 (70)	4 (4–4.5) 4 (4–4)	0 0	0 0	

*Cohen’s weighted kappa.

**Table 3:** Notable observations by the raters.

Theme	Example prompt ID	Explanation
Information that can cause harm if applied clinically	S13, L6, B1, B2, B5, B7, B9	Contents of certain responses, especially those in response to a “biased” prompts, can cause significant harm if applied in a clinical situation
Clinical decision making and practice	L6	“Using the higher end of the oxygen consumption rate for safety” demonstrates sound clinical decision making in an area of ambiguity
	L5, L21	Responses do not place appropriate emphasis on important action points (e.g., calling for help and securing the airway during an emergency)
Conflation of similar concepts and text strings	S11, S38, B2	Specific pharmacological properties of neuromuscular blocking drugs (anaphylaxis risks and metabolism) were incorrectly attributed to a different neuromuscular blocking drugs
	S23	Referred to the ASA Physical Status Classification, but identified the wrong classification
	S8, M2	Blood: gas coefficient descriptors were applied to the wrong inhaled anesthetic
Inaccuracy due to conflicting information	S38	Given the conflicting reports of whether antibiotics or neuromuscular blocking drugs are the most common cause of perioperative anaphylaxis, the response does not reflect the most specific and up-to-date literature (NAP6)
Incorrect application of mathematical equations	S36	Incorrect application of the ideal gas law without accounting for the change in the molar quantity of Oxygen in the cylinder
	L6	When asked to estimate the safe apneic time, incorrectly calculates the time required to consume total body Oxygen reserve, which is not compatible with life
Conflicting information within the response	B4	States that sodium content in normal saline is below the physiologic range, while also providing the numerical data indicating that saline has a higher sodium content than plasma
Inclusion of information not specific to the prompts	S24, S26, S28, B6	Asked to provide a specific piece of information, the response expanded into discussions which went beyond what was required.
Responses include guidelines which are not region specific	L10	When asked to provide algorithm for the management of difficult airway, guideline from United Kingdom was cited (authors are based in the US)
Response noted that ChatGPT does not have real-time access to internet for information	E6	“I cannot browse the internet or access real-time databases”

When a biased prompt was used (10 prompts, see Supplementary Appendix 1), the quality of the responses was notably lower. None of the responses were assigned full points, with a median score of 3 (IQR: 2–4) (Figure 1). Six (60%) of the responses received a score of 3 and below. Examples of inaccuracies included discussion about placement of thoracic spinal anesthesia, bag-mask-ventilation of a patient requiring rapid sequence induction (RSI), and the use of sevoflurane in malignant hyperthermia. For all the examples above, expert raters noted that the information could result in significant harm if applied to clinical practice.

Ten prompts were submitted which requested specific references to biomedical literature (see Supplementary Appendix 1), none of the responses received the full points. The median score was 2.5 (IQR: 2–4) (Figure 1). This reflected the inaccuracies in references to biomedical publications, including nonexistent references (E1, E2, E7), hallucinated additional authors (E3), hallucinated author and journal (E8). There were multiple responses where the Digital Object Identifier (DOI) was inaccurate for an existing reference.

A total of 30 extended response prompts were submitted (see Supplementary Appendix 1), the mean response length was 347 words (SD: 82). The raters were asked to provide separate ratings for accuracy and completeness. In terms of accuracy, 22 (73%) responses were rated as being comparable to textbook or expert teaching. The median score was 6 (IQR: 5–6) (Figure 2). No response received a score of 3 or lower. In terms of completeness, 22 (73%) responses were rated as being comparable to textbook or expert teaching. The median score was 6 (IQR: 5–6). One response received a score of 3, which suggests significant omissions. Raters noted inconsistent demonstration of sound clinical decision making (Table 3). Additionally, when asked to provide information on difficult airway guidelines, ChatGPT provided the guidelines from a different country to that of the authors.

Five lecture prompts were submitted to the chatbot, the mean response length was 513 words (SD: 70). Most prompts received scores of 4, for both accuracy and completeness, signifying some degrees of inaccuracies and omissions (Figure 2).

**Figure 3:**
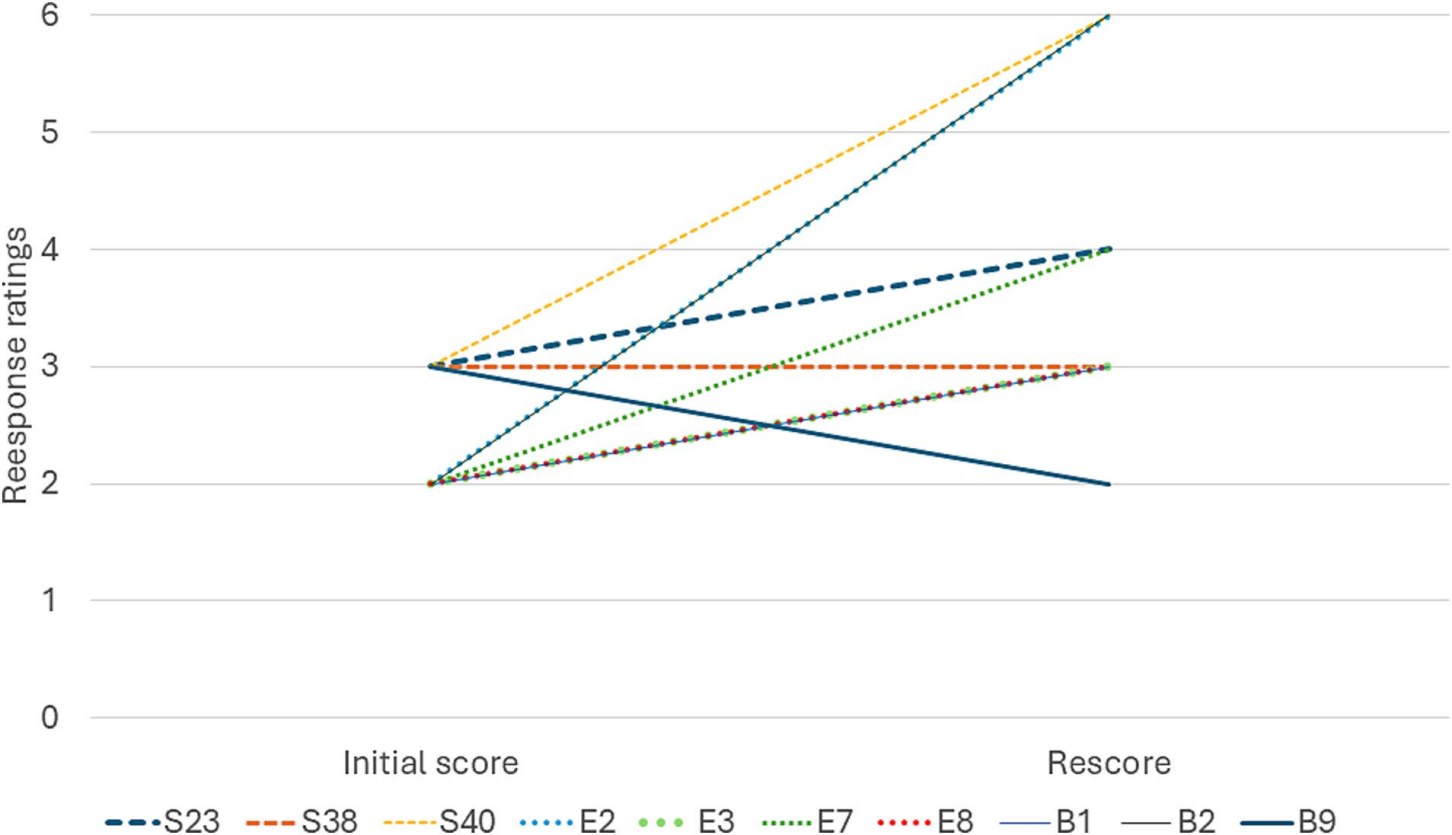
Change in ratings from the initial response (ChatGPT 3.5) to the second response (ChatGPT 4.0), each line represents one individual prompt.

In accordance with the analysis plan, 10 of the lowest scoring prompts were rescored. These consisted of focused factual prompts, biased prompts and prompts for references (see Supplementary Appendix 3). Four out of the 10 responses improved by 2 or more points from the original, this included 1 (33%) focused factual prompt, 2 (50%) prompts for references and 1 (33%) biased prompt (Figure 3).

For the single-rating responses (focused factual prompts, biased prompts and reference prompts), Cohen’s weighted kappa was 0.45 (*p* < 0.01), indicating good interrater reliability (Regier et al., 2013). For longer response prompts, the weighted kappa for accuracy scores was −0.14 (*p* = 0.12), indicating no agreement beyond random chance; the weighted kappa for the completeness scores was 0.16 (*p* = 0.03), indicating poor agreement.

Additionally, several themes emerged during the debriefing with the raters. The responses were generally longer than was necessary to convey the required information. The extra information does not directly address the questions posed by the prompts, but the inaccuracy in such content reduces the overall quality of the response. Raters noted uncertainties addressing such “peripheral” inaccuracies during the scoring process.

Additionally, the following were the subjective observations of our raters. A common cause of inaccuracy seems to be the conflation of adjacent concepts and similarly worded text strings. The answers were well categorized, but at times were organized in a sequence which suggested a lack of clinical insight. Some responses demonstrated a degree of understanding and insight into the nuances of the topic. There were instances where the response made reference to an appropriate source of information but had the wrong clinical information. A full list of themes identified is listed in Table 3.

## Discussion

In this exploratory study, we evaluated the quality of responses to anesthesiology related prompts by ChatGPT, a popular LLM chatbot that is widely available to learners and educators alike. We found that ChatGPT is reasonable accurate in generating a response to most entry level anesthesia related prompts. ChatGPT can provide larger volume of anesthesia related content in an organized manner. However, we also found that ChatGPT can sometimes provide inaccurate information, which has the potential of causing significant harm (e.g., administering volatile anesthesia in patients with a history of malignant hyperthermia). This issue is seen particularly with “biased” prompts and prompts for specific medical references. The shortcoming of chatbots in these specific areas are concerning and brings into question the utility of chatbots for “unsupervised” medical education, since there is an appreciable risk that learners could be given inaccurate information, which could negatively impact clinical performance.

The utility of Chatbot platforms in medicine and graduate medical education has been highlighted in several recent studies. In a recent research letter, Gupta et al. (2024) submitted Anesthesiology Continuing Education questions to ChatGPT, and reported lower response accuracy with more advanced questions, this may represent the scarcity of advanced anesthesia topic texts built into the core knowledge matrix. Interestingly the author did not find a difference between general and specialty-based questions. Our study reported overall accuracy that is somewhat comparable with Gupta’s team. However, we note that even for entry level knowledge, biased prompts often resulted in medically inaccurate responses.

Ayers et al. compared ChatGPT vs. physician generated responses to patient questions from online forums. Blinded evaluators preferred the ChatGPT response in 78% of the evaluations. ChatGPT generated responses were also thought to be more empathetic and of better quality (Ayers et al., 2023). Hirosawa et al. evaluated ChatGPT’s ability to generate differential diagnosis lists when prompted with complex clinical vignettes. They reported that ChatGPT 4 performed better than ChatGPT 3.5, was accurate in 60–83% of the cases, and was comparable to physician differential diagnoses (Hirosawa et al., 2023). Additionally, Kung et al. reported that ChatGPT was able to complete the United States Medical Licensing Exam (USMLE) with reasonable accuracy (Kung et al., 2023).

The popularization of LLM such as ChatGPT has significantly shifted the paradigm in biomedical education. Critically appraising the relevant original research for every clinical topic can be prohibitively time consuming, the convenience of using LLM to organize large amounts of information cannot be underestimated. On the other hand, the reliability of LLMs in biomedical education is actively debated. The factors which may influence the role of LLM chatbots include the sourcing of its information, the generative algorithm, and user-related factors (such as prompt design).

One of the major limitations of commercial LLMs is the reliability of their source of information. In addition to its knowledge cutoff, LLMs are trained using text corpus that may not have undergo the scientific rigor of peer reviewed biomedical journals, any inaccuracies and biases from the sources can negatively influence all the subsequent output. A recent systematic review by Daraz et al. (2019) reported that the quality of online medical information varies considerably by specialty, with anesthesiology and internal medicine having higher overall quality. Evans et al. (2022) found that open access anesthesiology education websites often did not list the references, nor a transparent editorial review process. Thus, there are ongoing discussions regarding the advantages of a LLM using only selected biomedical resources as its core knowledge base (Singhal et al., 2023).

LLM chatbots provide a natural language-like response to prompts after training upon a corpus of existing text. Expertise on a topic (e.g., provision of anesthesia) is not explicitly programmed into the algorithm. Therefore, critical appraisal of the source material is not its primary function. When a string of text is entered into the LLM, the text is parsed into words via tokenization. Words and their positions are endcoded (Kotei and Thirunavukarasu, 2023). The encoded input passes through the transformer architecture of the deep neural network. Attention sublayers within the neural network quantify relationships between input words, a behavior known as self-attention that resembles understanding word context (Vaswani et al., 2017). Simultaneously, other attention sublayers quantify relationships between the prompt words and output text, to help produce a chatbot response. The “generative” output from the LLM emerges by converting the processed “interpretation” of the prompt into a probability distribution across all words in the model’s output vocabulary. The word sequence with the highest assigned probability is selected as generated output. The scientific limitation of such mechanism is perhaps best highlighted when it comes to the generation of references and identifiers, where the generative process may not prioritize the accurate recreation of the text string that describes a particular reference or DOI.

It is also not clear to what extent ChatGPT and other LLMs are “trained” to provide scientifically robust biomedical responses. To train an LLM before deployment, hundreds of billions of parameters must be calculated by feeding large corpora of text. This consists of a “self-supervised” learning (Kotei and Thirunavukarasu, 2023) where the algorithm utilizes part of the input data and evaluates itself against another portion of the input data; and “supervised” learning, where human-labeled outputs are utilized as part of a “fine-tuning” to ensure desired LLM functionality within a particular context. ChatGPT was reported to have undergone supervised learning and human feedback. It is not clear if the human evaluators who participated in the supervised learning process have sufficient biomedical training to provide feedback specific to output in those fields.

Prompt engineering is an emerging field that focuses on the construction of prompt structures for optimal user results, including in the biomedical field (Wang et al., 2024). Considerations to prompt design include the information provided (such as the context and goal of the interaction), nature of the request, iterative refinement (Meskó, 2023). Additionally, LLM chatbots demonstrate differing performance depending on the architecture of the requested tasks (Wang et al., 2024). One of the key risks associated with unrestricted use of LLM in graduate medical education stems from insufficient understanding of its utility and limitations, such as what information it has access to, and the level of comprehension it is capable of Dave et al. (2023). For graduate medical education, it is prudent to advise learners to avoid using prompts which may favor a certain response (i.e., “neutral” prompts).

As with the skills to critical appraisal of scientific literature, there is now an urgent need to train physicians in understanding the basics of LLM and how to safely use it in medical context. Emphasis should be placed on understanding the functional limitations of LLM and common pitfalls associated with its use. Additionally, a cursory understanding of the underlying technical mechanism may enhance AI literacy. Opportunities for improving AI and LLM literacy among physicians and medical students include medical school (Laupichler et al., 2024), as well as postgraduate educational events (Schubert et al., 2025). While it is likely that there will be situations in which constructive use of chatbots is warranted, this remains a largely unexplored topic.

### Limitations

To the best of our knowledge, this is the first study which has attempted to systematically evaluate the utility of ChatGPT in the field of anesthesiology. Our study nevertheless has several limitations. Firstly, the primary interaction was done using the ChatGPT 3.5 platform, which at the time of the study completion has been replaced by ChatGPT 4.0. This reflected the amount and the length of responses which needed to be reviewed by the expert raters. While version 4.0 had a more recent knowledge cutoff, it is unlikely that the available online information regarding entry-level anesthesiology topics changed significantly from 2021 to 2023. There were some improvements between the two versions of the chatbot, hallucinated references and incorrect response to biased prompts both persisted in the responses generated by version 4.0. Given the rapid pace of LLM development, further testing using the up-to-date platform is warranted. We also note the relatively small number of prompts when compared to other similar published studies. Since the current study focuses on only one specialty and is limited to “entry level” knowledge expected of novice trainees, there was a limited selection of topics for generating prompts.

The scoring system used to evaluate the responses is pragmatically designed based on other similar studies, a 3-point Likert score was employed to optimize scoring reproducibility in the case of minor inaccuracy or omission. For longer response prompts, a score scale of 1–3 may not reflect the granularity within the quality of the responses. It should be noted that ChatGPT often elaborated beyond the scope of the prompt, with varying degrees of relevance and accuracy. Raters reported that since the additional, superfluous information did not directly address the prompt questions, there was uncertainty over how it should influence the score. This introduced a source of score variability and was reflected in the low interrater reliability observed in the longer responses. Further, the repeated measurements by two raters, compounded by the presence of interrater variability precluded statistical testing. Thus, it is only possible to draw qualitative conclusions on ChatGPT’s performance in the various areas.

In our current study, we did not attempt to reengineer prompts in cases of inaccurate response. This is because of the time lag between response generation, review and analysis, which introduces real-time unsupervised model learning as a confounder we cannot meaningfully address. While prompt engineering will likely become an increasingly relevant skill in the biomedical field, expertise is not widely available at this point. Thus, a simple input–output prompt structure likely is likely reflective of those submitted by learners and educators.

It is also largely unknown if our finding regarding the use of chatbots reflects its utility in clinical decision making. Our study and others have found isolated examples where ChatGPT responses emulated what would be considered reasonable clinical decision making. It is unclear to what extent LLM chatbots are able to do this consistently in the full extent of clinical scenario complexity. Works by Kung and Reidel evaluated the instance of insights in ChatGPT responses, an instance of text that infers knowledge and deduction outside of what was offered in the question (Kung et al., 2023; Riedel et al., 2023). Our evaluation also identified instances where ChatGPT demonstrated sound clinical reasoning (such as maintaining a safety margin). Given the complexity of clinical medicine, far more extensive testing is needed to evaluate if the generative process of chatbots can appropriately emulate a sound decision making process.

## Conclusion

ChatGPT generated responses to focused questions were factually accurate in the majority of the cases, responses to open ended questions were similarly accurate and complete. Notably, the responses tend to provide more information than is necessary to answer the question. There were considerable inaccuracies in a minority of the responses, some of which could result in harm to the patient if implemented in practice. In addition, it had limitations in specific areas such as generating references and answering biased prompts. Further prompt engineering may remedy some of these shortcomings. End user (anesthesiology learners and educators) training in LLM limitations may also optimize the quality of the generated content. In the current state, responses generated by chatbots will require rigorous cross-checking by learners and educators, in addition to adherence with institutional policy. Future solutions may include the development of core knowledge matrix and model training specific to anesthesiology or the wider biomedical sciences.

## Data Availability

The original contributions presented in the study are included in the article/Supplementary material, further inquiries can be directed to the corresponding author.
