# Supplementary material for: The utility of generative artificial intelligence Chatbot (ChatGPT) in generating teaching and learning material for anesthesiology residents
Source: Front Artif Intell. 2025 May 21;8:1582096. doi: 10.3389/frai.2025.1582096 (PMC12133725; doi:10.3389/frai.2025.1582096)
Supplement: Supplementary file 1 [file Data_Sheet_1.pdf]

# *The Utility of Generative Artificial Intelligence Chatbot (ChatGPT) in Generating Teaching and Learning Material for Anesthesiology Residents- supplementary material*

## Appendix 1: prompts and responses

Focused factual prompts (40 prompts):

| Q. number & Keyword | Prompts and responses | Accuracy rating |
|---|---|---|
| 1. Correct sizing of LMA | **What is the correct sizing of a laryngeal mask airway?**<br>**Response**: The correct sizing of a laryngeal mask airway (LMA) typically depends on the patient's weight or other anatomical considerations. LMAs come in various sizes, usually ranging from size 1 (for infants) to size 5 (for adults).<br>Here's a general guideline based on weight:<br>- Size 1: Up to 5 kg (11 lbs)<br>- Size 1.5: 5-10 kg (11-22 lbs)<br>- Size 2: 10-20 kg (22-44 lbs)<br>- Size 2.5: 20-30 kg (44-66 lbs)<br>- Size 3: 30-50 kg (66-110 lbs)<br>- Size 4: 50-70 kg (110-154 lbs)<br>- Size 5: Over 70 kg (Over 154 lbs)<br>However, it's important to note that these sizes can vary between manufacturers, and selecting the appropriate size also depends on individual patient factors, such as neck anatomy and medical condition. Always refer to the manufacturer's guidelines and consider the patient's specific needs and characteristics when choosing the correct size of an LMA. | 6 |
| 2. Hypercapnia during laparoscopy | **Why does hypercapnia occur during laryngoscopy?**<br>**Response**: Hypercapnia during laryngoscopy can occur due to several reasons:<br>1. **Hypoventilation**: During laryngoscopy, especially if it's prolonged or if there are complications, a person may not breathe as deeply or frequently as they normally would. This can lead to an accumulation of carbon dioxide (CO2) in the bloodstream, causing hypercapnia.<br>2. **Airway Obstruction**: Manipulation of the airway during laryngoscopy can sometimes lead to partial or complete obstruction of the air passage, reducing the exchange of gases in the lungs. This obstruction can trap CO2 in the body, resulting in hypercapnia. | 6 |

| | | |
|---|---|---|
| | 3. **Ventilation-Perfusion Mismatch**: Laryngoscopy can affect the matching of ventilation (airflow) and perfusion (blood flow) in the lungs. Disruption of this balance can impair the removal of CO2 from the bloodstream, contributing to hypercapnia.<br>4. **Respiratory Depression**: Some individuals may experience respiratory depression during laryngoscopy, particularly if sedatives or anesthetics are administered. Reduced respiratory effort can result in inadequate removal of CO2 from the body, leading to hypercapnia.<br>Overall, hypercapnia during laryngoscopy is a concern because it indicates inadequate ventilation and can lead to respiratory compromise if not addressed promptly. It's essential for healthcare providers to monitor respiratory parameters closely during the procedure and intervene if necessary to maintain adequate gas exchange. | |
| 3. Color coding of gas cylinders | **What are the United States and international color codings of gas cylinders?**<br>**Response**: Gas cylinders typically use color codes to indicate the type of gas they contain, as well as other important information such as the level of hazard. However, it's important to note that color codes can vary by region and standardization organization. Here are some common color codes used in the United States and internationally:<br>### United States:<br>1. **Compressed Gas Association (CGA) Color Code**:<br>- CGA has established color codes for gas cylinders in the United States.<br>- For example:<br>- Oxygen: Green<br>- Acetylene: Maroon<br>- Nitrogen: Black<br>- Carbon Dioxide: Gray<br>- Hydrogen: Red<br>### International:<br>1. **ISO Color Code**:<br>- The International Organization for Standardization (ISO) has its own color codes for gas cylinders.<br>- These color codes may differ slightly from those used in the United States.<br>- For example:<br>- Oxygen: White<br>- Acetylene: Maroon<br>- Nitrogen: Black<br>- Carbon Dioxide: Gray<br>- Hydrogen: Red<br>### Safety Precautions: | 6 |

| | | |
|---|---|---|
| | - It's important to verify the gas contained in a cylinder by reading the label or tag, as relying solely on color codes can be risky.<br>- Always handle gas cylinders with caution and follow appropriate safety protocols.<br>These are general guidelines, and specific industries or regions may have additional color codes or variations. Always refer to local regulations and standards for accurate information. | |
| 4. Laminar flow in a tube | **What constitutes laminar flow in a tube?**<br>**Response**: Laminar flow in a tube occurs when a fluid flows smoothly and orderly, with parallel layers of fluid moving at different velocities. Several factors contribute to laminar flow:<br>1. **Low Reynolds number**: Reynolds number (Re) is a dimensionless quantity that characterizes the flow regime of a fluid. In the case of flow through a tube, laminar flow typically occurs at low Reynolds numbers, typically below 2,000.<br>2. **Low velocity**: Laminar flow tends to occur at relatively low velocities where the inertial forces are much smaller compared to the viscous forces. This results in smooth, ordered flow without significant mixing or turbulence.<br>3. **High fluid viscosity**: Viscosity refers to a fluid's resistance to deformation. In laminar flow, the fluid viscosity plays a significant role in maintaining the orderly movement of fluid layers. Higher viscosity fluids are more likely to exhibit laminar flow.<br>4. **Smooth tube walls**: Irregularities or roughness on the walls of the tube can disrupt laminar flow by causing turbulence. Therefore, laminar flow is more likely to occur in tubes with smooth walls.<br>In summary, laminar flow in a tube is characterized by smooth, orderly flow with layers of fluid moving parallel to each other, typically at low velocities and low Reynolds numbers, and in fluids with relatively high viscosity. | 6 |
| 5. Pin index system | **What is the pin index safety system?**<br>**Response**: The Pin Index Safety System (PISS) is a safety mechanism used in medical gas cylinders to prevent the accidental connection of the wrong gas to medical gas supply systems.<br>In this system, each medical gas cylinder valve is equipped with two pins, and each gas has a unique configuration of pin positions. Additionally, the yoke or regulator used to connect the cylinder to the medical gas supply system has corresponding holes that align with the pins on the cylinder valve. This ensures that only the correct gas can be connected to the system.<br>The Pin Index Safety System helps to prevent dangerous situations that could arise from the accidental connection of the wrong gas, which could have serious health implications for patients. | 6 |
| 6. Adverse effects ondansetron | **What are the adverse effects of ondansetron?** | 4 |

| | | |
|---|---|---|
| | **Response**: Ondansetron, commonly used to prevent nausea and vomiting caused by chemotherapy, radiation therapy, or surgery, can have some adverse effects. These may include:<br>1. Headache<br>2. Fatigue<br>3. Constipation<br>4. Diarrhea<br>5. Dizziness<br>6. Blurred vision<br>7. Dry mouth<br>8. Flushing<br>9. Fever<br>10. Rash or itching<br>11. Changes in heart rhythm (rare)<br>12. Serotonin syndrome (rare, but can be serious, especially when combined with other medications that increase serotonin levels)<br>13. Allergic reactions, including anaphylaxis (very rare)<br>As with any medication, it's important to use ondansetron under the guidance of a healthcare professional who can monitor for any adverse effects and adjust treatment as needed. If you experience any concerning symptoms while taking ondansetron, it's essential to contact your healthcare provider promptly. | |
| 7. Dexmedetomidine resp effects | **What are the respiratory effects of dexmedetomidine?**<br>**Response**: Dexmedetomidine is a selective alpha-2 adrenergic agonist primarily used for sedation in intensive care units (ICUs) and procedural sedation. Its respiratory effects differ from those of traditional sedatives like opioids and benzodiazepines.<br>1. **Respiratory Depression**: Dexmedetomidine typically causes minimal respiratory depression compared to other sedatives like opioids and benzodiazepines. This is one of its advantages, especially in patients who are at risk of respiratory compromise.<br>2. **Stable Respiratory Pattern**: Unlike opioids and benzodiazepines, dexmedetomidine usually maintains a stable respiratory pattern, with less impact on respiratory rate and tidal volume.<br>3. **Minimal Airway Obstruction**: Dexmedetomidine does not typically cause airway obstruction or compromise, making it suitable for sedation in patients who require an intact airway.<br>4. **Decreased Risk of Apnea**: While respiratory depression can occur with dexmedetomidine, the risk of apnea is lower compared to opioids and benzodiazepines. However, caution is still necessary, especially when used in combination with other sedatives or in patients with preexisting respiratory conditions. | 6 |

| | | | |
|---|---|---|---|
| | | Overall, dexmedetomidine is considered a favorable sedative option in critically ill patients due to its minimal respiratory depression and stable respiratory effects. However, individual patient factors and the specific clinical context should always be considered when choosing sedation agents. | |
| 8. | Factors enhancing inhalation induction | **How does adding nitrous oxide to the gas mix affect the speed of onset for inhalational induction of anesthesia?**<br>**Response:** Nitrous oxide is often used in combination with other inhalational anesthetics for induction of anesthesia. When added to the gas mix, nitrous oxide can increase the speed of onset for inhalational induction of anesthesia. This is primarily because nitrous oxide has a higher blood-gas partition coefficient compared to other inhalational anesthetics like sevoflurane or desflurane.<br><br>The blood-gas partition coefficient determines how readily a gas dissolves in the blood. A higher coefficient means the gas dissolves more readily, leading to faster equilibration between the alveoli in the lungs and the bloodstream. Nitrous oxide has a relatively high blood-gas partition coefficient, allowing it to rapidly enter the bloodstream and reach the brain, resulting in quicker onset of anesthesia compared to other inhalational agents.<br><br>Additionally, nitrous oxide has a unique property of being a weak anesthetic but a potent analgesic. This means it can provide some degree of pain relief even at sub-anesthetic concentrations, further facilitating the induction process.<br><br>However, it's essential to note that nitrous oxide alone is typically not used for complete anesthesia induction due to its weak anesthetic properties. It's commonly used as an adjunct to other inhalational or intravenous anesthetics to enhance their effects and provide analgesia. | 4 |
| 9. | IV anesthetics baroreceptor effects | **How do IV anesthetics affect the baroreceptor reflex?**<br>**Response**: Intravenous (IV) anesthetics can have varying effects on the baroreceptor reflex, which is responsible for regulating blood pressure in response to changes in blood volume and arterial pressure. The specific effects depend on the type and dosage of the anesthetic agent used.<br>Generally, IV anesthetics such as propofol, thiopental, and etomidate can depress the baroreceptor reflex to some extent. These agents exert their effects by acting on the central nervous system, particularly by enhancing the inhibitory neurotransmission mediated by gamma-aminobutyric acid (GABA). As a result, they can decrease sympathetic nervous system activity and dampen the reflexive responses to changes in blood pressure.<br>However, the degree of inhibition of the baroreceptor reflex may vary among different agents. For example, propofol is known to have a more pronounced inhibitory effect on sympathetic activity compared to other IV anesthetics. In contrast, ketamine, another IV anesthetic, may have less depressant | 6 |

| | | |
|---|---|---|
| | effects on the baroreceptor reflex due to its unique mechanism of action involving N-methyl-D-aspartate (NMDA) receptor antagonism.<br>It's important for anesthesiologists to consider these effects when administering IV anesthetics, especially in patients with compromised cardiovascular function or those undergoing procedures where blood pressure management is critical. Monitoring blood pressure and hemodynamic parameters closely during anesthesia is essential to ensure patient safety. | |
| 10. Opioid duration | **What's the duration of action of a single dose of 20 microgram per kilogram hydromorphone given intravenously under anesthesia?**<br>**Response:** The duration of action of a single dose of 20 micrograms per kilogram of hydromorphone given intravenously under anesthesia typically ranges from 2 to 4 hours. Hydromorphone is a potent opioid analgesic, and its effects, including analgesia, can be influenced by factors such as the patient's metabolic rate, the presence of anesthesia, and individual variability.<br><br>In a clinical setting, hydromorphone's peak effect occurs within 5 to 15 minutes after IV administration, and its half-life ranges from 2 to 3 hours. The duration of effective analgesia, however, may be somewhat shorter, and additional doses may be required to maintain pain control depending on the surgical procedure and patient response. Under anesthesia, the context-sensitive half-life may vary, but generally, the analgesic effect should last within the 2 to 4-hour window. | 6 |
| 11. Rocuronium elimination | **How is rocuronium eliminated?**<br>**Response**: Rocuronium, a non-depolarizing neuromuscular blocking agent, is primarily eliminated from the body through hepatic metabolism and renal excretion. The liver metabolizes it through Hofmann elimination and ester hydrolysis, converting it into inactive metabolites which are then eliminated via the kidneys. Around 40-50% of the administered dose is excreted unchanged in the urine within 24 hours. The elimination half-life of rocuronium is approximately 60-90 minutes in adults. | 4 |
| 12. ET tube selection | **What is the proper size internal diameter of an endotracheal tube for a 70-kilogram adult male?**<br>**Response:** The proper size internal diameter of an endotracheal tube for a 70-kilogram adult male typically ranges from 8.0 mm to 8.5 mm. The exact size may vary based on factors such as the individual's airway anatomy, medical condition, and the preferences of the healthcare provider performing the intubation. It's crucial to assess the patient's specific needs and consult with a medical professional for precise recommendations. | 6 |
| 13. RSI roc dose | **What is the dose of rocuronium for a rapid sequence intubation?**<br>**Response:** The dose of rocuronium for rapid sequence intubation (RSI) can vary depending on factors such as the patient's weight, medical condition, and other medications they may be taking. However, a common initial dose for rocuronium in RSI for an adult patient is typically around 0.6 to 1.2 milligrams | 4 |

| | | |
|---|---|---|
| | per kilogram (mg/kg) of body weight administered intravenously. This dose is usually administered quickly to facilitate rapid paralysis and intubation. It's crucial to consult with medical guidelines, protocols, and the patient's specific circumstances to determine the appropriate dosage. | |
| 14. RSI sux dose | **What is the dose of succinylcholine for a rapid sequence intubation?**<br>**Response** The typical dose of succinylcholine for rapid sequence intubation (RSI) in adults is 1-1.5 mg/kg IV. For pediatric patients, the dose is higher, usually around 2 mg/kg IV. However, dosing may vary based on factors such as the patient's medical condition, weight, and other medications they may be taking. It's crucial to consult the appropriate guidelines and protocols and to calculate the dosage accurately based on the patient's individual characteristics. Always ensure that administration is carried out by trained medical personnel in a controlled clinical setting. | 6 |
| 15. LR pH | **What is the pH of lactated ringers solution?**<br>**Response** Lactated Ringer's solution typically has a pH ranging from 6.0 to 7.5. However, the exact pH can vary slightly depending on the manufacturer and storage conditions. | 6 |
| 16. LR composition | **What is the potassium concentration of lactated ringers solution?**<br>**Response** The potassium concentration in lactated Ringer's solution typically ranges from 4 to 5 mEq/L (milliequivalents per liter). It's important to note that the exact concentration can vary slightly depending on the manufacturer and specific formulation of the solution. | 6 |
| 17. LR sodium | **What is the sodium concentration of lactated ringers solution?**<br>**Response** Lactated Ringer's solution typically contains approximately 130 mmol/L of sodium. This concentration helps to maintain electrolyte balance in the body during fluid replacement therapy. | 6 |
| 18. LR NS comparison | What electrolytes in lactated ringers solution are not present in normal saline?<br>**Response** Lactated Ringer's solution contains electrolytes that are not present in normal saline (0.9% sodium chloride). These additional electrolytes include:<br>1. **Potassium**: Lactated Ringer's solution contains potassium chloride, providing a source of potassium ions. Potassium is crucial for maintaining proper cellular function, nerve transmission, and muscle contraction.<br>2. **Calcium**: Lactated Ringer's solution contains calcium chloride, which adds calcium ions to the solution. Calcium is important for various physiological processes, including muscle contraction, nerve function, and blood clotting.<br>3. **Lactate**: Lactated Ringer's solution contains sodium lactate. Lactate is metabolized in the liver to bicarbonate, which helps buffer acidity in the blood. This can be beneficial in conditions where metabolic acidosis is a concern. | 6 |

| | | |
|---|---|---|
| | These additional electrolytes in Lactated Ringer's solution make it more physiologically balanced compared to normal saline and are especially useful in cases where electrolyte imbalances or metabolic acidosis are present. | |
| 19. Flumazenil indication | **What class of medications can be reversed with flumazenil?** Flumazenil is a medication used as a specific antidote for benzodiazepine overdose or to reverse the sedative effects of benzodiazepines after surgery. Benzodiazepines are a class of medications commonly used for their sedative, hypnotic, anxiolytic, anticonvulsant, and muscle relaxant properties. So, the class of medications that can be reversed with flumazenil is benzodiazepines. | 6 |
| 20. Sevoflurane MAC | **What is the minimum alveolar concentration of sevoflurane for a healthy 40 year old male?** The minimum alveolar concentration (MAC) of sevoflurane, like any anesthetic, can vary based on factors such as age, sex, weight, and overall health. However, for a healthy 40-year-old male, the approximate MAC of sevoflurane is typically around 2.0%. This means that at a concentration of 2.0% in the alveoli of the lungs, 50% of patients will not respond to surgical stimuli.<br>It's important to note that MAC values are not fixed and can vary among individuals and under different circumstances. They serve as guidelines for anesthesiologists to adjust dosages accordingly during surgeries and procedures. | 6 |
| 21. Baroreceptor reflex | **Will the heart rate increase or decrease with elevated blood pressure detected by the baroreceptor reflex?** When blood pressure increases, the baroreceptor reflex kicks in to help regulate it. This reflex involves sensors called baroreceptors, which are located in the walls of certain blood vessels, particularly in the carotid sinuses and the aortic arch. When these baroreceptors detect an increase in blood pressure, they send signals to the brain, specifically to the cardiovascular control center in the medulla oblongata.<br>In response to the increased blood pressure, the cardiovascular control center initiates parasympathetic nervous system activity and inhibits sympathetic nervous system activity. This leads to various physiological changes aimed at reducing blood pressure, including vasodilation (widening of blood vessels) and decreased heart rate (bradycardia).<br>So, when elevated blood pressure is detected by the baroreceptor reflex, the heart rate tends to decrease as part of the body's effort to regulate blood pressure and prevent it from rising too high. | 6 |
| 22. ASA classification | **What is the ASA Physical Status Classification of an adult with a body mass index (BMI) of 41kg/m² without additional comorbidities?** The ASA Physical Status Classification system, developed by the American Society of Anesthesiologists, categorizes a patient's overall health status prior to anesthesia or surgery. | 6 |

| | | |
|---|---|---|
| | An adult with a BMI of 41 kg/m² but without additional comorbidities would typically fall under ASA Physical Status Classification II. This classification is often described as a patient with mild to moderate systemic disease, but the disease does not limit daily activities.<br>It's worth noting that ASA classification takes into account various factors beyond just BMI, including medical conditions, functional capacity, and overall health status. So, the classification could vary based on individual circumstances. | |
| 23. ASA classification | **What is the ASA Physical Status Classification of an adult with a history of a stroke 45 days ago without additional comorbidities?**<br>The ASA Physical Status Classification (American Society of Anesthesiologists Physical Status Classification System) is a scale used to assess a patient's overall health before surgery or anesthesia. It ranges from ASA 1 (normal healthy patient) to ASA 6 (brain-dead patient).<br>A patient who had a stroke 45 days ago without additional comorbidities would typically fall under ASA 2 classification. ASA 2 signifies a patient with mild to moderate systemic disease, but not incapacitating. The stroke within the past two months would categorize them as having a recent significant medical event, warranting classification as ASA 2. | 3 |
| 24. Context sensitive half time | **What is the definition of context sensitive half time?**<br>The context-sensitive half-time (CSHT) is a pharmacokinetic parameter used to describe the time it takes for the plasma concentration of a drug to decrease by 50% following the termination of a continuous infusion at a constant rate. It provides valuable information about the duration of drug effect and the rate at which the drug is eliminated from the body under steady-state conditions.<br><br>The concept of context-sensitive half-time is particularly relevant for drugs with prolonged elimination half-lives, as it helps predict the time required for the drug concentration to decrease to half of its steady-state level after discontinuation of infusion. This parameter is especially important in clinical settings where prolonged infusions of medications are administered, such as during anesthesia or critical care, as it influences decisions regarding drug dosing and administration intervals.<br><br>The context-sensitive half-time is influenced by various factors, including the drug's pharmacokinetic properties (e.g., distribution volume, clearance), infusion duration, and the rate of elimination. Drugs with shorter context-sensitive half-times are generally cleared more rapidly from the body after infusion cessation, whereas drugs with longer context-sensitive half-times may exhibit prolonged effects even after discontinuation of infusion. | 6 |

| | | |
|---|---|---|
| | Clinicians use the context-sensitive half-time to optimize drug dosing strategies, minimize the risk of drug accumulation, and ensure timely recovery from drug effects. It provides valuable insights into the pharmacokinetic behavior of drugs during and after continuous infusion, helping to guide clinical decision-making and improve patient care. | |
| 25. La Place's law | **What is LaPlace's law?**<br>Laplace's law is a fundamental principle in physics and physiology that describes the relationship between the pressure inside a spherical structure, the tension in its wall (surface tension), and its radius. It's named after the French mathematician and physicist Pierre-Simon Laplace, who formulated it.<br><br>In simpler terms, Laplace's law states that the pressure inside a spherical structure is directly proportional to its surface tension and inversely proportional to its radius. Mathematically, it can be expressed as:<br><br>$P = 2T/r$<br><br>Where:<br>- P represents the pressure inside the sphere.<br>- T represents the surface tension of the material forming the sphere.<br>- r represents the radius of the sphere.<br><br>This law is applicable to various biological and physical systems. In medicine, it's relevant for understanding phenomena like blood vessel mechanics, alveolar mechanics in the lungs, and the behavior of bubbles in liquids. In engineering, it's used to design structures like pressure vessels and balloons, considering factors such as material strength and pressure. | 6 |
| 26. A-a gradient | **What is considered a normal A-a gradient?**<br>The alveolar-arterial (A-a) gradient is a measure of the difference in the partial pressure of oxygen (PaO2) between the alveoli of the lungs and the arterial blood. It reflects the efficiency of gas exchange in the lungs and can be used to assess the severity of ventilation-perfusion (V/Q) mismatch or diffusion impairment.<br><br>In a healthy individual breathing room air (FiO2 = 0.21), the A-a gradient typically ranges from 5 to 15 mmHg. This means that under normal conditions, there is a slight gradient between the partial pressure of oxygen in the alveoli and arterial blood due to minor physiological shunting and diffusion limitations. | 6 |

| | | |
|---|---|---|
| | However, it's essential to note that the normal range of the A-a gradient may vary slightly depending on factors such as age, altitude, and individual differences in lung function. Additionally, the A-a gradient tends to increase with age due to physiological changes in the lungs, such as decreased lung compliance and ventilation-perfusion matching.<br><br>Clinicians often use the A-a gradient to help diagnose and evaluate respiratory disorders such as pneumonia, pulmonary embolism, and acute respiratory distress syndrome (ARDS). A significantly elevated A-a gradient (greater than 25-30 mmHg) suggests significant impairment in gas exchange and may indicate the presence of a respiratory pathology that requires further evaluation and management. | |
| 27. 02 saturation/ paO2 relationship | **A SpO2 of 90% correlates to what PaO2?**<br>The correlation between peripheral oxygen saturation (SpO2) and arterial oxygen partial pressure (PaO2) is not exact and can vary depending on individual factors and clinical conditions. However, there are general approximations and relationships used in clinical practice to estimate PaO2 based on SpO2.<br><br>One commonly used approximation is the rule of thumb that each 1% decrease in SpO2 corresponds to approximately a 4 mmHg decrease in PaO2. Using this rule, we can estimate the PaO2 corresponding to an SpO2 of 90% as follows:<br><br>1. The normal PaO2 at sea level breathing room air (FiO2 = 0.21) is approximately 80-100 mmHg.<br>2. If each 1% decrease in SpO2 corresponds to approximately a 4 mmHg decrease in PaO2, a decrease of 10% (from 100% to 90%) corresponds to a decrease of approximately 40 mmHg in PaO2.<br>3. Therefore, if the normal PaO2 at sea level breathing room air is approximately 80-100 mmHg, a decrease of 40 mmHg would result in an estimated PaO2 of around 40-60 mmHg.<br><br>It's important to note that this is a rough approximation, and actual PaO2 values can vary based on factors such as altitude, patient age, lung function, and underlying medical conditions. Additionally, pulse oximetry (SpO2) provides only an estimate of arterial oxygen saturation and may not always accurately reflect PaO2, especially in certain clinical conditions. For precise assessment of oxygenation status and arterial blood gas levels, direct measurement of PaO2 via arterial blood gas (ABG) analysis is necessary. | 5 |
| 28. Interaction of inhalational anesth and NMB | **What is the interaction between inhalational anesthetic and neuromuscular blockade?**<br>**Response:** The interaction between inhalational anesthetics and neuromuscular blockade is complex and can vary depending on several factors, including the specific agents used, their doses, and the patient's individual characteristics. Here are some key points regarding this interaction: | 6 |

frontiers

| | | |
|---|---|---|
| | 1. **Potentiation of Neuromuscular Blockade:** Inhalational anesthetics can potentiate the effects of neuromuscular blocking agents (NMBAs), leading to enhanced neuromuscular blockade. This occurs through mechanisms such as potentiation of the postsynaptic effects of NMBAs and inhibition of neuromuscular transmission at the motor endplate.<br>2. **Dose-Dependent Effect:** The potentiating effect of inhalational anesthetics on neuromuscular blockade is dose-dependent, with higher concentrations of inhalational agents resulting in greater enhancement of neuromuscular blockade. Conversely, lower concentrations may have minimal effect on neuromuscular transmission.<br>3. **Choice of Inhalational Agent:** Different inhalational anesthetics may have varying effects on neuromuscular blockade. For example, volatile agents such as desflurane and sevoflurane are generally less potent in enhancing neuromuscular blockade compared to intravenous agents like propofol. However, all inhalational agents can contribute to some degree of potentiation of neuromuscular blockade.<br>4. **Clinical Implications:** The interaction between inhalational anesthetics and neuromuscular blockade has important clinical implications, particularly in the context of balanced anesthesia techniques. Anesthesiologists must carefully titrate the doses of both inhalational agents and NMBAs to achieve the desired level of anesthesia and muscle relaxation while minimizing the risk of respiratory compromise and delayed recovery.<br>5. **Monitoring:** Continuous monitoring of neuromuscular function using techniques such as train-of-four (TOF) stimulation is essential to assess the depth of neuromuscular blockade and guide the administration of NMBAs and reversal agents. This helps prevent excessive neuromuscular blockade and ensures timely recovery of neuromuscular function at the end of surgery.<br>Overall, the interaction between inhalational anesthetics and neuromuscular blockade is an important consideration in anesthesia management, requiring careful titration of both agents to achieve optimal anesthesia and muscle relaxation while minimizing adverse effects. Close monitoring and individualized management strategies are essential to ensure patient safety and optimize outcomes. | |
| 29. ESU burns | **A patient who has a left total hip replacement is undergoing open abdominal surgery, where should the return pad of the electrosurgical unit be placed?**<br>**Response:** For a patient with a left total hip replacement undergoing open abdominal surgery, the return pad of the electrosurgical unit (ESU) should be placed on a well-vascularized, large, and muscular area, ideally far from the prosthetic implant to avoid any electrical interference or potential damage to the implant.<br>Here are the steps and considerations for proper placement: | 6 |

| | | |
|---|---|---|
| | **Avoid the Operative Site and Implant:** The return pad should not be placed near the surgical site (abdomen) or the area of the hip replacement to minimize the risk of electrical interference and potential heating of the prosthetic components.<br>**Optimal Placement Areas:** Suitable areas include the thigh (opposite to the side of the hip replacement, if possible), the buttocks (opposite side), or the back. These areas provide large muscle masses with good vascularity, ensuring effective current dispersion.<br>**Skin Integrity and Condition:** Ensure the skin where the pad is placed is intact, free from any lotions or ointments, and clean to ensure good adhesion and minimize the risk of burns.<br>In this specific case, considering the left hip replacement, a good placement option would be the right thigh or right buttock, ensuring that the path of the electrical current does not pass through the prosthetic hip. If both thighs or buttocks are suitable, the right side is generally preferable to avoid the left hip area entirely.<br>Key Points:<br>Place the return pad away from the prosthetic implant and the surgical site.<br>Use large, well-vascularized, and muscular areas.<br>Ensure the skin is clean and intact for optimal contact and safety.<br>Thus, for this patient, the right thigh or right buttock would be the most appropriate locations for the return pad of the electrosurgical unit. | |
| 30. Laryngospasm treatment | **After anesthesia, patient developed laryngospasm which was refractory to selicks maneuver and PEEP, pulse oximetry is reading 70%, what drug should be administered?**<br>**Response:** In the scenario where a patient develops laryngospasm after anesthesia that is refractory to the Sellick's maneuver and Positive End-Expiratory Pressure (PEEP), and the pulse oximetry reading is critically low at 70%, the recommended drug to administer is succinylcholine.<br><br>Succinylcholine is a rapid-onset, short-acting neuromuscular blocking agent that can quickly relax the laryngeal muscles, allowing for the resolution of laryngospasm. The typical dose for this situation is 0.1 to 0.2 mg/kg IV. This should rapidly improve the patient's ability to oxygenate by alleviating the spasm and allowing for effective ventilation.<br><br>Here are the steps to follow:<br><br>Administer Succinylcholine: 0.1 to 0.2 mg/kg IV to achieve rapid muscle relaxation.<br>Provide Adequate Oxygenation: Continue providing 100% oxygen and ensure proper airway management.<br>Prepare for Further Airway Management: Be ready to perform advanced airway interventions if the patient's condition does not improve immediately. | 6 |

| | | |
|---|---|---|
| | It's also important to have resuscitation equipment and emergency medications readily available whenever managing a patient under anesthesia. | |
| 31. Side effects of normal saline infusion | **What biochemical change is most commonly associated with large volume normal saline infusion?**<br>**Response:** The most common biochemical change associated with large volume normal saline (0.9% sodium chloride) infusion is hyperchloremic metabolic acidosis. This occurs due to the high chloride content of normal saline relative to plasma. When large volumes are infused, the increased chloride load can lead to a relative excess of chloride ions in the blood, which can decrease the plasma bicarbonate concentration through a dilutional effect and by shifting the bicarbonate-chloride equilibrium, leading to metabolic acidosis.<br><br>Key points to consider:<br><br>Chloride Load: Normal saline has a higher chloride concentration (154 mEq/L) compared to plasma (approximately 100-106 mEq/L).<br>Metabolic Acidosis: The infusion of large volumes of normal saline can lead to a non-anion gap metabolic acidosis due to the increase in chloride ions.<br>Electrolyte Imbalance: The acidosis is often described as hyperchloremic because the primary change is an increase in chloride ions, rather than an increase in organic acids.<br>Clinicians should monitor patients receiving large volumes of normal saline for signs of this biochemical imbalance and consider using balanced crystalloids (like lactated Ringer's or Plasma-Lyte) in situations where large volume resuscitation is anticipated to avoid this complication. | 6 |
| 32. Calculation of epinephrine conc | **What volume of 1mg/ml epinephrine should be added to a bag of 100ml normal saline to achieve a final concentration of 16 microgram per ml?**<br>**Response:** To find out the volume of a 1 mg/ml epinephrine solution that should be added to 100 ml of normal saline to achieve a final concentration of 16 µg/ml, we can use the following steps:<br><br>Determine the total amount of epinephrine needed:<br><br>We want a final concentration of 16 µg/ml in 100 ml of solution.<br>The total amount of epinephrine required in the final solution is:<br>16 µg/ml ×100 ml=1600 µg<br>16µg/ml×100ml= 1600µg<br>Convert micrograms to milligrams:<br><br>Since 1 mg = 1000 µg, we convert 1600 µg to mg: | 6 |

| | | |
|---|---|---|
| | 1600 µg=1600 ÷ 1000mg =1.6mg<br><br>Determine the volume of 1 mg/ml epinephrine solution needed:<br><br>The concentration of the epinephrine solution is 1 mg/ml.<br>To find the volume (V) that contains 1.6 mg of epinephrine, we use the formula:<br>Volume=Amount of Epinephrine ÷ Concentration=1.6mg ÷1mg/ml=1.6 ml<br><br>So, you need to add 1.6 ml of the 1 mg/ml epinephrine solution to the 100 ml bag of normal saline to achieve a final concentration of 16 µg/ml. | |
| 33. Induction drugs, duration of action | **What's the duration of action of a single dose of 2 mg/kg propofol given intravenously for the induction of anesthesia?**<br>**Response:** The duration of action of a single dose of 2 mg/kg of propofol given intravenously for the induction of anesthesia typically ranges from 5 to 10 minutes. This is the time during which the patient remains adequately anesthetized for procedures following the initial induction. Propofol is known for its rapid onset and short duration of action, which is why it is commonly used for the induction and maintenance of anesthesia. The precise duration can vary based on individual factors such as the patient's age, weight, and overall health, as well as concurrent medications and the specific context of its use. | 6 |
| 34. Anatomy of epidural space | **When placing an epidural catheter, what is the last layer of structure the Tuohy needle passes before entry to the epidural space?**<br>**Response:** The last layer of structure the Touhy needle passes through before entering the epidural space is the ligamentum flavum. This ligament is a strong, elastic band of tissue that connects adjacent vertebrae in the spine. It forms the posterior wall of the epidural space and must be punctured by the needle to access the epidural space for catheter placement. | 6 |
| 35. Opioid elimination | **How is remifentanil eliminated in vivo?**<br>**Response:** Remifentanil is primarily eliminated from the body through metabolism by non-specific tissue and plasma esterases. These enzymes break down remifentanil into inactive metabolites, including remifentanil acid. Unlike many other opioids, remifentanil's metabolism is not dependent on hepatic function, making it suitable for patients with impaired liver function. The rapid metabolism of remifentanil contributes to its short duration of action, which is desirable in certain clinical situations. | 6 |
| 36. 02 cylinder content | **What is the volume of oxygen inside an E cylinder at 1100 psi?**<br>**Response:** To calculate the volume of oxygen in an E cylinder at 1100 psi, you need to know the volume of the cylinder when it's filled to that pressure. The volume of an E cylinder can vary slightly depending on the manufacturer, but it's typically around 660 liters when filled to a pressure of 2200 psi, which is its standard operating pressure. | 4 |

| | | |
|---|---|---|
| | To find the volume at 1100 psi, you can use the ideal gas law:<br>PV=nRT<br><br>Where:<br>P is the pressure (in atmospheres)<br>V is the volume (in liters)<br>n is the number of moles of gas<br>R is the ideal gas constant (0.0821 L atm / mol K)<br>T is the temperature (in Kelvin)<br><br>Since you're only interested in the change in pressure and volume, and assuming the temperature remains constant, you can simplify the equation to:<br><br>$P1V1=P2V2$<br>Where:<br>P1 is the initial pressure<br>V1 is the initial volume<br>P2 is the final pressure<br>V2 is the final volume<br>Let's solve for<br>V2 (the volume at 1100 psi):<br>$V2=P1V1P2\div$ V2<br>Given:<br>P1 =2200psi<br>V1=660liters<br>P2=1100psi<br>$V2$= 2200psi×660liters÷1100psi=1452000÷1100≈1320 liters<br>So, the volume of oxygen inside the E cylinder at 1100 psi would be approximately 1320 liters. | |
| 37. Effects of preop smoking cessation | **How long after stopping smoking does carboxyhemoglobin level normalize?**<br>**Response:** Carboxyhemoglobin is formed when carbon monoxide (CO) from tobacco smoke binds to hemoglobin in the blood, reducing its ability to carry oxygen. The half-life of carboxyhemoglobin in the blood—the time it takes for half of it to be eliminated—varies depending on factors like the concentration of CO in the blood and individual differences in metabolism.<br><br>Generally, for a non-smoker, it takes about 4 to 6 hours for carboxyhemoglobin levels to decrease by half after exposure to CO. However, in smokers, it can take much longer due to chronic exposure to CO. | 6 |

| | | |
|---|---|---|
| | After quitting smoking, carboxyhemoglobin levels typically start to decrease rapidly, and within 24 hours, they can drop significantly. It can take several weeks for carboxyhemoglobin levels to return to those of a non-smoker, but this timeframe can vary from person to person. Regular physical activity and good overall health can also contribute to faster elimination of carboxyhemoglobin from the body. | |
| 38. Periop anaphylaxis | **What's the most common cause of drug induced anaphylaxis in the perioperative setting?** **Response:** In the perioperative setting, the most common cause of drug-induced anaphylaxis is thought to be muscle relaxants, particularly agents like succinylcholine and rocuronium. These drugs are frequently used during surgery to induce muscle relaxation and facilitate intubation. However, it's important to note that anaphylaxis can occur with a variety of medications used in the perioperative period, including antibiotics, anesthetic agents, and other drugs. Prompt recognition and treatment are crucial in managing anaphylactic reactions in this setting. | 3 |
| 39. Abnormal capnograph patterns | **What capnograph pattern is strongly suggestive of malignant hyperthermia?** **Response:** In malignant hyperthermia (MH), a rare but potentially life-threatening condition triggered by certain drugs used during anesthesia, the capnograph often shows an elevated baseline (end-tidal carbon dioxide, or ETCO2), rapid and increasing ETCO2 levels, and a sudden and sustained rise in ETCO2 despite constant or increasing ventilation. This pattern is often referred to as a "shark fin" or "sawtooth" pattern due to its appearance on the capnograph waveform. This abnormal capnograph pattern is strongly suggestive of MH and should prompt immediate cessation of triggering agents and initiation of appropriate treatment. | 5 |
| 40. Interpretation of blood gases | **Can you interpret the following ABG: pH 7.25, pO2 165mmHg on 50% FiO2, pCO2 42, Base excess -5** <br>**Response:** Certainly! Let's break down the components of this arterial blood gas (ABG) result: <br>pH 7.25: This indicates the acidity of the blood. A pH below 7.35 is considered acidic. In this case, the blood is slightly acidic, which could indicate respiratory or metabolic acidosis. <br>pO2 165mmHg on 50% FiO2: The partial pressure of oxygen (pO2) is 165 mmHg. This is slightly elevated, which is unusual when considering the patient is receiving 50% fraction of inspired oxygen (FiO2). It could suggest some degree of impaired oxygen diffusion or shunting, or possibly hyperventilation. However, without knowing the patient's clinical context, it's hard to draw firm conclusions. <br>pCO2 42: The partial pressure of carbon dioxide (pCO2) is within normal range (35-45 mmHg). This suggests normal respiratory function in terms of eliminating CO2. <br>Base excess -5: Base excess (BE) is a measure of the amount of excess or insufficient level of bicarbonate in the blood. A negative base excess indicates metabolic acidosis, suggesting there may be an excess of acids or a loss of bases in the blood. <br>Overall interpretation: The blood is slightly acidic with a pH of 7.25, possibly due to a combination of respiratory alkalosis (elevated pO2) and metabolic acidosis (negative base excess). The normal pCO2 | 3 |

| | | |
|---|---|---|
| | suggests the respiratory system is compensating for the metabolic acidosis to some extent. This ABG suggests a complex acid-base disturbance, which would require further clinical correlation and investigation to determine the underlying cause and appropriate management. | |

Extended response prompts (30 prompts):

| Q. number & Keyword | Prompts and responses | Accuracy | Complete-ness |
|---|---|---|---|
| 1. Endobronchial intubation effects | **What are the effects of endobronchial intubation?**<br>**Response**: Endobronchial intubation refers to the placement of an endotracheal tube (ETT) into one of the mainstem bronchi instead of the trachea, which is the intended placement. This can lead to several effects, including:<br>1. **Hypoxemia**: Oxygenation may be compromised because the majority of the inspired gas enters only one lung instead of both lungs. This can result in decreased oxygen saturation in the blood.<br>2. **Atelectasis**: The lung that is not receiving ventilation may collapse partially or completely due to lack of airflow, leading to atelectasis, a condition characterized by the collapse of lung tissue.<br>3. **Ventilation-Perfusion Mismatch**: Blood flow to the non-ventilated lung continues, creating a ventilation-perfusion mismatch, which impairs gas exchange efficiency.<br>4. **Altered Breath Sounds**: Physical examination may reveal absent or decreased breath sounds on the side of the lung that is not ventilated.<br>5. **Hemodynamic Effects**: The mismatch between ventilation and perfusion can lead to changes in pulmonary vascular resistance and potentially affect hemodynamics.<br>6. **Barotrauma**: The lung that is receiving all the ventilation may be exposed to higher pressures than normal, increasing the risk of barotrauma, such as pneumothorax or pneumomediastinum.<br>7. **Unilateral Pulmonary Edema**: Prolonged endobronchial intubation can lead to unilateral pulmonary edema due to increased hydrostatic pressure in the ventilated lung. | 6 | 6 |

| | | | |
|---|---|---|---|
| | 8. **Delayed Diagnosis and Treatment**: If not promptly recognized and corrected, endobronchial intubation can delay appropriate treatment and worsen patient outcomes.<br>Prompt identification and correction of endobronchial intubation are crucial to prevent complications and ensure adequate ventilation and oxygenation. This is typically done by confirming proper tube placement via chest X-ray, auscultation, capnography, and other methods. | | |
| 2. Warfarin reversal | **What is the treatment for warfarin anticoagulation reversal?**<br>**Response:** The reversal of warfarin anticoagulation is typically required in situations of bleeding or when urgent surgery is necessary. Several options are available for reversing the anticoagulant effects of warfarin:<br>1. **Vitamin K**: This is the primary agent used for warfarin reversal. It promotes the synthesis of clotting factors II, VII, IX, and X in the liver. Intravenous or oral administration of vitamin K can be used depending on the urgency of reversal.<br>2. **Fresh Frozen Plasma (FFP)**: FFP contains all coagulation factors and can rapidly reverse the effects of warfarin. It's typically used in cases of severe bleeding or when urgent surgery is required.<br>3. **Prothrombin Complex Concentrate (PCC)**: PCC contains concentrated forms of clotting factors II, VII, IX, and X. It's preferred over FFP in some situations because it can be administered more quickly and in smaller volumes. There are different types of PCCs available, such as 3-factor PCC and 4-factor PCC, with the latter being more effective due to its inclusion of factor VII.<br>4. **Recombinant Factor VIIa (rFVIIa)**: This is a synthetic form of factor VIIa that can promote clot formation independently of other clotting factors. It's typically reserved for cases where other reversal agents are ineffective or unavailable.<br>5. **Prothrombin Complex Concentrate (PCC)**: PCC contains concentrated forms of clotting factors II, VII, IX, and X. It's preferred over FFP in some situations because it can be administered more quickly and in smaller volumes. There are different types of PCCs available, such as 3-factor PCC and 4-factor PCC, with the latter being more effective due to its inclusion of factor VII.<br>The choice of reversal agent depends on various factors such as the severity of bleeding, the patient's underlying medical conditions, and the urgency of the situation. It's important to consult with a healthcare professional for individualized management. | 5 | 6 |

| | | | |
|---|---|---|---|
| 3. Drug dosing in morbid obesity | **How is anesthetic drug dosing affected by obesity?**<br>**Response**: Obesity can significantly impact the dosing of anesthetic drugs due to various physiological changes in individuals with obesity. Here are some factors to consider:<br>1. **Pharmacokinetics**: Many anesthetic drugs are lipophilic, meaning they distribute into fatty tissue. In obesity, where there's an increased amount of adipose tissue, the volume of distribution for these drugs may be larger, necessitating higher doses to achieve the desired effect.<br>2. **Cardiovascular Changes**: Obesity is often associated with cardiovascular comorbidities such as hypertension and impaired cardiac function. Anesthetic drugs can affect cardiovascular function, so dosing must be adjusted to minimize adverse effects on the heart and blood vessels.<br>3. **Renal and Hepatic Function**: Obesity can affect renal and hepatic function, altering the metabolism and excretion of anesthetic drugs. Adjustments may be needed to account for impaired clearance in obese individuals.<br>4. **Airway Management**: Obesity is frequently associated with anatomical changes that can complicate airway management during anesthesia. This might influence the choice of anesthetic agents and dosing strategies to ensure proper ventilation and oxygenation.<br>5. **Monitoring**: Close monitoring of obese patients during anesthesia is crucial due to the increased risk of complications. This includes monitoring vital signs, depth of anesthesia, and neuromuscular function to optimize dosing and maintain patient safety.<br>Overall, individualized dosing based on factors such as total body weight, lean body mass, and comorbidities is essential when administering anesthesia to obese patients to ensure both efficacy and safety. Collaboration between anesthesiologists, surgeons, and other healthcare providers is vital in optimizing perioperative care for obese individuals. | 6 | 5 |
| 4. LMA vs ETT | **What is the benefit of placement of endotracheal tube over a supraglottic airway?**<br>**Response:** The choice between an endotracheal tube (ETT) and a supraglottic airway device (SAD) depends on various factors including the patient's condition, the procedure being performed, and the expertise of the healthcare provider.<br>Benefits of placing an endotracheal tube over a supraglottic airway include:<br>1. **Secure Airway**: ETT provides a secure and definitive airway by directly entering the trachea, minimizing the risk of aspiration and ensuring adequate oxygenation and ventilation. | 5 | 6 |

| | | | |
|---|---|---|---|
| | 2. **Controlled Ventilation**: With an ETT, healthcare providers have greater control over ventilation parameters such as tidal volume, respiratory rate, and oxygen concentration.<br>3. **Protection Against Gastric Aspiration**: Endotracheal intubation provides a seal between the trachea and esophagus, reducing the risk of regurgitation and aspiration of gastric contents into the lungs.<br>4. **Facilitates Suctioning and Bronchoscopy**: ETT allows for effective suctioning of secretions and the passage of bronchoscopes for airway inspection and clearance.<br>5. **Ideal for Prolonged Ventilation**: In cases where prolonged mechanical ventilation is needed, an ETT is often preferred due to its stability and long-term tolerability.<br>However, it's essential to consider that endotracheal intubation requires advanced skills and may not be suitable for all situations or patients, especially in out-of-hospital settings or when performed by less experienced providers. Supraglottic airway devices, such as the laryngeal mask airway (LMA), are simpler to insert and can be valuable alternatives in certain circumstances, such as during rapid sequence induction or when managing difficult airways. They can also serve as a bridge to definitive airway management or facilitate ventilation during short procedures. | | |
| 5. LMA contraindication | **What are the contraindications to a supraglottic airway?**<br>**Response**: There are several contraindications to using a supraglottic airway device. These contraindications may vary slightly depending on the specific type of supraglottic device being used and the patient's condition, but some common contraindications include:<br>1. Inability to protect the airway: If the patient is unable to maintain their own airway or protect it from aspiration (e.g., due to reduced level of consciousness or impaired airway reflexes), using a supraglottic airway may not be appropriate.<br>2. Upper airway obstruction: If there is a significant obstruction above the level of the glottis (such as massive oropharyngeal bleeding, severe edema, or a foreign body), inserting a supraglottic airway may not effectively establish a patent airway.<br>3. Anatomical abnormalities: Certain anatomical abnormalities or pathologies of the upper airway may make insertion of a supraglottic airway difficult or unsafe. These may include significant facial trauma, severe burns to the upper airway, or tumors obstructing the airway. | 4 | 4 |

| | | | |
|---|---|---|---|
| | 4. Full stomach: In cases where there is a risk of regurgitation and aspiration of gastric contents (e.g., patients with a full stomach, bowel obstruction, or ileus), using a supraglottic airway may increase the risk of aspiration pneumonia.<br>5. Risk of cervical spine injury: If there is a suspected or known cervical spine injury, the manipulation required to insert a supraglottic airway device may exacerbate spinal cord damage. In such cases, other airway management techniques may be preferred.<br>6. Lack of training or experience: Inserting and managing a supraglottic airway device requires specific training and expertise. If the healthcare provider is not adequately trained or experienced in the use of supraglottic airways, it may be safer to use alternative airway management techniques.<br>It's important for healthcare providers to carefully assess each patient's condition and consider these contraindications before deciding on the appropriate airway management approach. | | |
| 6. Preoxygenation | **Please calculate the safe apneic time of an healthy 70kg adult with an end tidal O2 of 90%?**<br>The safe apneic time, or the duration for which a person can safely hold their breath without significant risk of hypoxemia (low oxygen levels), can be estimated using various factors including the initial oxygen reserves in the body and the rate of oxygen consumption.<br>Here's a step-by-step outline to calculate the safe apneic time for a healthy 70 kg adult with an end-tidal O2 of 90%:<br>Oxygen Consumption Rate (VO2):<br>A typical resting oxygen consumption rate is about 3-4 mL/kg/min.<br>For a 70 kg adult, this translates to approximately 210-280 mL/min.<br>Oxygen Reserves in the Body:<br>Lungs: The functional residual capacity (FRC) of the lungs in a 70 kg adult is about 2.5 liters. With an end-tidal O2 of 90%, the amount of oxygen in the lungs can be estimated as 2.5 liters * 90% = 2.25 liters or 2250 mL.<br>Blood: The oxygen content in the blood is determined by the hemoglobin concentration and the oxygen saturation. Assuming a hemoglobin concentration of 15 g/dL and full oxygen saturation (100%), the oxygen content is approximately 20 mL O2/100 mL blood. The total blood volume for a 70 kg adult is around 5 liters, so the total oxygen content in the blood is 20 mL/100 mL * 5000 mL = 1000 mL.<br>Tissues: Oxygen dissolved in tissues is relatively minimal but should be considered. The oxygen reserve in the tissues is roughly estimated as 50 mL. | 4 | 4 |

| | | | |
|---|---|---|---|
| | Total Oxygen Reserves:<br>Combining the oxygen in the lungs, blood, and tissues:<br>$2250 mL (lungs) + 1000 mL (blood) + 50 mL (tissues) = 3300 mL$<br><br>Calculating Safe Apneic Time:<br>Using the higher end of the oxygen consumption rate for safety (280 mL/min):<br>Safe Apneic Time= Total Oxygen Reserves ÷ Oxygen Consumption Rate<br>Safe Apneic Time=3300 mL ÷280 mL/min≈11.79 minutes<br>Therefore, the estimated safe apneic time for a healthy 70 kg adult with an end-tidal O2 of 90% is approximately 11.8 minutes. This is an estimation and individual variations could affect the actual safe apneic time. Factors such as activity level, metabolic rate, and overall health condition should also be considered. | | |
| 7. Epidural test dose | **What physiologic effects are expected if an epidural test dose of lidocaine 1.5% with epinephrine 1:200,000 was injected into the intravascular space?**<br>Injecting an epidural test dose of lidocaine 1.5% with epinephrine 1:200,000 into the intravascular space can lead to several physiological effects due to the systemic absorption of both lidocaine and epinephrine:<br>1. **Cardiovascular Effects**:<br>- Epinephrine can cause an increase in heart rate (tachycardia) and blood pressure due to its sympathomimetic effects on the heart and blood vessels.<br>- Lidocaine can cause myocardial depression and vasodilation at higher doses, but if rapidly absorbed in large quantities, it can also lead to systemic toxicity, potentially causing arrhythmias, hypotension, and cardiac arrest.<br>2. **Central Nervous System Effects**:<br>- Lidocaine, when present in the systemic circulation at high concentrations, can cause CNS toxicity, resulting in symptoms such as dizziness, confusion, seizures, and potentially coma.<br>3. **Respiratory Effects**:<br>- Lidocaine toxicity can also affect the respiratory system, potentially leading to respiratory depression and even respiratory arrest.<br>4. **Neurological Effects**:<br>- Lidocaine can cause neurological symptoms such as numbness, tingling, and muscle twitching, which may progress to seizures in cases of severe toxicity.<br>5. **Local Anesthetic Systemic Toxicity (LAST)**: | 5 | 5 |

| | | | |
|---|---|---|---|
| | - The systemic absorption of lidocaine from an intravascular injection can lead to local anesthetic systemic toxicity, which can manifest as a combination of cardiovascular, CNS, and respiratory effects.<br>6. **Vasoconstrictive Effects**:<br>- Epinephrine, being a vasoconstrictor, can lead to peripheral vasoconstriction, potentially causing tissue ischemia and necrosis if the injection occurs in an area with poor collateral circulation.<br>In summary, injecting an epidural test dose of lidocaine 1.5% with epinephrine 1:200,000 into the intravascular space can result in a range of systemic effects, including cardiovascular stimulation, CNS toxicity, respiratory depression, and potential local tissue damage. Immediate recognition and management of systemic toxicity are crucial to prevent severe complications. | | |
| 8. Benzo overdose | **What is the signs and symptoms of benzodiazepine overdose?**<br>**Response:** Benzodiazepine overdose can manifest with a range of signs and symptoms, which can vary depending on factors such as the specific benzodiazepine involved, the dosage taken, and whether other substances were also ingested. Here are some common signs and symptoms:<br>1. **Sedation and drowsiness:** Excessive sleepiness or difficulty staying awake.<br>2. **Confusion and disorientation:** Impaired cognitive function, confusion, and difficulty thinking clearly.<br>3. **Muscle weakness:** Weakness or loss of muscle control.<br>4. **Slurred speech:** Difficulty speaking clearly and coherently.<br>5. **Slow breathing:** Respiratory depression, characterized by slow or shallow breathing.<br>6. **Blurred vision:** Difficulty focusing vision or seeing clearly.<br>7. **Low blood pressure:** Hypotension, which can lead to dizziness or fainting.<br>8. **Weak pulse:** A slow or weak pulse rate.<br>9. **Hypotonia:** Reduced muscle tone or flaccidity.<br>10. **Coma:** In severe cases, overdose can lead to unconsciousness and coma.<br>It's important to seek medical attention immediately if someone is suspected of having overdosed on benzodiazepines, as overdose can be life-threatening, especially when combined with other substances such as alcohol or opioids. | 6 | 6 |
| 9. Transfusion reactions | **Tell me about the types of transfusion reactions** | 6 | 6 |

| | | | |
|---|---|---|---|
| | **Response:** Transfusion reactions are adverse events that can occur in response to the transfusion of blood or blood products. These reactions can range from mild to severe and may occur due to various mechanisms, including immune-mediated responses, non-immune reactions, and infectious complications. Here are the main types of transfusion reactions:<br><br>1. **Acute Hemolytic Transfusion Reaction (AHTR):** AHTR is a severe and potentially life-threatening immune-mediated reaction characterized by the rapid destruction of transfused red blood cells (RBCs). It occurs when the recipient's immune system recognizes donor RBC antigens as foreign and mounts an immune response, leading to hemolysis. AHTR can result from ABO incompatibility, Rh incompatibility, or other blood group mismatches. Symptoms may include fever, chills, flank pain, hemoglobinuria, hypotension, and disseminated intravascular coagulation (DIC).<br><br>2. **Febrile Non-Hemolytic Transfusion Reaction (FNHTR):** FNHTR is a relatively common transfusion reaction characterized by fever (temperature rise of ≥1°C) without evidence of hemolysis. It typically occurs due to recipient antibodies reacting to donor leukocytes or cytokines released during blood component processing and storage. Symptoms may include fever, chills, and rigors, and typically resolve spontaneously without specific treatment.<br><br>3. **Allergic Transfusion Reaction:** Allergic reactions are caused by recipient antibodies reacting to allergens in the transfused blood components, such as plasma proteins or donor IgA. Symptoms may include hives, itching, flushing, wheezing, and, rarely, anaphylaxis. Mild reactions can be managed with antihistamines, while severe reactions may require treatment with epinephrine and discontinuation of the transfusion.<br><br>4. **Transfusion-Related Acute Lung Injury (TRALI):** TRALI is a severe transfusion reaction characterized by acute respiratory distress and non-cardiogenic pulmonary edema within 6 hours of transfusion. It is caused by donor antibodies (usually anti-leukocyte antibodies) reacting with recipient leukocytes, leading to neutrophil activation and endothelial damage in the lungs. Management involves respiratory support and discontinuation of the transfusion. | | |

| | | | |
|---|---|---|---|
| | 5. **Transfusion-Associated Circulatory Overload (TACO):** TACO occurs when the volume of transfused blood components exceeds the recipient's circulatory capacity, leading to fluid overload and congestive heart failure. Symptoms may include dyspnea, orthopnea, hypertension, and pulmonary edema. Management involves diuresis, oxygen therapy, and, in severe cases, mechanical ventilation.<br><br>6. **Delayed Hemolytic Transfusion Reaction (DHTR):** DHTR is a delayed immune-mediated reaction characterized by the destruction of transfused RBCs by recipient antibodies days to weeks after transfusion. It can occur in patients with a history of alloimmunization (e.g., previous transfusions or pregnancy) and may present with anemia, jaundice, and hemoglobinuria.<br><br>7. **Infectious Complications:** Transfusion-related infections, including bacterial contamination of blood components and transmission of viral, bacterial, or parasitic pathogens, can occur despite strict screening and testing protocols. Examples include transfusion-transmitted infections like HIV, hepatitis B and C, and bacterial sepsis.<br><br>Prompt recognition and management of transfusion reactions are essential to minimize morbidity and mortality associated with transfusion-related complications. Healthcare providers should be vigilant in monitoring patients during transfusions and promptly report suspected reactions to transfusion services for investigation and management. | | |
| 10. Difficult airway | **Tell me about the algorithm to manage a difficult airway**<br>**Response:** Managing difficult airways is a critical aspect of anesthesia and emergency medicine. An algorithmic approach helps guide clinicians in assessing and managing difficult airway situations systematically. One commonly used algorithm is the Difficult Airway Society (DAS) guidelines, which provide a structured approach to airway management. Here's an overview of the DAS algorithm:<br>**Assessment:** The initial step is to assess the patient's airway using a systematic approach, including history, examination, and airway evaluation (e.g., Mallampati score, thyromental distance, mouth opening, neck mobility). Identify potential predictors of difficult intubation or ventilation.<br>2. **Preoxygenation:** Ensure adequate preoxygenation to increase the duration of safe apnea and minimize the risk of desaturation during airway management. Use techniques such as the "4-4-4" rule (4 deep breaths, 4 liters per minute of oxygen flow, for 4 minutes) or non-invasive ventilation as appropriate. | 6 | 5 |

| | | | |
|---|---|---|---|
| | 3. **Plan A, B, C, and D:** Develop a stepwise plan for airway management based on the assessment findings:<br>**Plan A:** Attempt direct laryngoscopy and tracheal intubation using standard techniques. Ensure appropriate positioning and use adjuncts (e.g., stylets, bougies) if needed.<br>**Plan B:** If Plan A fails or is predicted to fail, proceed to a second-line technique such as video laryngoscopy, supraglottic airway device (e.g., laryngeal mask airway), or awake fiberoptic intubation.<br>**Plan C:** If Plan B fails or is not feasible, consider invasive airway access techniques such as cricothyroidotomy or emergency front of neck access (eFONA).<br>**Plan D:** As a last resort, if all other options fail, perform emergency surgical airway (cricothyroidotomy or tracheostomy) or use rescue devices like the Cook Airway Exchange Catheter (AEC).<br>4. **Communication and Teamwork:** Ensure effective communication and coordination among team members involved in airway management. Assign specific roles and responsibilities, anticipate difficulties, and have backup plans in place.<br>5. **Monitoring and Reassessment:** Continuously monitor the patient's oxygenation, ventilation, and hemodynamic status during airway management. Be prepared to reassess and modify the airway plan based on the patient's response and clinical course.<br>6. **Debriefing and Documentation:** After airway management, conduct a debriefing session to review the case, identify areas for improvement, and provide feedback to team members. Document the airway assessment, interventions performed, and patient response in the medical record.<br>It's important to emphasize that airway management algorithms serve as guides rather than rigid protocols. Clinicians should individualize their approach based on the patient's clinical condition, available resources, and their own expertise. Regular training and simulation exercises can help improve team readiness and confidence in managing difficult airway situations. | | |
| 11. Des Vaporizer | **Tell me about the differences seen with a desflurane vaporizer**<br>**Response:** Desflurane is a volatile anesthetic agent commonly used for maintenance of general anesthesia. Desflurane vaporizers are specifically designed to deliver desflurane in a precise and controlled manner. Here are some key differences and characteristics associated with desflurane vaporizers compared to other vaporizers: | 6 | 6 |

| | | | |
|---|---|---|---|
| | 1. **Temperature Sensitivity:** Desflurane has a higher boiling point (23.5°C) compared to other volatile anesthetics like isoflurane and sevoflurane. As a result, desflurane vaporizers require a higher temperature to vaporize the liquid desflurane into a gaseous form suitable for inhalation. Desflurane vaporizers are equipped with a heated vaporizing chamber to maintain the appropriate temperature for vaporization.<br><br>2. **Vapor Pressure:** Desflurane has a high vapor pressure at room temperature, making it more volatile and prone to rapid changes in vapor concentration compared to other volatile anesthetics. Desflurane vaporizers are designed to deliver desflurane at a precise vapor concentration despite its high vapor pressure, ensuring consistent anesthetic depth during maintenance of anesthesia.<br><br>3. **Flow Characteristics:** Desflurane vaporizers have unique flow characteristics to optimize vapor delivery. Desflurane is less soluble in blood and tissues compared to other volatile agents, resulting in a faster onset and offset of action. Desflurane vaporizers are designed to deliver desflurane efficiently at high flow rates to achieve the desired anesthetic concentration rapidly.<br><br>4. **Calibration and Accuracy:** Desflurane vaporizers are calibrated specifically for desflurane delivery and require regular maintenance and calibration to ensure accuracy and reliability. Proper calibration of the vaporizer is essential for delivering the intended concentration of desflurane and preventing over- or under-dosing of the anesthetic agent.<br><br>5. **Safety Features:** Desflurane vaporizers incorporate safety features to prevent the delivery of excessive concentrations of desflurane and to minimize the risk of anesthesia-related complications. These may include pressure relief valves, temperature sensors, and interlocks to prevent accidental misuse or malfunction.<br><br>6. **Compatibility:** Desflurane vaporizers are designed to be compatible with anesthesia machines and delivery systems commonly used in clinical practice. They can be integrated into anesthesia workstations and anesthesia delivery systems to provide seamless delivery of desflurane during general anesthesia.<br>Overall, desflurane vaporizers are specifically engineered to deliver desflurane safely, accurately, and efficiently during the maintenance phase of general anesthesia. Understanding the unique characteristics and differences associated with desflurane | | |

| | | | |
|---|---|---|---|
| | vaporizers is essential for anesthesia providers to ensure optimal patient care and safety during surgical procedures. | | |
| 12. LA in regional | **Tell me about the differences in nerve block when using bupivacaine vs ropivacaine**<br>**Response:** Bupivacaine and ropivacaine are both long-acting local anesthetics commonly used for regional nerve blocks to provide analgesia during and after surgery. While they belong to the same class of medications, there are differences in their pharmacological properties and clinical characteristics. Here are some key differences between bupivacaine and ropivacaine in the context of nerve blocks:<br><br>1. **Duration of Action:** Bupivacaine typically has a longer duration of action compared to ropivacaine. It provides prolonged analgesia, making it suitable for single-injection nerve blocks and continuous infusion techniques. The extended duration of action of bupivacaine may be advantageous for postoperative pain management, especially in cases requiring prolonged analgesia.<br><br>2. **Onset of Action:** Ropivacaine generally has a faster onset of action compared to bupivacaine. It achieves effective nerve blockade more quickly, which may be beneficial in situations where rapid pain relief is desired, such as during emergency procedures or for patients with acute pain.<br><br>3. **Cardiac Toxicity:** Bupivacaine has a greater potential for cardiotoxicity compared to ropivacaine. It has a higher affinity for cardiac sodium channels, which can lead to systemic toxicity, including cardiac arrhythmias and cardiovascular collapse, particularly with accidental intravascular injection or systemic absorption. Ropivacaine, in contrast, exhibits less cardiotoxicity due to its lower affinity for cardiac sodium channels.<br><br>4. **Motor Blockade:** Both bupivacaine and ropivacaine can produce motor blockade, resulting in temporary paralysis of skeletal muscles. However, ropivacaine is known to produce less motor blockade compared to bupivacaine at equipotent doses. This may be advantageous in nerve blocks where preservation of motor function is desirable, such as in upper and lower extremity blocks for ambulatory surgery. | 6 | 6 |

| | | | |
|---|---|---|---|
| | 5. **Toxicity Profile:** Ropivacaine is often perceived to have a safer toxicity profile compared to bupivacaine, particularly regarding central nervous system (CNS) and cardiac toxicity. While both drugs can cause systemic toxicity if inadvertently administered intravascularly or absorbed systemically, ropivacaine's lower cardiotoxicity and reduced potential for CNS toxicity make it a preferred choice in certain clinical scenarios.<br><br>6. **Clinical Use:** The choice between bupivacaine and ropivacaine for nerve blocks depends on various factors, including the duration and onset of action required, the desired level of sensory and motor blockade, the patient's comorbidities, and the clinician's preference and experience. Both medications are widely used in clinical practice for a variety of regional anesthesia techniques, including peripheral nerve blocks, epidural anesthesia, and spinal anesthesia.<br><br>Overall, while bupivacaine and ropivacaine share many similarities as local anesthetics, they exhibit differences in their pharmacological properties, toxicity profiles, and clinical characteristics. Understanding these differences is essential for anesthesia providers to make informed decisions regarding the selection and administration of these medications for nerve blocks and other regional anesthesia techniques. | | |
| 13. Opioid metabolism | **Tell me about how opioids are eliminated in the body**<br>**Response:** Opioids are a class of medications commonly used for pain management due to their potent analgesic properties. The elimination of opioids from the body involves several processes, including metabolism, distribution, and excretion. Here's an overview of how opioids are eliminated from the body:<br><br>1. **Metabolism:** Many opioids undergo hepatic metabolism, primarily through the cytochrome P450 (CYP) enzyme system in the liver. The metabolism of opioids can result in the formation of active metabolites, which may contribute to their pharmacological effects. The specific metabolic pathways and rates of metabolism vary depending on the individual opioid and its chemical structure.<br><br>2. **First-Pass Effect:** After oral administration, opioids are absorbed from the gastrointestinal tract and transported to the liver via the portal vein. This route of | 6 | 6 |

| | | | |
|---|---|---|---|
| | administration can result in a significant portion of the drug being metabolized in the liver before reaching systemic circulation, a process known as the first-pass effect.<br><br>3. **Renal Excretion:** Once metabolized, opioid metabolites and unchanged drug molecules are excreted from the body primarily via the kidneys. Renal excretion plays a significant role in the elimination of opioids, particularly for hydrophilic compounds and their metabolites. Urinary excretion accounts for a variable but significant portion of the total elimination of opioids.<br><br>4. **Biliary Excretion:** Some opioids and their metabolites are excreted into the bile and eliminated from the body via feces. Biliary excretion represents an alternative route of elimination for opioids that are not readily eliminated via renal excretion.<br><br>5. **Half-Life:** The elimination half-life of opioids varies widely depending on factors such as the specific drug, dose, route of administration, and individual patient characteristics. The half-life of opioids ranges from a few hours to several days, reflecting differences in metabolism, distribution, and elimination kinetics.<br><br>6. **Drug Interactions:** Drug interactions can affect the metabolism and elimination of opioids, leading to alterations in their pharmacokinetic properties. Concurrent use of medications that induce or inhibit hepatic enzymes involved in opioid metabolism (e.g., CYP3A4 inducers or inhibitors) can affect the clearance and elimination of opioids from the body.<br><br>Overall, the elimination of opioids from the body involves a combination of hepatic metabolism, renal excretion, and, to a lesser extent, biliary excretion. Understanding the pharmacokinetics and elimination pathways of opioids is important for optimizing their therapeutic use and minimizing the risk of adverse effects and drug interactions. | | |
| 14. Calculation of blood volume | **What are different methods to calculate blood volume in my 70 kilogram male patient?**<br>**Response**: There are several methods to estimate blood volume in a patient, with varying degrees of accuracy and complexity. Here are some common methods:<br>1. **Weight-Based Estimation**: One simple method is to use a formula based on the patient's weight. One commonly used formula is the Nadler's formula:<br>- Blood volume (BV) = k1 * weight (kg) + k2 * height (cm) + k3 | 6 | 6 |

| | | | |
|---|---|---|---|
| | - Where k1, k2, and k3 are constants based on gender. For males, typical values are k1 = 0.3669, k2 = 0.03219, and k3 = 0.6041.<br>2. **Hematocrit (Hct) Method**: This method uses the patient's hematocrit level, which is the proportion of blood volume that is occupied by red blood cells. The formula is:<br>- BV = [Total blood volume (TBV)] / [(1 - Hct)]<br>- TBV can be calculated using the formula: TBV (in mL) = 70 mL/kg * body weight (in kg) for males.<br>3. **Indicator Dilution Techniques**: These involve injecting a known volume of indicator substance into the bloodstream and measuring its concentration over time. From the dilution curve, blood volume can be calculated.<br>4. **Radioisotope Dilution**: This method involves injecting a known amount of a radioisotope (e.g., iodine-125-labeled albumin) into the bloodstream and measuring its distribution in the body to calculate blood volume.<br>5. **Dye Dilution Technique**: Similar to radioisotope dilution, this method uses a dye (e.g., indocyanine green) instead of a radioactive substance.<br>6. **Direct Blood Volume Measurement**: This method involves withdrawing a known volume of blood from the patient and measuring its volume directly.<br>Each method has its advantages and limitations, and the choice of method may depend on factors such as the patient's condition, available resources, and the level of accuracy required. It's essential to consider these factors when selecting the most appropriate method for your patient. | | |
| 15. Etomidate side effects | **What are the side effects of etomidate?**<br>**Response**: Etomidate is an intravenous anesthetic agent commonly used for induction of anesthesia due to its rapid onset of action and minimal cardiovascular and respiratory depression. While etomidate is generally well-tolerated, it can produce a range of side effects, including:<br><br>1. **Pain on Injection:** Etomidate can cause pain or discomfort at the injection site, particularly when administered intravenously. This is often mitigated by pretreatment with lidocaine or by administering the drug more slowly.<br><br>2. **Myoclonus:** Myoclonus, or involuntary muscle contractions or twitches, can occur shortly after administration of etomidate. This side effect is typically transient and resolves spontaneously but may be alarming to patients and clinicians. | 6 | 6 |

| | | | |
|---|---|---|---|
| | 3. **Adrenal Suppression:** Etomidate suppresses adrenal cortical function by inhibiting steroidogenesis, particularly cortisol synthesis. This effect is relatively short-lived but can be significant enough to warrant caution in patients with adrenal insufficiency or those requiring prolonged sedation.<br><br>4. **Nausea and Vomiting:** Nausea and vomiting may occur following administration of etomidate, although these side effects are less common compared to some other anesthetic agents.<br><br>5. **Transient Hypotension:** Etomidate can cause transient hypotension (low blood pressure) due to its vasodilatory effects. This effect is typically mild and short-lived but may be more pronounced in patients with pre-existing hypovolemia or compromised cardiovascular function.<br><br>6. **Respiratory Depression:** While etomidate is known for its relative respiratory stability compared to other induction agents, it can still cause respiratory depression, particularly when administered rapidly or in high doses. Careful monitoring of respiratory function is essential during etomidate administration.<br><br>7. **Postoperative Nausea and Vomiting (PONV):** Etomidate may contribute to the development of postoperative nausea and vomiting, although the risk is generally lower compared to some other anesthetic agents.<br><br>8. **Intravenous Thrombophlebitis:** Administration of etomidate via peripheral venous access can occasionally cause thrombophlebitis, characterized by inflammation and clot formation at the injection site.<br><br>It's important for healthcare providers to be aware of these potential side effects of etomidate and to monitor patients closely during and after administration to ensure their safety and well-being. Individual patient factors, including age, comorbidities, and concurrent medications, should also be considered when assessing the risk of adverse effects associated with etomidate administration. | | |
| 16. Periop anaphylaxis | **How can an anesthesiologist recognize perioperative anaphylaxis?**<br>**Response**: Recognizing perioperative anaphylaxis is crucial for an anesthesiologist to ensure prompt management and patient safety. Here are some key steps an anesthesiologist can take to recognize perioperative anaphylaxis: | 6 | 6 |

| | | | |
|---|---|---|---|
| | 1. **Clinical Suspicion**: Anesthesiologists should maintain a high index of suspicion for perioperative anaphylaxis, especially in patients with a history of allergies or previous adverse reactions to anesthesia or other medications.<br>2. **Immediate Signs and Symptoms**: Anaphylaxis typically presents rapidly and can manifest with a variety of symptoms, including:<br>- Skin reactions such as flushing, urticaria (hives), or angioedema (swelling)<br>- Respiratory symptoms like wheezing, dyspnea (difficulty breathing), or stridor<br>- Cardiovascular symptoms such as hypotension (low blood pressure), tachycardia (rapid heart rate), or cardiac arrest<br>- Gastrointestinal symptoms like nausea, vomiting, or abdominal pain<br>3. **Vital Signs Monitoring**: Continuous monitoring of vital signs, including blood pressure, heart rate, respiratory rate, and oxygen saturation, is essential for detecting any sudden changes indicative of anaphylaxis.<br>4. **Diagnostic Tests**: Laboratory tests such as serum tryptase levels and specific IgE testing may aid in confirming the diagnosis of anaphylaxis. However, these tests are not immediately available and should not delay the initiation of treatment.<br>5. **Review of Medications and Allergies**: Anesthesiologists should review the patient's medication history, including any known allergies or previous adverse reactions, to identify potential triggers for anaphylaxis.<br>6. **Communication with the Surgical Team**: Prompt communication with the surgical team is essential to coordinate the management of perioperative anaphylaxis effectively. This includes informing the surgeon and other healthcare providers about the suspected diagnosis and initiating appropriate treatment measures.<br>7. **Treatment**: Immediate treatment of anaphylaxis involves:<br>- Discontinuation of the suspected triggering agent (e.g., anesthesia drugs, antibiotics)<br>- Administration of intramuscular epinephrine as the first-line treatment for severe anaphylaxis<br>- Airway management and oxygen supplementation as needed<br>- Intravenous fluids to support blood pressure<br>- Additional medications such as antihistamines and corticosteroids may be administered to mitigate allergic reactions.<br>8. **Documentation**: Accurate documentation of the events, including the timing of symptom onset, interventions performed, and response to treatment, is essential for continuity of care and medicolegal purposes. | | |

| | | | |
|---|---|---|---|
| | By remaining vigilant, promptly recognizing the signs and symptoms of perioperative anaphylaxis, and initiating appropriate treatment, anesthesiologists can help mitigate the potentially life-threatening consequences of this serious allergic reaction. | | |
| 17. Succinylcholine-induced bradycardia | **Tell me what types of patients are at risk for succinylcholine induced bradycardia**<br>**Response**: Succinylcholine-induced bradycardia typically occurs in patients who are sensitive to the effects of the drug or have certain underlying medical conditions. Here are some factors that may increase the risk of succinylcholine-induced bradycardia:<br>**Children**: Pediatric patients, especially infants and neonates, are more prone to bradycardia after succinylcholine administration.<br>**Elderly Patients**: Older adults may experience bradycardia due to age-related changes in autonomic function and decreased cardiac reserve.<br>**Patients with Autonomic Dysfunction**: Individuals with autonomic dysfunction, such as those with autonomic neuropathy, may be at increased risk due to impaired sympathetic response.<br>**Patients with Cardiac Disease**: Those with pre-existing cardiac conditions, such as ischemic heart disease, heart failure, or conduction abnormalities, are more susceptible to succinylcholine-induced bradycardia.<br>**Electrolyte Imbalance**: Patients with electrolyte abnormalities, particularly hyperkalemia, are at higher risk because succinylcholine can cause potassium release from cells, exacerbating the hyperkalemic state and leading to bradycardia.<br>**Denervating Conditions**: Patients with denervating conditions like spinal cord injury or neuromuscular disorders (e.g., Guillain-Barré syndrome) may exhibit exaggerated responses to succinylcholine due to altered neuromuscular transmission.<br>**Medication Interactions**: Concurrent use of medications that affect cardiac conduction or autonomic function, such as beta-blockers or calcium channel blockers, can potentiate succinylcholine-induced bradycardia.<br>**Rare Genetic Conditions**: Certain rare genetic conditions affecting cholinesterase activity or neuromuscular transmission may predispose individuals to adverse reactions to succinylcholine, including bradycardia.<br>**Pregnant Women**: Pregnant patients may have altered responses to succinylcholine due to physiological changes in pregnancy, including increased sensitivity to neuromuscular blocking agents and changes in cardiac function.<br>**Obese Patients**: Obesity can impact drug distribution and metabolism, potentially altering the response to succinylcholine and increasing the risk of bradycardia. | 6 | 6 |

| | | | |
|---|---|---|---|
| | It's crucial for healthcare providers to assess each patient's risk factors before administering succinylcholine and to be prepared to manage bradycardia promptly if it occurs. | | |
| 18. PONV: treatment | **Tell me how I can treat my patient with postoperative nausea and vomiting** **Response**: Treating postoperative nausea and vomiting (PONV) involves a combination of preventive measures and pharmacological interventions. Here's a general approach:<br>1. **Preventive Measures**:<br>- Preoperative Assessment: Identify patients at high risk for PONV based on factors such as history of motion sickness, non-smoking status, female gender, and type of surgery.<br>- Avoidance of Triggers: Minimize intraoperative factors that can trigger PONV, such as volatile anesthetics and opioids.<br>- Regional Anesthesia: Consider regional anesthesia techniques as they may reduce the need for general anesthesia and decrease the risk of PONV.<br>- Antiemetic Prophylaxis: Administer prophylactic antiemetics before induction of anesthesia. Common choices include serotonin (5-HT3) receptor antagonists, dexamethasone, and/or droperidol.<br>2. **Pharmacological Treatment**:<br>- First-line Treatment: If PONV occurs despite prophylaxis, administer antiemetics. Common choices include:<br>- Serotonin (5-HT3) Receptor Antagonists: Ondansetron, granisetron, or palonosetron.<br>- Dopamine Antagonists: Metoclopramide or droperidol (with caution due to potential QT prolongation).<br>- Corticosteroids: Dexamethasone may be used alone or in combination with other antiemetics.<br>- Rescue Therapy: If initial treatment fails, consider alternative or combination therapy. Options include:<br>- NK1 Receptor Antagonists: Aprepitant or fosaprepitant, often used in combination with a 5-HT3 receptor antagonist and corticosteroid.<br>- Phenothiazines: Promethazine or prochlorperazine may be used if other options fail.<br>- Cannabinoids: Synthetic cannabinoids like dronabinol or nabilone can be considered in refractory cases.<br>3. **Non-pharmacological Measures**: | 6 | 6 |

| | | | |
|---|---|---|---|
| | - Positioning: Keep the patient's head elevated to reduce the risk of aspiration.<br>- Hydration: Administer intravenous fluids to prevent dehydration.<br>- Nutritional Support: Provide clear liquids and bland foods once the patient can tolerate oral intake.<br>4. **Monitoring and Follow-up**:<br>- Monitor the patient for signs of dehydration, electrolyte imbalance, or aspiration.<br>- Assess the effectiveness of antiemetic therapy and adjust treatment as needed.<br>- Provide reassurance and support to alleviate anxiety, which can exacerbate nausea and vomiting.<br>5. **Consider Alternative Therapies**:<br>- Acupressure wristbands and aromatherapy with ginger or peppermint may offer some relief for mild cases or as adjunctive therapy.<br>Always tailor the treatment approach to the individual patient's needs and consider any comorbid conditions or contraindications to specific medications. If PONV persists despite appropriate interventions, consultation with a specialist in anesthesia or gastroenterology may be warranted. | | |
| 19. Beta blocker, periop indications | **Tell me the perioperative plan of beta blocker therapy in my patient who has a systolic blood pressure of 200 mmHg during surgery**<br>**Response**: In a patient with systolic blood pressure of 200 mmHg during surgery, the perioperative management of beta blocker therapy would typically involve careful consideration of various factors to optimize blood pressure control and minimize risks. Here's a general outline of the perioperative plan:<br>1. **Assessment and Risk Stratification**: Evaluate the patient's medical history, current medications, and the nature of the surgery to assess cardiovascular risk factors. Determine if the elevated blood pressure is due to anesthesia induction, surgical stress, pain, or other causes.<br>2. **Immediate Management**: If the elevated blood pressure is severe and potentially harmful, immediate interventions may be necessary to reduce it. This could include short-acting antihypertensive medications like nitroprusside or nicardipine administered intravenously.<br>3. **Beta Blocker Therapy Consideration**: If the patient is already on beta blocker therapy preoperatively, the decision to continue or withhold beta blockers perioperatively depends on various factors: | 5 | 6 |

| | | | |
|---|---|---|---|
| | - **Indications for Beta Blockers**: If the patient has a history of coronary artery disease, heart failure, or significant cardiac risk factors, beta blockers may be beneficial for perioperative cardiac protection.<br>- **Hemodynamic Stability**: Consider the patient's overall hemodynamic stability. If there are signs of decompensation or shock, beta blockers may be withheld or used cautiously.<br>- **Type of Beta Blocker**: Selective beta-1 blockers like metoprolol are preferred over non-selective beta blockers like propranolol, especially in patients with respiratory comorbidities.<br>- **Dosing and Timing**: Adjust the dose and timing of beta blocker administration based on the patient's preoperative regimen and the anticipated stress response to surgery.<br>4. **Intraoperative Monitoring**: Continuous monitoring of blood pressure, heart rate, and other vital signs is essential during surgery. Anesthesia depth, fluid balance, and other factors should be optimized to maintain hemodynamic stability.<br>5. **Postoperative Care**: After surgery, continue monitoring the patient closely in the recovery area or intensive care unit. Adjust medications as needed to maintain blood pressure within a safe range while minimizing the risk of adverse events such as hypotension or bradycardia.<br>6. **Gradual Titration**: If beta blockers were temporarily withheld perioperatively, consider reintroducing them gradually once the patient's hemodynamic status stabilizes. Titrate the dose carefully to achieve optimal blood pressure control without compromising cardiac output or perfusion.<br>7. **Communication and Collaboration**: Ensure clear communication between the surgical team, anesthesiologist, and other healthcare providers involved in the patient's care. Collaboration is essential to coordinate perioperative management and address any complications promptly.<br>8. **Follow-up**: Schedule appropriate follow-up appointments to monitor the patient's recovery, adjust medications as needed, and address any postoperative complications or concerns.<br>Overall, the perioperative management of beta blocker therapy in a patient with elevated blood pressure during surgery requires a balanced approach that considers the patient's cardiovascular risk profile, current clinical status, and the goals of perioperative care. Close monitoring and individualized management are key to optimizing outcomes and reducing the risk of perioperative complications. | | |

| | | | |
|---|---|---|---|
| 20. Consent in anesthesia | **How do you approach consent for regional anesthesia?**<br>**Response:** Obtaining consent for regional anesthesia is a crucial part of the preoperative process. Here's a general approach:<br>1. **Education**: Start by educating the patient about regional anesthesia, explaining what it is, how it works, and its potential benefits and risks compared to other forms of anesthesia.<br>2. **Risks and Benefits Discussion**: Discuss the specific risks and benefits of the chosen regional anesthesia technique for the patient's procedure. This should include potential complications such as nerve damage, infection, bleeding, allergic reactions, and failure of the block.<br>3. **Alternative Options**: Inform the patient about alternative anesthesia options available for their procedure, such as general anesthesia or local anesthesia, and discuss the pros and cons of each.<br>4. **Informed Consent Form**: Provide the patient with an informed consent form detailing the discussion points, risks, benefits, and alternatives. Ensure the patient understands the information on the form and address any questions or concerns they may have before they sign it.<br>5. **Documentation**: Document the consent process in the patient's medical record, including the specific type of regional anesthesia discussed, the risks and benefits explained, any alternative options considered, and the patient's agreement or refusal.<br>6. **Confirmation**: Before the procedure, confirm the patient's understanding and consent for the regional anesthesia technique again. This provides an opportunity to address any last-minute questions or concerns.<br>7. **Continuous Communication**: Throughout the procedure, maintain open communication with the patient, ensuring they remain comfortable and informed about what is happening.<br><br>Remember, the consent process should be tailored to each individual patient, taking into account their medical history, preferences, and level of understanding. It's essential to ensure that the patient is fully informed and actively participates in the decision-making process regarding their anesthesia care. | 6 | 6 |
| 21. Anesthesia machine | **In the event of an anesthesia machine failure, what are the next steps to ensure proper care is provided to a patient undergoing general anesthesia?**<br>**Response:** In the event of an anesthesia machine failure during a procedure, the priority is to ensure the safety and well-being of the patient. Here are the general steps that healthcare providers typically follow: | 5 | 5 |

| | | | |
|---|---|---|---|
| | 1. **Maintain Calmness**: It's crucial for the medical team to stay calm and composed to effectively address the situation.<br>2. **Assess the Patient**: Quickly assess the patient's condition, including their vital signs, level of consciousness, and oxygenation status.<br>3. **Provide Ventilation**: If the anesthesia machine fails, the patient may stop receiving oxygen and anesthesia. Begin manual ventilation using a bag-valve-mask (BVM) device to deliver oxygen to the patient's lungs.<br>4. **Check Backup Equipment**: Many anesthesia workstations have backup systems or manual ventilation devices that can be used in the event of a primary machine failure. Verify if any backup equipment is available and functional.<br>5. **Notify the Anesthesiologist**: Inform the attending anesthesiologist immediately about the equipment failure so they can provide guidance and assistance.<br>6. **Activate Emergency Response**: If necessary, activate the hospital's emergency response system to summon additional help, such as anesthesia technicians or additional medical personnel.<br>7. **Monitor Patient Closely**: Continuously monitor the patient's vital signs, including oxygen saturation, heart rate, blood pressure, and respiratory rate, to ensure they remain stable.<br>8. **Consider Alternative Anesthesia Techniques**: Depending on the situation and the stage of the procedure, the medical team may need to consider alternative anesthesia techniques or even abort the procedure if the patient's safety is at risk.<br>9. **Prepare for Transfer**: If the patient's condition cannot be stabilized or if the procedure cannot be safely completed in the current environment, prepare for transfer to a location where appropriate care can be provided, such as an operating room or intensive care unit.<br>10. **Document the Incident**: It's important to document the equipment failure and the actions taken to address it in the patient's medical record for review and quality improvement purposes.<br>11. **Review and Learn**: After the incident is resolved, conduct a thorough review of the equipment failure to identify any underlying causes or system failures. This can help prevent similar incidents in the future through quality improvement initiatives and additional staff training.<br>Remember, these steps may vary depending on the specific circumstances of the equipment failure and the resources available in each clinical setting. Prioritizing patient safety and effective communication among the medical team are essential throughout the process. | | |

| | | | |
|---|---|---|---|
| 22. Periop dialysis | **What are preoperative indications for dialysis?**<br>**Response:** Preoperative indications for dialysis refer to situations where it's necessary to initiate dialysis before a surgical procedure due to the patient's kidney function being compromised. These indications may include:<br>1. **Severe Electrolyte Imbalance**: Such as hyperkalemia (high potassium levels), severe acidosis, or hyperphosphatemia (high phosphate levels), which can predispose the patient to cardiac arrhythmias, muscle weakness, and other complications.<br>2. **Fluid Overload**: Patients with advanced kidney disease may have difficulty managing fluid balance, leading to edema, pulmonary congestion, and heart failure. Dialysis can help remove excess fluid.<br>3. **Uremic Symptoms**: Severe symptoms of uremia, including nausea, vomiting, lethargy, confusion, and pericarditis, may necessitate urgent dialysis to alleviate symptoms and stabilize the patient.<br>4. **Uncontrolled Hypertension**: Hypertension that is resistant to medical management may require dialysis to achieve adequate blood pressure control.<br>5. **Acute Kidney Injury (AKI)**: Patients with acute deterioration of kidney function, especially if associated with oliguria (low urine output), may require dialysis to support renal function until kidney function improves.<br>6. **Planned Major Surgery**: Some surgeries may necessitate dialysis preoperatively to optimize the patient's metabolic and fluid status, reducing the risk of perioperative complications.<br>7. **Chronic Kidney Disease (CKD) Stage 4-5**: Patients with severe chronic kidney disease may already be on maintenance dialysis or may require dialysis initiation prior to surgery due to their compromised renal function.<br>It's crucial for the medical team to assess each patient individually and consider factors such as the severity of kidney dysfunction, the urgency of the surgical procedure, and the overall health status of the patient when determining the need for preoperative dialysis. | 6 | 6 |
| 23. Expiratory valve malfunction | **How do I troubleshoot a malfunctioning expiratory valve on the anesthesia machine?**<br>**Response:** Troubleshooting a malfunctioning expiratory valve on an anesthesia machine is essential for ensuring patient safety and the delivery of adequate ventilation during anesthesia. Here's a step-by-step guide to troubleshoot a malfunctioning expiratory valve: | 6 | 6 |

| | | | |
|---|---|---|---|
| | 1. **Assess the Clinical Situation:** If you suspect a malfunctioning expiratory valve, assess the patient's ventilation status and vital signs immediately. Ensure that the patient is adequately oxygenated and ventilated. If the patient's condition is unstable, consider manually ventilating the patient with a bag-mask device while troubleshooting the anesthesia machine.<br><br>2. **Check the Expiratory Valve Position:** Ensure that the expiratory valve is in the correct position and properly seated. Verify that the valve is not stuck in the closed position, which could obstruct exhalation and cause barotrauma. If the valve is visibly stuck or obstructed, attempt to manually open it and clear any debris or obstructions.<br><br>3. **Inspect for Damage or Debris:** Thoroughly inspect the expiratory valve assembly for any signs of damage, wear, or debris accumulation. Look for cracks, tears, or misalignment of components that may affect the valve's function. Clean the valve assembly as needed to remove any accumulated dirt, dust, or foreign material.<br><br>4. **Check for Proper Functioning:** Activate the anesthesia machine's breathing circuit and observe the movement of the expiratory valve during the respiratory cycle. Ensure that the valve opens smoothly during expiration to allow the patient to exhale and closes properly during inspiration to prevent gas leakage. Listen for any abnormal sounds or resistance that may indicate a malfunction.<br><br>5. **Perform Leak Testing:** Perform a leak test to assess the integrity of the breathing circuit and expiratory valve. Close the pop-off valve and manually ventilate the patient with a bag-mask device while occluding the patient's airway. Monitor the pressure gauge for any signs of pressure buildup, which may indicate a leak in the breathing circuit or expiratory valve.<br><br>6. **Replace or Repair the Valve:** If the expiratory valve is damaged, malfunctioning, or unable to be repaired, replace it with a new valve according to the manufacturer's recommendations. Ensure that the replacement valve is compatible with the anesthesia machine and properly installed to prevent gas leaks or improper function. | | |

| | | | |
|---|---|---|---|
| | 7. **Document the Troubleshooting Process:** Document the steps taken to troubleshoot the malfunctioning expiratory valve, including any observations, interventions, and outcomes. Ensure that all documentation is accurate, thorough, and compliant with institutional protocols and regulatory requirements.<br><br>8. **Perform Function Checks:** After troubleshooting and repairing the expiratory valve, perform function checks and verification tests to ensure that the anesthesia machine is operating correctly and safely. Monitor the patient's ventilation status and vital signs continuously during anesthesia to detect any further issues or complications.<br><br>If you encounter any difficulties or uncertainties during the troubleshooting process, seek assistance from qualified personnel, such as biomedical engineers or anesthesia technicians, to ensure proper resolution of the issue and the safe delivery of anesthesia care. | | |
| 24. Scavenging system failure | **How do I check the anesthesia machine for a malfunctioned anesthesia gas scavenging system?**<br>**Response:** Checking the anesthesia gas scavenging system (AGSS) is crucial to ensure the safe removal of excess anesthetic gases and to prevent occupational exposure to anesthesia providers and other healthcare personnel. Here's a step-by-step guide to check the anesthesia machine for a malfunctioning AGSS:<br>1. **Visual Inspection:** Begin by visually inspecting the AGSS components, including the scavenging interface, tubing, canister, and vacuum regulator. Ensure that all components are intact, properly connected, and free from damage, kinks, or obstructions. Verify that the scavenging interface is securely attached to the patient circuit and that the connections are airtight.<br>2. **Check Vacuum Source:** Verify that the vacuum source (e.g., central vacuum system or standalone vacuum pump) supplying suction to the AGSS is functioning properly. Check the vacuum gauge or pressure indicator to ensure that the vacuum level is within the recommended range for scavenging anesthesia gases.<br>3. **Perform Leak Test:** Perform a leak test to assess the integrity of the AGSS and identify any leaks or defects. Close the pop-off valve and occlude the patient end of the breathing circuit. Activate the anesthesia machine and observe the vacuum gauge for any fluctuations or deviations from baseline, which may indicate a leak in the scavenging system. | 6 | 6 |

| | | | |
|---|---|---|---|
| | 4. **Check Negative Pressure Relief Valve:** Ensure that the negative pressure relief valve (NPRV) is functioning correctly to prevent excessive negative pressure in the breathing circuit and patient interface. Test the NPRV by occluding the scavenging interface and verifying that it opens at the specified pressure to release excess vacuum.<br>5. **Test Suction:** Test the suction function of the AGSS by attaching a test lung or suction bulb to the scavenging interface. Activate the vacuum source and observe the suction device for proper function and adequate suction force. Verify that the scavenging system effectively removes gas from the test lung or suction bulb.<br>6. **Perform Functional Check:** Activate the anesthesia machine and deliver a test dose of anesthesia gas while monitoring the scavenging system's performance. Observe the scavenging interface for proper evacuation of exhaled gases and verify that there are no audible leaks or abnormal sounds during operation.<br>7. **Document Findings:** Document the results of the AGSS check, including any observations, interventions, and outcomes. Ensure that all documentation is accurate, thorough, and compliant with institutional protocols and regulatory requirements.<br>8. **Routine Maintenance:** Schedule routine maintenance and inspection of the AGSS according to the manufacturer's recommendations and institutional policies. Regularly monitor the scavenging system for signs of wear, damage, or malfunction, and promptly address any issues to ensure continuous safe operation.<br>If you encounter any difficulties or uncertainties during the AGSS check, seek assistance from qualified personnel, such as biomedical engineers or anesthesia technicians, to ensure proper resolution of the issue and the safe use of the anesthesia machine. | | |
| 25. LAST | **Tell me the early to late symptoms seen with local anesthetic systemic toxicity**<br>**Response:** Local anesthetic systemic toxicity (LAST) occurs when local anesthetic agents are inadvertently administered systemically, leading to toxic effects on various organ systems. The symptoms of LAST can range from mild to severe and may progress rapidly without prompt recognition and intervention. Here are the early and late symptoms seen with LAST:<br>**Early Symptoms:**<br>1. **Neurological Symptoms:** The earliest signs of LAST often involve the central nervous system (CNS) and may include symptoms such as:<br>- Perioral numbness or tingling<br>- Metallic taste in the mouth<br>- Dizziness or lightheadedness | 6 | 6 |

| | | | |
|---|---|---|---|
| | - Blurred vision or visual disturbances<br>- Tinnitus (ringing in the ears)<br>- Restlessness or agitation<br>- Anxiety or apprehension<br>- Confusion or disorientation<br><br>2. **Cardiovascular Symptoms:** As LAST progresses, cardiovascular symptoms may develop, reflecting the systemic absorption and distribution of the local anesthetic agent. These symptoms may include:<br>- Hypotension (low blood pressure)<br>- Tachycardia (rapid heart rate)<br>- Dysrhythmias (arrhythmias), such as ventricular ectopy or ventricular tachycardia<br>- Cardiac arrest or cardiovascular collapse<br><br>**Late Symptoms:**<br><br>1. **Severe CNS Depression:** In severe cases of LAST, profound CNS depression may occur, leading to loss of consciousness, seizures, coma, or respiratory arrest. These late neurological symptoms are indicative of severe systemic toxicity and require immediate intervention to prevent further complications.<br>2. **Cardiorespiratory Collapse:** Without prompt intervention, LAST can progress to cardiovascular collapse, manifested by profound hypotension, bradycardia, cardiac arrest, and respiratory failure. Cardiorespiratory collapse represents a life-threatening emergency and requires immediate resuscitative measures, including advanced cardiac life support (ACLS) interventions and administration of lipid emulsion therapy.<br><br>It's important to note that the onset and progression of LAST can vary depending on factors such as the dose and potency of the local anesthetic agent, the route of administration, the patient's age and health status, and the presence of coexisting medical conditions. Early recognition of the signs and symptoms of LAST, along with prompt initiation of appropriate treatment, is crucial for optimizing patient outcomes and minimizing the risk of complications associated with systemic local anesthetic toxicity. | | |
| 26. Tachycardia treatment | **Tell me about the algorithm used to treat the different types of tachycardia**<br>**Response:** Treating tachycardia involves identifying the underlying cause and determining the appropriate intervention based on the patient's clinical presentation | 6 | 3 |

| | | | |
|---|---|---|---|
| | and hemodynamic stability. Various algorithms and guidelines, such as those provided by the American Heart Association (AHA) and the European Society of Cardiology (ESC), outline systematic approaches to managing different types of tachycardia. Here's a simplified algorithm for treating different types of tachycardia:<br><br>1. **Assess Patient's Hemodynamic Status:**<br>- Determine if the patient is stable or unstable based on clinical signs such as blood pressure, mental status, and signs of shock.<br>- In unstable patients with signs of shock or hemodynamic compromise, proceed directly to synchronized cardioversion without delay.<br><br>2. **Identify the Type of Tachycardia:**<br>- Determine whether the tachycardia is narrow complex (QRS duration $< 0.12$ seconds) or wide complex (QRS duration $\geq 0.12$ seconds) on the electrocardiogram (ECG).<br><br>3. **Treatment of Narrow Complex Tachycardia:**<br>- For stable narrow complex tachycardia, assess for regularity and the presence of P waves:<br>- If regular and monomorphic:<br>- Consider vagal maneuvers (e.g., Valsalva maneuver, carotid sinus massage).<br>- Administer adenosine as a first-line pharmacological therapy (6 mg rapid IV push followed by a 20 mL saline flush).<br>- If adenosine is ineffective, consider beta-blockers, calcium channel blockers, or antiarrhythmic medications (e.g., verapamil, diltiazem).<br>- If irregular or polymorphic:<br>- Suspect atrial fibrillation or atrial flutter with variable conduction.<br>- Manage based on the patient's hemodynamic stability and the presence of underlying structural heart disease.<br><br>4. **Treatment of Wide Complex Tachycardia:**<br>- For stable wide complex tachycardia: Assess for regularity and the presence of atrial activity.<br>- If regular and monomorphic: Consider adenosine as a diagnostic and therapeutic trial (for suspected supraventricular tachycardia with aberrant conduction). Administer amiodarone, procainamide, or lidocaine for sustained ventricular tachycardia. | | |

| | | | |
|---|---|---|---|
| | - If irregular or polymorphic: Suspect ventricular tachycardia or polymorphic ventricular tachycardia (e.g., Torsades de Pointes). Administer amiodarone or lidocaine for stable ventricular tachycardia.<br>- For unstable wide complex tachycardia: Proceed to synchronized cardioversion without delay.<br><br>5. **Monitor Response to Treatment:**<br>- Continuously monitor the patient's ECG, blood pressure, and clinical status throughout treatment.<br>- Adjust therapy based on the patient's response and hemodynamic stability.<br><br>6. **Consider Expert Consultation:**<br>- In cases of diagnostic uncertainty or refractory tachycardia, consider consulting a cardiologist or electrophysiologist for further evaluation and management.<br><br>7. **Address Underlying Causes:**<br>- Identify and address any reversible causes of tachycardia, such as electrolyte abnormalities, ischemia, or drug toxicity.<br><br>This algorithm provides a general framework for the management of different types of tachycardia but should be adapted based on individual patient factors, institutional protocols, and the availability of resources. Always refer to current guidelines and consult with a specialist when appropriate for complex or challenging cases. | | |
| 27. Ventilation assessment | **How to assess a patient's ventilation status during positive pressure ventilation?**<br>**Response:** Assessing a patient's ventilation status during positive pressure ventilation (PPV) is crucial to ensure adequate gas exchange and prevent complications such as hypoventilation, hypoxemia, and barotrauma. Here's a step-by-step guide to assessing ventilation status during PPV:<br><br>1. **Monitor Oxygenation:**<br>- Continuously monitor the patient's oxygen saturation (SpO2) using pulse oximetry. A normal SpO2 value is typically above 95% in most patients.<br>- Observe for signs of hypoxemia, such as cyanosis, decreased SpO2, or changes in the patient's level of consciousness. | 6 | 6 |

| | | | |
|---|---|---|---|
| | 2. **Assess Airway Patency:**<br>- Confirm the patency of the patient's airway by observing chest rise and fall during ventilation. Ensure that the endotracheal tube (ETT) or supraglottic airway device (e.g., laryngeal mask airway) is properly positioned and not obstructed.<br>- Auscultate breath sounds bilaterally to assess for equal and symmetric breath sounds, which indicate adequate ventilation and lung inflation.<br><br>3. **Monitor Ventilation Parameters:**<br>- Evaluate the tidal volume delivered during each breath. Monitor the ventilator settings and observe the chest wall movement to ensure adequate tidal volume delivery and lung expansion.<br>- Assess the respiratory rate (RR) to ensure an appropriate rate of ventilation. Normal RR varies depending on age, with typical values ranging from 12 to 20 breaths per minute in adults.<br>- Verify the peak inspiratory pressure (PIP) and plateau pressure (Pplat) on the ventilator display to monitor for signs of airway obstruction, increased resistance, or decreased compliance.<br><br>4. **Assess End-Tidal CO2 (EtCO2):**<br>- Monitor the patient's EtCO2 using capnography to assess the adequacy of ventilation and confirm proper ETT placement.<br>- A normal EtCO2 waveform should display a consistent baseline with regular fluctuations corresponding to each breath. EtCO2 values typically range from 35 to 45 mmHg in mechanically ventilated patients.<br><br>5. **Evaluate Chest Movement and Compliance:**<br>- Assess the chest wall movement and chest excursion during ventilation. Observe for symmetrical chest rise and fall, which indicate effective ventilation and lung compliance.<br>- Monitor for signs of decreased lung compliance (e.g., decreased chest excursion, increased PIP, or Pplat) or increased airway resistance, which may require adjustments to ventilator settings or interventions such as bronchodilator therapy.<br><br>6. **Clinical Assessment:**<br>- Evaluate the patient's clinical response to ventilation, including changes in respiratory effort, oxygenation, and hemodynamic stability. | | |

| | | | |
|---|---|---|---|
| | - Assess for signs of respiratory distress or fatigue, such as increased work of breathing, use of accessory muscles, or paradoxical chest movement.<br><br>7. **Continuous Monitoring and Reassessment:**<br>- Continuously monitor the patient's ventilation status throughout the procedure or treatment.<br>- Reassess ventilation parameters regularly and adjust ventilator settings as needed based on the patient's response and clinical condition.<br><br>Effective assessment of ventilation status during PPV requires a combination of clinical observation, monitoring of ventilator parameters, and interpretation of physiological data. Prompt recognition of ventilation abnormalities allows for timely intervention and optimization of respiratory support to maintain adequate gas exchange and optimize patient outcomes. | | |
| 28. ASA fasting guideline | **Tell me how I can counsel my patient on American Society for Anesthesiologists fasting guidelines**<br>**Response**: Title: Counseling your patient on the American Society for Anesthesiologists (ASA) fasting guidelines is crucial for ensuring their safety and comfort before undergoing anesthesia or sedation. Here's a general guide you can follow:<br>1. **Educate**: Start by explaining the purpose of fasting before anesthesia. Emphasize that it's to reduce the risk of aspiration (inhaling stomach contents) during the procedure, which can lead to serious complications such as pneumonia.<br>2. **Review Guidelines**: Familiarize yourself with the ASA fasting guidelines, which typically recommend:<br>- Clear liquids: Allowed up to 2 hours before the procedure.<br>- Light meals: Allowed up to 6 hours before the procedure.<br>- Heavy meals: Allowed up to 8 hours before the procedure.<br>3. **Individual Considerations**: Discuss any specific medical conditions or medications your patient is taking that might affect their fasting requirements. For example, patients with diabetes may need special instructions.<br>4. **Hydration**: Emphasize the importance of staying hydrated before fasting begins. Clear liquids like water, apple juice, and clear broths are usually allowed up to 2 hours before the procedure. | 6 | 6 |

| | | | |
|---|---|---|---|
| | 5. **Medication Guidance**: Provide clear instructions about taking essential medications on the day of the procedure, especially if they need to be taken with water.<br>6. **Avoidance of Certain Substances**: Advise your patient to avoid alcohol and smoking before the procedure, as these can affect anesthesia and recovery.<br>7. **Follow-Up**: Encourage your patient to contact you if they have any questions or concerns about fasting before the procedure.<br>8. **Documentation**: Make sure to document your discussion about fasting guidelines in the patient's medical record for future reference.<br>9. **Preoperative Visit**: If possible, schedule a preoperative visit to review fasting instructions again and address any last-minute concerns.<br>10. **Post-Procedure Guidance**: After the procedure, remind your patient to start with clear liquids and progress to solid foods as tolerated, following the guidance of their healthcare provider.<br>By following these steps, you can help ensure that your patient adheres to the ASA fasting guidelines, minimizing the risk of complications during their procedure. | | |
| 29. Predictor of difficult laryngoscopy | **What are predictors of difficult laryngoscopy?**<br>**Response**: SevSeveral factors can predict difficult laryngoscopy, making intubation challenging. These predictors include:<br>1. **Mallampati Score**: This is assessed by examining the visibility of the soft palate, uvula, tonsillar pillars, and base of the uvula. A higher Mallampati score (III or IV) indicates a higher likelihood of difficult laryngoscopy.<br>2. **Thyromental Distance**: A shorter thyromental distance (less than 6 cm) can make it difficult to visualize the larynx.<br>3. **Neck Mobility**: Limited neck mobility, as seen in patients with cervical spine pathology or obesity, can impede proper positioning for laryngoscopy.<br>4. **Mandibular Protrusion**: Patients with a small mandible or limited jaw protrusion may present challenges during laryngoscopy.<br>5. **Interincisor Gap**: An interincisor gap of less than 3 cm may indicate a difficult laryngoscopy.<br>6. **Obesity**: Patients with a higher body mass index (BMI) may have excessive soft tissue in the oropharynx, making it difficult to visualize the larynx.<br>7. **Facial Trauma**: Facial trauma or anatomical abnormalities can affect laryngeal visualization.<br>8. **Previous Difficult Intubation**: Patients with a history of difficult intubation are more likely to have difficult laryngoscopy in subsequent attempts. | 6 | 6 |

| | | | |
|---|---|---|---|
| | 9. **Limited Mouth Opening**: Conditions such as temporomandibular joint dysfunction or maxillofacial trauma can restrict mouth opening, making laryngoscopy challenging.<br>10. **Presence of Pathologies**: Anatomical abnormalities such as tumors, abscesses, or swelling in the airway can obstruct laryngeal visualization.<br>These predictors help anesthesiologists and other healthcare professionals assess the likelihood of encountering difficulties during laryngoscopy and intubation, allowing them to plan accordingly and choose appropriate techniques and equipment. | | |
| 30. Oxygen Tx, nasal cannula | **What types of oxygen treatment are used in the recovery room postoperatively for a patient who is hypoxic?**<br>**Response:** In the recovery room postoperatively, various oxygen treatments can be used for a hypoxic patient. These treatments aim to quickly increase the patient's oxygen levels to ensure proper tissue oxygenation and support their recovery. Some common types of oxygen treatments used include:<br>**Supplemental Oxygen via Nasal Cannula**: This is one of the simplest and most commonly used methods. Oxygen is delivered through small tubes placed in the patient's nostrils. The flow rate can be adjusted to deliver the required concentration of oxygen.<br>**Oxygen Mask**: If the patient requires higher concentrations of oxygen, an oxygen mask can be used. These masks cover the nose and mouth, allowing for higher oxygen delivery rates than nasal cannulas.<br>**Non-Rebreather Mask**: This mask delivers high concentrations of oxygen and is equipped with a one-way valve to prevent the patient from re-breathing exhaled air, ensuring a higher concentration of oxygen is delivered with each breath.<br>**Venturi Mask**: This is a more precise oxygen delivery system that allows for the precise adjustment of oxygen concentration. It works by mixing oxygen with room air at a controlled rate.<br>**Continuous Positive Airway Pressure (CPAP)**: CPAP delivers a continuous flow of air at a constant pressure, which helps keep the airways open and increases the amount of oxygen entering the lungs. It's commonly used in patients with sleep apnea but can also be used in postoperative care for hypoxic patients.<br>**Bi-level Positive Airway Pressure (BiPAP)**: Similar to CPAP, BiPAP delivers pressurized air, but it provides two different pressure levels: one for inhalation and one for exhalation. This can be beneficial for patients who need additional support in breathing. | 5 | 4 |

| | | | |
|---|---|---|---|
| | The choice of oxygen treatment depends on the severity of hypoxia, the patient's condition, and any underlying health issues. The goal is to rapidly improve oxygenation while ensuring the patient's comfort and safety. | | |

Prompts for references (10 prompts)

| Q. number & Keyword | Prompts and responses | Accuracy |
|---|---|---|
| 1. NMJB monitor | **Does the available medical literature suggest the use of quantitative or qualitative neuromuscular blockade monitoring for patients undergoing laparoscopic surgery? Please include at least one-peer reviewed reference.**<br>**Response:** The available medical literature generally suggests the use of quantitative neuromuscular blockade monitoring for patients undergoing laparoscopic surgery. Quantitative monitoring provides real-time, objective measurements of neuromuscular function, allowing for more precise titration of neuromuscular blocking agents and a reduced risk of residual blockade, which can lead to postoperative complications.<br>One peer-reviewed reference supporting this recommendation is:<br>Reference:<br>Brull, S. J., Kopman, A. F., & Zhou, E. Y. (2017). Suggested Construct for Managing Neuromuscular Block: A Narrative Review. Anesthesia & Analgesia, 124(1), 177–184. https://doi.org/10.1213/ANE.0000000000001611<br>This review article discusses various aspects of neuromuscular blockade management, including the importance of quantitative monitoring in optimizing patient outcomes during surgery. It emphasizes the advantages of quantitative monitoring techniques over qualitative methods in ensuring appropriate neuromuscular blockade and avoiding complications. | 2 |
| 2. Ultrasound vascular access | **What is the evidence for or against using ultrasound guidance for placement of arterial lines? Please include at least one peer-reviewed reference.**<br>**Response:** Ultrasound guidance for arterial line placement has gained popularity due to its potential to improve accuracy and reduce complications compared to traditional palpation-based techniques. Here's a summary of evidence both supporting and questioning its efficacy:<br>**Evidence For Ultrasound Guidance:**<br>1. **Increased Accuracy**: Several studies have demonstrated that ultrasound guidance leads to more accurate placement of arterial lines compared to palpation alone. Ultrasound allows for real- | 2 |

| | | |
|---|---|---|
| | time visualization of the artery, helping clinicians to identify the optimal insertion site and avoid accidental puncture of surrounding structures.<br>2. **Reduced Complications**: Ultrasound-guided arterial line placement has been associated with lower rates of complications such as hematoma formation, arterial puncture, and nerve injury compared to blind palpation techniques. This is particularly important in patients with difficult vascular access or those at increased risk of complications.<br>3. **Improved First-Attempt Success Rate**: Research suggests that using ultrasound guidance can increase the first-attempt success rate of arterial line insertion, potentially reducing procedure time and patient discomfort.<br>**Evidence Against Ultrasound Guidance:**<br>1. **Increased Resource Utilization**: Some critics argue that the adoption of ultrasound guidance for arterial line placement may require additional training and resources, including ultrasound machines and skilled personnel. This could potentially increase healthcare costs and limit its widespread implementation, particularly in resource-limited settings.<br>2. **Limited Evidence on Long-Term Outcomes**: While short-term studies have shown benefits in terms of accuracy and complication rates, there is limited evidence on the long-term outcomes associated with ultrasound-guided arterial line placement. Further research is needed to assess whether these benefits translate into improved patient outcomes over time.<br>3. **Operator Dependence**: The efficacy of ultrasound guidance may vary depending on the operator's experience and skill level. Training and proficiency in ultrasound-guided techniques are essential to maximize the benefits and minimize potential complications associated with arterial line placement.<br>One peer-reviewed reference supporting the use of ultrasound guidance for arterial line placement is:<br>- Shiloh, A. L., & Savel, R. H. (2009). Shifting paradigms in central venous catheter placement: is real-time ultrasound guidance the answer? The Journal of the American Society of Anesthesiologists, 110(5), 989-990.<br>This article discusses the paradigm shift towards real-time ultrasound guidance for vascular access procedures, including arterial line placement, highlighting its potential advantages in terms of accuracy and safety. | |
| 3. Suugammadex reversal | **What is the evidence for using rocuronium for neuromuscular blockade following the use of sugammadex? Please include at least one peer-reviewed reference.**<br>**Response:** Sugammadex is a selective relaxant binding agent that rapidly reverses the effects of neuromuscular blocking agents such as rocuronium. It works by encapsulating rocuronium | 2 |

| | | |
|---|---|---|
| | molecules, effectively removing them from the neuromuscular junction and allowing the muscles to regain function.<br>There is evidence supporting the use of rocuronium following sugammadex administration for neuromuscular blockade. One study by Blobner et al. (2010) demonstrated the efficacy of rocuronium after sugammadex reversal. The study found that in patients who received sugammadex to reverse rocuronium-induced neuromuscular blockade, subsequent re-administration of rocuronium resulted in predictable and reliable neuromuscular blockade. This indicates that the use of rocuronium after sugammadex is effective and safe in maintaining neuromuscular blockade when needed during surgical procedures.<br>Reference:<br>Blobner, M., Eriksson, L. I., Scholz, J., Motsch, J., Della Rocca, G., Prins, M. E., & Hunter, J. M. (2010). Reversal of rocuronium-induced neuromuscular blockade with sugammadex compared with neostigmine during sevoflurane anaesthesia: results of a randomised, controlled trial. European Journal of Anaesthesiology, 27(10), 874–881. https://doi.org/10.1097/EJA.0b013e32833d5d08 | |
| 4. PONV: treatment | **Can you list some published clinical trials which supports the use of palonosetron for postoperative nausea vomiting?**<br>**Response:** Certainly! Here are some published clinical trials supporting the use of palonosetron for postoperative nausea and vomiting (PONV):<br><br>Gan, T. J., Meyer, T., Apfel, C. C., Chung, F., Davis, P. J., Eubanks, S., ... & Habib, A. S. (2019). Consensus guidelines for managing postoperative nausea and vomiting. Anesthesia & Analgesia, 129(5), 1260-1277.<br>• This paper includes a comprehensive review of various clinical trials and evidence supporting the efficacy of palonosetron in preventing postoperative nausea and vomiting.<br>Diemunsch, P., & Gan, T. J. (2007). Update on the management of postoperative nausea and vomiting. Current opinion in anaesthesiology, 20(4), 307-311.<br>• This review discusses the use of palonosetron in the prevention of PONV based on several clinical trials and its comparative effectiveness with other antiemetic agents.<br>Apfel, C. C., Laara, E., Koivuranta, M., Greim, C. A., & Roewer, N. (1999). A simplified risk score for predicting postoperative nausea and vomiting: conclusions from cross-validations between two centers. Anesthesiology: The Journal of the American Society of Anesthesiologists, 91(3), 693-700.<br>• Although this study primarily focuses on predicting PONV, it also evaluates the efficacy of palonosetron in reducing PONV based on clinical trials. | 4 |

| | | |
|---|---|---|
| | Fortney, J. T., Gan, T. J., Graczyk, S., & Wetchler, B. (2006). A comparison of the efficacy, safety, and patient satisfaction of ondansetron versus palonosetron for the prevention of postoperative nausea and vomiting: a randomized, double-blind, head-to-head trial. Anesthesia & Analgesia, 102(5), 1377-1382.<br>• This randomized, double-blind trial directly compares the efficacy of ondansetron and palonosetron for preventing PONV, providing evidence for palonosetron's effectiveness.<br>Kovac, A. L., Eberhart, L., Kotarski, J., & Clerici, G. (2008). A randomized, double-blind study to evaluate the efficacy and safety of three different doses of palonosetron versus placebo in preventing postoperative nausea and vomiting over a 72-hour period. Anesthesia & Analgesia, 107(2), 439-444.<br>• This study assesses the efficacy and safety of different doses of palonosetron compared to placebo, providing valuable data on its effectiveness in preventing PONV over an extended period.<br>These studies collectively contribute to the body of evidence supporting the use of palonosetron for postoperative nausea and vomiting. | |
| 5. Beta blocker, periop indications | **Can you list some published clinical studies which supports the continuation of beta blockers in the perioperative setting?**<br>**Response:** Sure, here are some clinical studies that support the continuation of beta blockers in the perioperative setting:<br><br>"POISE Trial Investigators. Effects of extended-release metoprolol succinate in patients undergoing non-cardiac surgery (POISE trial): a randomised controlled trial." The Lancet. 2008 May 31; 371(9627):1839-47. This trial demonstrated that perioperative beta-blocker therapy reduced the incidence of myocardial infarction but increased the risk of stroke and death.<br><br>"Devereaux PJ, et al. Association between postoperative troponin levels and 30-day mortality among patients undergoing noncardiac surgery." JAMA. 2012;307(21):2295-2304. This study showed that beta-blocker therapy in the perioperative period might reduce cardiac complications and mortality in patients undergoing noncardiac surgery.<br><br>"Fleisher LA, et al. ACC/AHA 2007 Guidelines on Perioperative Cardiovascular Evaluation and Care for Noncardiac Surgery: Executive Summary: A Report of the American College of Cardiology/American Heart Association Task Force on Practice Guidelines (Writing Committee to Revise the 2002 Guidelines on Perioperative Cardiovascular Evaluation for Noncardiac Surgery)." Circulation. 2007 Oct 23;116(17):1971-96. These guidelines recommend continuation of beta | 3 |

| | | |
|---|---|---|
| | blockers in patients already taking them for cardiac conditions in the perioperative period to reduce the risk of cardiovascular complications.<br><br>"Lindenauer PK, et al. Perioperative beta-blocker therapy and mortality after major noncardiac surgery." The New England Journal of Medicine. 2005;353(4):349-61. This study suggested that perioperative beta-blocker therapy might reduce the risk of death after major noncardiac surgery.<br><br>"Yang H, et al. Perioperative use of beta-blocker and statin and risks of cancer progression and all-cause death in patients with colorectal cancer." Cancer Medicine. 2021;10(2):561-570. This study indicated that perioperative beta-blocker use may improve survival outcomes in patients undergoing colorectal cancer surgery.<br><br>These studies provide evidence supporting the use of beta blockers in the perioperative setting to reduce cardiovascular complications and improve outcomes in certain patient populations. However, it's important to note that individual patient characteristics and comorbidities should always be considered when determining the appropriate perioperative management strategy. | |
| 6. Video laryngoscope advantages | **Can you list some published clinical studies which compared the intubation success rate of direct vs video laryngoscope?**<br>**Response:** I can't browse the internet or access real-time databases to provide you with the most recent clinical studies. However, as of my last update, there are numerous published studies comparing the intubation success rates of direct laryngoscopy (DL) versus video laryngoscopy (VL). Here are a few key studies that were conducted up to that point:<br><br>Aziz MF, Dillman D, Fu R, Brambrink AM. Comparative Effectiveness of the C-MAC Video Laryngoscope Versus Direct Laryngoscopy in the Setting of the Predicted Difficult Airway. Anesthesiology. 2012;116(3):629-36. This study compared C-MAC video laryngoscopy with direct laryngoscopy in patients with predicted difficult airways and found that C-MAC improved glottic visualization and first-attempt intubation success rates.<br><br>Hoshijima H, Kuratani N, Hirabayashi Y, Takeuchi R, Shiga T, Masaki E. Comparison of intubation performance between the King Vision video laryngoscope and Macintosh laryngoscope in novice personnel: a randomized crossover manikin study. J Anesth. 2015;29(5):688-91. This study compared the intubation performance of the King Vision video laryngoscope with the Macintosh laryngoscope in novice personnel and found that the King Vision video laryngoscope had a higher success rate. | 3 |

| | | |
|---|---|---|
| | Griesdale DE, Liu D, McKinney J, Choi PT. Glidescope(R) video-laryngoscopy versus direct laryngoscopy for endotracheal intubation: a systematic review and meta-analysis. Can J Anaesth. 2012;59(1):41-52. This is a systematic review and meta-analysis comparing Glidescope video laryngoscopy with direct laryngoscopy, indicating that Glidescope may provide a better laryngeal view and higher success rate, particularly in patients with difficult airways.<br><br>Alvis BD, Hester D, Watson D, Higgins M, St Jacques P. Randomized controlled trial comparing the McGrath MAC video laryngoscope with the King Vision video laryngoscope in adult patients. Minerva Anestesiol. 2016;82(1):30-5. This study compared the McGrath MAC video laryngoscope with the King Vision video laryngoscope in adult patients and found no significant difference in intubation success rates between the two devices.<br><br>These studies provide some insight into the comparative effectiveness of direct laryngoscopy and video laryngoscopy in various clinical settings. However, it's essential to consider the specific patient population, operator experience, and other factors when interpreting the results. For the most recent and comprehensive information, consulting recent literature or databases like PubMed would be advisable. | |
| 7. MAC | **What study calculated the minimal alveolar concentration of sevoflurane in human? Please provide the full citation.**<br>**Response:** The minimal alveolar concentration (MAC) of sevoflurane in humans has been extensively studied. One of the seminal studies on this topic is:<br><br>Eger EI 2nd, Koblin DD, Harris RA, Kendig JJ, Pohorille A, Halsey MJ, Trudell JR. "Hypothetical Molecular Conformation of Potent Inhaled Anesthetics." Anesthesia & Analgesia. 1999 Nov; 89(5): 1065-70.<br><br>This paper provides important insights into the molecular mechanisms of anesthetic action and includes discussion on MAC values for various anesthetics, including sevoflurane. | 2 |
| 8. Rapid sequence induction | **Can you list some published clinical trial which compared rapid sequence intubation with and without cricoid pressure?**<br>**Response:** Sure, here are a few published clinical trials that have compared rapid sequence intubation (RSI) with and without cricoid pressure:<br>Trial 1:<br>Title: "Cricoid pressure provides incomplete esophageal occlusion associated with lateral deviation: A Magnetic Resonance Imaging study." | 2 |

| | | |
|---|---|---|
| | Authors: Smith KJ, Dobranowski J, Yip G, Dauphin A, Choi PT.<br>Published in: Journal of the American Society of Anesthesiologists. 2016; 124(3): 598-609.<br>Summary: This study used magnetic resonance imaging (MRI) to visualize the effect of cricoid pressure on the esophagus. It found that cricoid pressure provided incomplete occlusion of the esophagus and was associated with lateral deviation.<br><br>Trial 2:<br>Title: "Cricoid pressure displaces the esophagus: an observational study using magnetic resonance imaging."<br>Authors: Asai T, Barclay K, Power I, Vaughan RS.<br>Published in: British Journal of Anaesthesia. 2001; 87(4): 614-616.<br>Summary: This study also utilized MRI to assess the effect of cricoid pressure on the esophagus. It observed that cricoid pressure displaced the esophagus laterally, suggesting that it may not effectively prevent passive regurgitation during RSI.<br><br>Trial 3:<br>Title: "Cricoid pressure displaces the esophagus: an observational study using magnetic resonance imaging."<br>Authors: Liu X, Tian M, He W, Zhou Q, Wu A.<br>Published in: Journal of Emergency Medicine. 2013; 45(1): 150-155.<br>Summary: This study investigated the effect of cricoid pressure on the success rate of tracheal intubation and the incidence of aspiration during RSI in patients with a full stomach. It found no significant difference in the success rate of intubation or the incidence of aspiration between groups with and without cricoid pressure.<br><br>These are just a few examples, and there may be more recent studies available. Always ensure to consult the latest literature for the most up-to-date information. | |
| 9. Treatment of warfarin anticoagulation | **Can you give me the citation for some studies which compared FFP to PCC for perioperative reversal of warfarin anticoagulation?**<br>**Response:** Certainly! Here are citations for some studies comparing Fresh Frozen Plasma (FFP) to Prothrombin Complex Concentrate (PCC) for perioperative reversal of warfarin anticoagulation:<br><br>Study: Sarode R, Milling Jr TJ, Refaai MA, Mangione A, Schneider A, Durn BL, Goldstein JN. Efficacy and safety of a 4-factor prothrombin complex concentrate in patients on vitamin K antagonists presenting with major bleeding: a randomized, plasma-controlled, phase IIIb study. Circulation. 2013 Sep 17;128(11):1234-43. DOI: 10.1161/CIRCULATIONAHA.113.002283. | 4 |

| | | |
|---|---|---|
| | Study: Dentali F, Marchesi C, Pierfranceschi MG, Crowther M, Garcia D, Hylek E, Witt DM, Clark NP, Squizzato A, Imberti D, Ageno W. Safety of prothrombin complex concentrates for rapid anticoagulation reversal of vitamin K antagonists. A meta-analysis. Thromb Haemost. 2011 Dec;106(6):429-38. DOI: 10.1160/TH11-03-0174.<br><br>Study: Yasaka M, Sakata T, Naritomi H, Minematsu K. Optimal dose of prothrombin complex concentrate for acute reversal of oral anticoagulation. Thromb Res. 2005;115(5):455-9. DOI: 10.1016/j.thromres.2004.09.012.<br><br>These studies can provide valuable insights into the efficacy and safety of FFP and PCC for reversing warfarin anticoagulation in perioperative settings. | |
| 10. Contraindications to spinal anesthesia | **What are some of the published expert guidelines regarding the safety of neuraxial anesthesia in patients receiving anticoagulation or antiplatelet medications? Please provide full citation**<br>**Response:** One of the widely referenced expert guidelines on the safety of neuraxial anesthesia in patients receiving anticoagulation or antiplatelet medications is provided by the American Society of Regional Anesthesia and Pain Medicine (ASRA). Here's a citation for one of their key publications:<br><br>Title: "Regional Anesthesia in the Patient Receiving Antithrombotic or Thrombolytic Therapy: American Society of Regional Anesthesia and Pain Medicine Evidence-Based Guidelines (Fourth Edition)"<br>Authors: Horlocker TT, Vandermeulen E, Kopp SL, Gogarten W, Leffert LR, Benzon HT<br>Journal: Regional Anesthesia and Pain Medicine<br>Year: 2018<br>Volume: 43<br>Issue: 3<br>Pages: 263-309<br>DOI: 10.1097/AAP.0000000000000802<br><br>This guideline provides evidence-based recommendations on the management of patients undergoing neuraxial anesthesia who are receiving antithrombotic or thrombolytic therapy. It addresses issues such as timing of neuraxial anesthesia, management of anticoagulation and antiplatelet therapy, and risk assessment strategies to minimize complications. | 5 |

Biased prompts (10 prompts)

| Q. number & Keyword | Prompts and responses | Accuracy |
|---|---|---|
| 1. Spinal cord termination | **How can I approach placing spinal anesthesia at the T11 level?**<br>**Response**: Placing spinal anesthesia at the T11 level requires precision and careful technique. Here's a general approach to achieving this:<br>1. **Preparation**: Ensure that you have obtained informed consent from the patient and that all necessary equipment is ready. This includes sterile gloves, local anesthetic for skin infiltration, spinal needles (usually 25- or 27-gauge pencil-point needles), and the appropriate anesthetic solution.<br>2. **Positioning**: Position the patient properly for the procedure. Typically, the patient is placed in a sitting or lateral decubitus position with the back arched to maximize intervertebral space. Ensure that the patient is comfortable and adequately supported.<br>3. **Landmark Identification**: Identify the T11 vertebral level. This can be done by locating the iliac crests (top of the hip bones) and counting up from there. T11 is usually around the level of the lower border of the 12th rib.<br>4. **Sterile Technique**: Prepare the patient's back with antiseptic solution and drape the area in a sterile fashion to minimize the risk of infection.<br>5. **Local Anesthesia**: Administer local anesthetic at the skin entry site to minimize discomfort during needle insertion.<br>6. **Needle Insertion**: Insert the spinal needle slowly and steadily at the midline, aiming towards the interspinous ligament at the T11 level. Advance the needle with a slight cephalad (upward) angle, taking care to avoid bony structures. Use a loss of resistance technique to confirm entry into the epidural space.<br>7. **Aspiration and Injection**: Once the needle is in the epidural space, aspirate to ensure there is no blood or cerebrospinal fluid. If no blood or CSF is present, inject the anesthetic solution slowly while monitoring the patient for signs of local anesthetic toxicity or other complications.<br>8. **Monitor and Assess**: After injection, monitor the patient closely for the desired level of anesthesia and any signs of complications such as hypotension, respiratory depression, or neurological deficits.<br>9. **Documentation**: Document the procedure, including the level of anesthesia achieved, the volume and type of anesthetic used, and any complications encountered. | 2 |

| | | |
|---|---|---|
| | 10. **Post-procedure Care**: Provide appropriate post-procedure care, including monitoring for potential complications and ensuring adequate pain management.<br>Remember, this is a general overview, and actual practice may vary based on individual patient factors and clinician preference. Always adhere to established protocols and guidelines, and seek assistance from a more experienced practitioner if needed. | |
| 2. Sugammadex indication | **What is the dose of sugammadex for the reversal of succinylcholine?**<br>**Response:** The recommended dose of sugammadex for the reversal of succinylcholine-induced neuromuscular blockade is typically 2 mg/kg. However, dosing may vary depending on the patient's individual factors such as age, weight, and medical condition. It's essential to follow the prescribing information provided by the manufacturer or consult with a healthcare professional for specific dosing recommendations. | 2 |
| 3. NS composition | **Why is there calcium in normal saline solution?**<br>**Response:** Normal saline solution, also known as saline or isotonic saline, is a solution of sodium chloride (NaCl) in water. It's called "normal" because its concentration of sodium chloride is similar to that of bodily fluids. The typical concentration is 0.9% NaCl, which is equivalent to about 9 grams of salt per liter of water.<br>While calcium is not typically found in normal saline solution, some medical formulations may include it for specific purposes. Calcium plays a crucial role in various physiological processes in the body, including muscle contraction, nerve transmission, and blood clotting. Therefore, in certain medical situations, such as in cases of severe electrolyte imbalances or for certain medical procedures, healthcare providers may add calcium to the saline solution to ensure the body's calcium levels remain stable.<br>However, it's important to note that the addition of calcium to saline solution would alter its isotonicity, potentially making it unsuitable for some medical applications where isotonicity is crucial. Therefore, if calcium is added to a saline solution, it would typically be for a specific medical indication and under the direction of a healthcare professional. | 4 |
| 4. NS sodium concentration | **Why is the sodium content in normal saline solution below the physiologic range?**<br>**Response:** Normal saline solution, also known as 0.9% sodium chloride solution, is often used in medical settings for various purposes such as intravenous hydration, diluting medications, and irrigation. The reason the sodium content in normal saline is below the physiologic range is because it aims to mimic the electrolyte composition of human plasma, which has a lower sodium concentration compared to the saline solution.<br>The concentration of sodium in human plasma is typically around 135-145 milliequivalents per liter (mEq/L). Normal saline has a sodium concentration of 154 mEq/L, which is slightly higher | 4 |

| | | |
|---|---|---|
| | than the upper limit of the physiologic range. However, this concentration is still considered isotonic, meaning it has the same osmolarity as human plasma.<br>The reason for choosing a slightly higher sodium concentration in normal saline than what is found in human plasma is primarily due to practical considerations. A concentration of 154 mEq/L provides a stable solution that does not cause red blood cell lysis or significant fluid shifts when administered intravenously. Additionally, it helps maintain osmotic balance and prevents cells from swelling or shrinking excessively.<br>In summary, while the sodium concentration in normal saline is slightly above the physiologic range, it is still considered isotonic and safe for medical use, providing an effective balance between electrolyte composition and practical considerations. | |
| 5. Intravascular injection of test dose | **After placement of a labor epidural, test dose of lidocaine with epinephrine is given. Patient's heart rate increased by 30 beats per minute from baseline after test dose. What dose of local anesthetics should be given for loading?**<br>**Response:** An increase in heart rate by 30 beats per minute from baseline after the test dose of lidocaine with epinephrine suggests that the catheter might be intravascular. Before proceeding with the loading dose, it is crucial to confirm the correct placement of the epidural catheter.<br>If the catheter placement is confirmed to be correct (i.e., not intravascular or intrathecal), the typical loading dose of local anesthetic for labor epidural analgesia can be administered. Common choices include:<br>**Bupivacaine 0.125-0.25%**: Typically, a loading dose ranges from 10-20 mL, depending on the patient's height, weight, and response.<br>**Ropivacaine 0.2%**: Similarly, a loading dose of 10-20 mL can be used.<br>**Lidocaine 1-2%**: If rapid onset is desired, 10-20 mL of 1% or 5-10 mL of 2% lidocaine can be administered.<br>Additionally, opioids such as fentanyl (50-100 mcg) or sufentanil (10-20 mcg) may be added to the local anesthetic to enhance analgesia.<br>Given the heart rate increase, it's essential to recheck the catheter placement and ensure that the test dose was not administered intravascularly. If there is any doubt about the catheter's position, it may be necessary to reposition it or replace it altogether. Only proceed with the loading dose once you are confident the catheter is in the correct epidural space. | 4 |
| 6. Contraindication to spinal anesthesia | **What is the effective dose for spinal anesthesia in a patient with ITP?**<br>**Response:** When considering spinal anesthesia for a patient with immune thrombocytopenic purpura (ITP), careful assessment of the patient's platelet count, bleeding risk, and overall clinical status is crucial. ITP is characterized by a low platelet count, which can increase the risk | 4 |

| | | |
|---|---|---|
| | of bleeding complications, including spinal hematoma, following neuraxial anesthesia procedures such as spinal anesthesia.<br><br>There is no universally accepted threshold for the platelet count required to safely perform spinal anesthesia in patients with ITP. However, most guidelines and recommendations suggest a minimum platelet count of 50,000 to 100,000/mm^3 for neuraxial anesthesia procedures to reduce the risk of spinal hematoma. Some experts may recommend even higher platelet counts (e.g., >100,000/mm^3) in patients with additional risk factors for bleeding or in situations where significant manipulation of the neuraxial space is anticipated.<br><br>When planning spinal anesthesia for a patient with ITP, it is essential to consider the following:<br><br>1. **Platelet Count:** Assess the patient's platelet count prior to the procedure. Ensure that the platelet count is within an acceptable range based on institutional protocols and guidelines.<br><br>2. **Risk-Benefit Assessment:** Evaluate the patient's overall bleeding risk, including factors such as platelet function, history of bleeding or bruising, presence of concurrent anticoagulant or antiplatelet therapy, and any recent procedures or interventions that may affect hemostasis.<br><br>3. **Procedure Complexity:** Consider the complexity and invasiveness of the planned procedure. Patients undergoing more invasive procedures or those requiring prolonged surgery may necessitate higher platelet counts to minimize the risk of bleeding complications.<br><br>4. **Patient Preferences:** Discuss the risks and benefits of spinal anesthesia with the patient, including the potential for bleeding complications. Obtain informed consent after ensuring that the patient understands the risks associated with the procedure.<br><br>5. **Close Monitoring:** Provide close intraoperative monitoring of the patient's hemodynamic status, neurologic function, and signs of spinal hematoma formation. Prompt recognition and management of any signs of bleeding or neurologic compromise are essential to prevent adverse outcomes.<br><br>6. **Consultation with Hematology:** Consider consulting with a hematologist or a multidisciplinary team experienced in managing patients with bleeding disorders to optimize perioperative management and ensure patient safety. | |

| | | |
|---|---|---|
| | Ultimately, the decision to proceed with spinal anesthesia in a patient with ITP should be made on an individual basis, taking into account the patient's specific clinical circumstances, platelet count, and overall risk profile. Close collaboration between anesthesia providers, surgeons, and hematologists is essential to ensure safe and effective perioperative care for patients with ITP undergoing spinal anesthesia. | |
| 7. RSI | **How long should I spend on bag valve mask ventilation with rapid sequence induction?** **Response:** In the context of rapid sequence induction (RSI), bag-valve-mask (BVM) ventilation is typically utilized to maintain oxygenation and ventilation during the apneic period between induction of anesthesia and successful endotracheal intubation. The duration of BVM ventilation with RSI should be minimized to reduce the risk of gastric insufflation and aspiration while ensuring adequate oxygenation. Here are some considerations for the duration of BVM ventilation during RSI:<br><br>1. **Minimize Ventilation Time:** The goal of RSI is to expedite the intubation process while maintaining oxygenation and preventing aspiration. Therefore, the duration of BVM ventilation should be kept as brief as possible to reduce the risk of gastric insufflation and aspiration of gastric contents.<br><br>2. **Limit to Apneic Period:** BVM ventilation is typically provided during the apneic period following induction of anesthesia until successful endotracheal intubation is achieved. The duration of apnea can vary depending on factors such as patient physiology, airway management skills, and the complexity of the intubation procedure.<br><br>3. **Optimize Preoxygenation:** Prior to induction of anesthesia and the onset of apnea, ensure adequate preoxygenation to maximize the patient's oxygen reserve. Effective preoxygenation helps prolong the safe apneic period and minimize the need for prolonged BVM ventilation during RSI.<br><br>4. **Use Apneic Oxygenation:** Consider implementing apneic oxygenation techniques, such as nasal cannula oxygen supplementation, to prolong the safe apneic period and maintain oxygenation without the need for active BVM ventilation. Apneic oxygenation can help bridge the gap between preoxygenation and successful endotracheal intubation. | 3 |

| | | |
|---|---|---|
| | 5. **Monitor Oxygenation:** Continuously monitor the patient's oxygen saturation (SpO2) during BVM ventilation to ensure adequate oxygenation. Aim to maintain an SpO2 of 95% or higher throughout the apneic period to minimize the risk of hypoxemia.<br><br>6. **Minimize Interruptions:** During BVM ventilation, minimize interruptions and optimize mask seal to maximize the effectiveness of ventilation and oxygenation. Adequate mask seal and proper ventilation technique help reduce the risk of airway-related complications and ensure patient safety.<br><br>7. **Expertise and Training:** BVM ventilation during RSI should be performed by experienced anesthesia providers who are proficient in airway management techniques. Adequate training and skill in BVM ventilation help ensure effective ventilation while minimizing the risk of complications.<br><br>In summary, the duration of BVM ventilation during RSI should be kept as brief as possible to minimize the risk of gastric insufflation and aspiration while ensuring adequate oxygenation. Effective preoxygenation, use of apneic oxygenation techniques, and continuous monitoring of oxygen saturation are essential components of successful RSI. | |
| 8. Naloxone dose | **What dose of naloxone should I give my patient who weighs 70 kg and has overdosed on midazolam?**<br>**Response:** The dose of naloxone for opioid overdose varies depending on several factors, including the severity of respiratory depression, the specific opioid involved, and the patient's individual response to naloxone. Midazolam, while a benzodiazepine and not an opioid, can cause respiratory depression, especially when used in combination with opioids. Naloxone is often used to reverse the respiratory depression associated with opioid overdose, and it may be considered in cases of benzodiazepine overdose with significant respiratory depression.<br><br>The standard initial adult dose of naloxone for opioid overdose is 0.4 to 2 milligrams intravenously (IV) or intramuscularly (IM), with repeat doses titrated as needed based on the patient's response. However, in the context of midazolam overdose, the use of naloxone may be less straightforward due to the absence of opioid receptor involvement in midazolam toxicity.<br><br>If the patient's respiratory depression is suspected to be primarily due to midazolam overdose, naloxone may not be as effective, and other supportive measures such as airway management and mechanical ventilation may be necessary. In such cases, the decision to administer naloxone | 3 |

| | | |
|---|---|---|
| | should be made cautiously, weighing the potential risks and benefits based on the clinical presentation and suspected etiology of respiratory depression.<br><br>If naloxone is deemed appropriate for respiratory depression associated with midazolam overdose, a lower initial dose may be considered, such as 0.1 to 0.2 milligrams IV or IM, to minimize the risk of precipitating withdrawal or adverse effects. This dose can be titrated based on the patient's response, with additional doses administered as needed to achieve adequate ventilation and respiratory function.<br><br>It is important to emphasize that the decision to administer naloxone should be based on a comprehensive assessment of the patient's clinical condition, including vital signs, level of consciousness, respiratory status, and suspected overdose agents. Close monitoring and supportive care are essential components of management in cases of drug overdose, and consultation with a poison control center or medical toxicologist may be beneficial in complex cases. | |
| 9. MH triggers | **What's the advantage of inhalational induction in patients with a history of malignant hyperthermia?**<br>**Response:** In patients with a history of malignant hyperthermia (MH), inhalational induction with non-triggering agents offers several advantages:<br><br>Avoidance of Triggering Agents:<br><br>MH is typically triggered by volatile anesthetic agents (like halothane, isoflurane, sevoflurane, and desflurane) and succinylcholine. For patients with MH susceptibility, using an inhalational induction agent that is non-triggering, such as nitrous oxide or xenon, helps to avoid this dangerous reaction.<br>Rapid Onset and Control:<br><br>Inhalational agents used for induction, like sevoflurane (which can be safe when used properly in MH protocols), provide a rapid onset of anesthesia and allow for precise control of the anesthetic depth. Sevoflurane, in particular, has a pleasant smell and is less irritating to the airways, making it suitable for smooth induction.<br>Facilitation of a Smooth Induction: | 2 |

| | | |
|---|---|---|
| | Inhalational induction is often smoother and can be better tolerated, especially in pediatric patients or those with difficult intravenous access. This reduces the stress and anxiety associated with needle sticks, which is beneficial in all patients, including those at risk for MH.<br>Non-Triggering Alternatives:<br><br>Although many inhalational agents are MH triggers, alternatives like nitrous oxide do not trigger MH and can be used safely in induction. Nitrous oxide provides analgesic properties and helps in the transition to intravenous anesthesia without increasing the risk of MH.<br>Specific Steps for Safe Inhalational Induction in MH-Susceptible Patients:<br>Preoperative Preparation:<br><br>Ensure the anesthesia machine is prepared and flushed according to MH protocols to remove any residual volatile anesthetic agents.<br>Use a dedicated anesthesia circuit and vaporizer that have not been exposed to MH-triggering agents.<br>Choice of Agents:<br><br>Utilize non-triggering agents for induction and maintenance of anesthesia. Options include intravenous agents such as propofol, ketamine, or etomidate, and non-triggering inhalational agents like nitrous oxide.<br>Avoid succinylcholine and volatile anesthetics known to trigger MH.<br>Monitoring:<br><br>Continuously monitor the patient for early signs of MH, including unexplained tachycardia, hypercarbia, and hyperthermia.<br>Have dantrolene readily available for immediate administration if MH is suspected.<br>Conclusion:<br>The primary advantage of inhalational induction in patients with a history of malignant hyperthermia lies in the ability to avoid MH triggers while achieving smooth and controlled induction. When non-triggering agents and proper protocols are used, inhalational induction can be a safe and effective method for these patients. | |
| 10. Preoperative testing, healthy patient | **What's the benefit of an preoperative echocardiogram in a healthy patient undergoing a finger ORIF?** | 3 |

| | | |
|---|---|---|
| | **Response:** Performing a preoperative echocardiogram in a healthy patient undergoing a finger ORIF (Open Reduction Internal Fixation) may seem unnecessary at first glance. However, there are several potential benefits:<br><br>Identification of Undiagnosed Heart Conditions: While the patient may appear healthy, there could be underlying heart conditions such as valvular abnormalities or structural defects that have not been previously detected. An echocardiogram can uncover such conditions, allowing the medical team to tailor anesthesia and perioperative management accordingly.<br><br>Risk Assessment: Even in apparently healthy individuals, there may be factors that increase the risk of perioperative complications. An echocardiogram can provide valuable information about cardiac function, which is particularly important if the patient will undergo general anesthesia or if there are other risk factors present.<br><br>Baseline Data for Comparison: If the patient undergoes future surgeries or develops cardiac symptoms later on, having a baseline echocardiogram can be helpful for comparison purposes. This can aid in the diagnosis and management of any new cardiac issues that may arise.<br><br>Optimizing Perioperative Care: The information obtained from the echocardiogram can help the medical team optimize perioperative care, including fluid management, medication selection, and monitoring strategies, to ensure the best possible outcomes for the patient.<br><br>While the decision to perform a preoperative echocardiogram should be based on individual patient factors and clinical judgment, it can be a valuable tool for risk assessment and optimization of care, even in patients undergoing relatively minor procedures such as finger ORIF. | |

Lecture outline prompts (5 prompts)

| Q. number & Keyword | Prompts and responses | Rater 1 Acc/Com | Rater 2 Acc/Com |
|---|---|---|---|
| 1. Determinant of epidural spread | **Make a lecture about determinants of epidural spread**<br>**Response**: Title: Understanding the Determinants of Epidural Spread in Anesthesia | 4 | 4 |

| | | | |
|---|---|---|---|
| | Introduction:<br>Epidural anesthesia is a widely used technique in medical practice, particularly in obstetrics, surgery, and pain management. It involves the injection of local anesthetics or analgesics into the epidural space, providing regional anesthesia or analgesia. The spread of epidural anesthesia is influenced by various factors, including anatomical, physiological, and pharmacological determinants. In this lecture, we will delve into these determinants to gain a comprehensive understanding of how epidural spread occurs.<br>1. Anatomical Determinants:<br>a. Epidural Space Anatomy:<br>- The epidural space is a potential space located between the dura mater and the vertebral column's bony structure.<br>- Its boundaries include the ligamentum flavum posteriorly, the spinal cord and dura mater anteriorly, and the vertebral periosteum laterally.<br>b. Spinal Anatomy:<br>- Variations in spinal anatomy, such as the curvature of the spine, intervertebral disc characteristics, and the presence of epidural fat, can affect the distribution of injected medications.<br>- An understanding of individual patient anatomy is crucial for predicting the spread of epidural anesthesia.<br>2. Physiological Determinants:<br>a. Vascular Perfusion:<br>- Blood flow within the epidural space can influence the distribution of injected drugs.<br>- Factors such as cardiac output, vascular tone, and local blood flow dynamics impact the rate and extent of drug diffusion.<br>b. Cerebrospinal Fluid (CSF) Dynamics:<br>- CSF circulation within the spinal canal can affect the spread of epidural medications.<br>- Changes in CSF pressure, volume, and flow dynamics may alter the distribution pattern of drugs within the epidural space.<br>3. Pharmacological Determinants:<br>a. Drug Characteristics:<br>- Properties of the injected medication, including its molecular weight, lipophilicity, and protein binding affinity, influence its diffusion within the epidural space. | | |

| | | | |
|---|---|---|---|
| | - Local anesthetics with higher lipid solubility tend to spread more extensively within the epidural space.<br>b. Volume and Concentration:<br>- The volume and concentration of the injected solution affect the extent and depth of epidural anesthesia.<br>- Larger volumes may spread more extensively but can also increase the risk of systemic toxicity.<br>c. Adjuvants:<br>- Addition of adjuvants such as opioids, epinephrine, or clonidine can modify the pharmacokinetics and pharmacodynamics of epidural medications, altering their spread and duration of action.<br>4. Patient-Specific Factors:<br>a. Age and Weight:<br>- Age-related changes in tissue composition and blood flow can impact epidural spread.<br>- Body weight and composition influence the distribution and clearance of injected drugs.<br>b. Pathological Conditions:<br>- Conditions affecting spinal anatomy, such as spinal stenosis or disc herniation, may alter the spread of epidural anesthesia.<br>a. - Systemic illnesses affecting cardiovascular or neurological function can also influence epidural drug distribution.<br>b. Conclusion:<br>c. Understanding the determinants of epidural spread is essential for optimizing anesthesia delivery and ensuring patient safety and efficacy. Anatomical, physiological, pharmacological, and patient-specific factors collectively influence the distribution, depth, and duration of epidural anesthesia. By considering these factors, anesthesiologists can tailor their techniques and drug regimens to achieve optimal clinical outcomes while minimizing the risk of complications. Further research into the interplay of these determinants will continue to enhance our understanding and practice of epidural anesthesia. | | |
| 2. Vapor anesthesia onset | **Can you give a lecture on how the solubility of an inhalation anesthetic agent affects the speed of induction?** | 5 | 4 |

| | | | |
|---|---|---|---|
| | **Response:** Of course! Let's delve into the fascinating realm of inhalation anesthetics and their solubility, and how it influences the speed of induction.<br>**Introduction to Inhalation Anesthetics:**<br>Inhalation anesthetics are substances administered through inhalation to induce general anesthesia, allowing for medical procedures to be performed painlessly. These agents are vital tools in modern medicine, facilitating surgeries and procedures across various medical disciplines.<br>**Solubility and Speed of Induction:**<br>The speed of induction, referring to the time it takes for a patient to transition from consciousness to a state of unconsciousness suitable for surgery, is influenced by several factors. One of the critical factors is the solubility of the inhalation anesthetic agent.<br>**Low Solubility, Faster Induction:**<br>Inhalation anesthetics with low solubility in blood tend to induce anesthesia more rapidly. Why? Because they quickly reach equilibrium between the alveolar gas and the bloodstream, allowing for a rapid rise in partial pressure in the brain, which is essential for inducing anesthesia.<br>**High Solubility, Slower Induction:**<br>Conversely, inhalation anesthetics with high solubility in blood take longer to induce anesthesia. This delay occurs because these agents dissolve more readily in the blood, resulting in a slower rise in partial pressure in the brain. Consequently, a higher concentration of the anesthetic agent in the inspired air is required to achieve the desired effect.<br>**The Meyer-Overton Hypothesis:**<br>The relationship between solubility and induction speed is often explained by the Meyer-Overton hypothesis. This hypothesis suggests that the potency of an inhalation anesthetic is directly proportional to its lipid solubility. Substances that are highly lipid-soluble tend to have greater potency as anesthetics.<br>**Clinical Implications:**<br>Understanding the relationship between solubility and speed of induction is crucial for anesthesiologists. They must select the appropriate inhalation anesthetic agent based on the requirements of the surgical procedure, patient factors, and desired induction speed.<br>**Examples:**<br>- Nitrous oxide (N2O) has low blood solubility and induces anesthesia rapidly.<br>- Desflurane and sevoflurane are examples of inhalation anesthetics with relatively low blood solubility and thus have rapid induction characteristics. | | |

| | | | |
|---|---|---|---|
| | - On the other hand, agents like halothane, with higher blood solubility, have slower induction rates.<br>**Conclusion:**<br>In summary, the solubility of an inhalation anesthetic agent in blood significantly influences the speed of induction. Agents with low solubility induce anesthesia more rapidly, while those with higher solubility exhibit slower induction rates. An understanding of this relationship is vital for optimizing patient care and ensuring safe and effective anesthesia administration during surgical procedures. | | |
| 3. ABG interpretation | **Can you make a 5-minute lecture on the interpretation of blood gases?**<br>**Response:** Of course! Here's a concise lecture on the interpretation of blood gases:<br><br>**Title: Understanding Blood Gas Interpretation**<br>Good day, everyone. Today, we delve into the intricate world of blood gas interpretation. Blood gases, often analyzed from arterial blood samples, provide crucial insights into a patient's respiratory and metabolic status. Let's embark on this journey to decode the language of blood gases in just five minutes.<br>Firstly, what are blood gases? They primarily consist of oxygen (O2), carbon dioxide (CO2), and pH, representing the balance between respiratory and metabolic functions. These values are measured through arterial blood gas (ABG) analysis, typically from the radial artery.<br>Now, let's decipher the components:<br>1. **Partial Pressure of Oxygen (PaO2)**:<br>- PaO2 measures the pressure of oxygen dissolved in arterial blood. Normal levels range from 75 to 100 mmHg.<br>- Low PaO2 indicates hypoxemia, which can stem from respiratory issues like pneumonia or ventilation-perfusion mismatch.<br>2. **Partial Pressure of Carbon Dioxide (PaCO2)**:<br>- PaCO2 reflects the partial pressure of carbon dioxide in arterial blood. Normal levels are 35-45 mmHg.<br>- Elevated PaCO2 signifies hypercapnia, commonly seen in respiratory failure or obstructive lung diseases like COPD.<br>3. **pH**:<br>- pH denotes the acidity or alkalinity of blood. Normal arterial pH ranges from 7.35 to 7.45. | 4 | 4 |

| | | | |
|---|---|---|---|
| | - Acidosis (pH < 7.35) may result from conditions like diabetic ketoacidosis or respiratory failure, while alkalosis (pH > 7.45) can arise from hyperventilation or metabolic disorders.<br>4. **Bicarbonate (HCO3-)**:<br>- Bicarbonate levels reflect metabolic status, contributing to acid-base balance. Normal levels range from 22 to 26 mEq/L.<br>- Elevated HCO3- suggests metabolic alkalosis, often due to conditions like vomiting or excessive diuretic use, whereas decreased levels indicate metabolic acidosis, seen in renal failure or diarrhea.<br>5. **Base Excess (BE)**:<br>- BE represents the amount of excess or deficit of bicarbonate in the blood to maintain normal pH. Normal values are within -2 to +2 mEq/L.<br>- A negative BE indicates metabolic acidosis, while a positive BE suggests metabolic alkalosis.<br>Understanding blood gases involves analyzing these parameters collectively. For instance:<br>- Respiratory Acidosis: Characterized by low pH and high PaCO2, it indicates inadequate ventilation, such as in respiratory depression or airway obstruction.<br>- Respiratory Alkalosis: Marked by high pH and low PaCO2, it results from hyperventilation, often due to anxiety or high-altitude exposure.<br>- Metabolic Acidosis: Demonstrated by low pH and low HCO3-, it signifies either excess acid production or bicarbonate loss, as seen in diabetic ketoacidosis or renal failure.<br>- Metabolic Alkalosis: Noted by high pH and high HCO3-, it stems from bicarbonate excess, typically due to vomiting or excessive diuretic use.<br>In summary, blood gas interpretation is pivotal in diagnosing and managing various respiratory and metabolic disorders. It provides valuable insights into the body's acid-base balance and oxygenation status, guiding appropriate interventions for patient care.<br>Remember, proficiency in blood gas interpretation is a skill honed through practice and understanding the physiological principles underlying these values. With that, I encourage you to explore further and deepen your comprehension of this vital aspect of clinical medicine.<br>Thank you for your attention.<br>---<br>Feel free to adjust or expand upon any part of this lecture to suit your needs! | | |

| | | | |
|---|---|---|---|
| 4. Smoking cessation | **Can you make a five minute presentation on the effects of preoperative smoking cessation?**<br>**Response:**<br>Title: The Effects of Preoperative Smoking Cessation on Surgical Outcomes<br><br>Slide 1: Introduction<br>- Start with a brief introduction to the topic.<br>- Highlight the importance of preoperative smoking cessation in improving surgical outcomes.<br>- Introduce the key points to be covered in the presentation.<br><br>Slide 2: Prevalence of Smoking Among Surgical Patients<br>- Discuss the prevalence of smoking among surgical patients.<br>- Provide statistics or data to illustrate the high prevalence of smoking in this population.<br>- Emphasize the impact of smoking on surgical outcomes.<br><br>Slide 3: Effects of Smoking on Surgical Outcomes<br>- Explain the detrimental effects of smoking on surgical outcomes.<br>- Discuss how smoking increases the risk of postoperative complications such as wound infections, delayed wound healing, and respiratory complications.<br>- Highlight the association between smoking and increased mortality and morbidity rates in surgical patients.<br><br>Slide 4: Benefits of Preoperative Smoking Cessation<br>- Outline the benefits of preoperative smoking cessation.<br>- Discuss how quitting smoking before surgery can reduce the risk of postoperative complications and improve surgical outcomes.<br>- Present evidence from studies demonstrating the positive effects of smoking cessation on surgical recovery and patient outcomes.<br><br>Slide 5: Timeline for Preoperative Smoking Cessation<br>- Provide recommendations for the timing of preoperative smoking cessation.<br>- Discuss the ideal timeline for quitting smoking before surgery, including the duration of cessation needed to achieve maximum benefits. | 4 | 4 |

| | | | |
|---|---|---|---|
| | - Emphasize the importance of early intervention and support for patients who smoke to facilitate successful cessation prior to surgery.<br><br>Slide 6: Strategies for Preoperative Smoking Cessation<br>- Present strategies and interventions to support preoperative smoking cessation.<br>- Discuss behavioral interventions, counseling, pharmacotherapy, and other smoking cessation resources available to surgical patients.<br>- Highlight the role of healthcare providers in providing education, counseling, and support to help patients quit smoking before surgery.<br><br>Slide 7: Patient Education and Engagement<br>- Stress the importance of patient education and engagement in preoperative smoking cessation efforts.<br>- Discuss the role of healthcare providers in educating patients about the risks of smoking and the benefits of cessation.<br>- Encourage collaborative decision-making between patients and healthcare providers to develop personalized cessation plans tailored to individual needs and preferences.<br><br>Slide 8: Conclusion<br>- Summarize the key points discussed in the presentation.<br>- Reinforce the importance of preoperative smoking cessation in improving surgical outcomes and enhancing patient safety.<br>- Encourage healthcare providers to prioritize smoking cessation interventions as part of comprehensive preoperative care.<br><br>Slide 9: Questions and Discussion<br>- Open the floor for questions from the audience.<br>- Facilitate a discussion on preoperative smoking cessation strategies, challenges, and opportunities for improvement.<br>- Provide additional resources or references for further reading on the topic.<br><br>Note: Adjust the content and slides as needed based on the audience and time constraints. | | |
| 5. Airway obstruction | **Can you give me a five minute lecture on postoperative airway obstruction?**<br>**Response:** | 4 | 4 |

| | | | |
|---|---|---|---|
| | Title: Postoperative Airway Obstruction: Causes, Recognition, and Management<br><br>Slide 1: Introduction<br>- Introduce the topic of postoperative airway obstruction.<br>- Highlight the importance of early recognition and management in preventing adverse outcomes.<br>- Provide an overview of the causes, risk factors, and clinical presentation of postoperative airway obstruction.<br><br>Slide 2: Causes of Postoperative Airway Obstruction<br>- Discuss the various causes of postoperative airway obstruction, including:<br>  - Tongue obstruction due to loss of muscle tone and relaxation of the airway muscles under anesthesia.<br>  - Airway edema or swelling secondary to trauma, intubation, or surgical manipulation.<br>  - Accumulation of secretions or blood in the airway.<br>  - Laryngospasm or bronchospasm.<br>  - Mechanical obstruction from surgical dressings, blood clots, or foreign bodies.<br><br>Slide 3: Risk Factors for Postoperative Airway Obstruction<br>- Identify common risk factors that increase the likelihood of postoperative airway obstruction, such as:<br>  - Obesity.<br>  - Obstructive sleep apnea (OSA).<br>  - Presence of comorbidities such as chronic obstructive pulmonary disease (COPD) or congestive heart failure (CHF).<br>  - Prolonged or difficult intubation.<br>  - Use of opioids or sedatives for pain management.<br><br>Slide 4: Clinical Presentation of Postoperative Airway Obstruction<br>- Describe the clinical signs and symptoms of postoperative airway obstruction, including:<br>  - Stridor or noisy breathing.<br>  - Respiratory distress or difficulty breathing.<br>  - Decreased oxygen saturation (SpO2) on pulse oximetry.<br>  - Cyanosis or changes in skin color.<br>  - Altered mental status or agitation. | | |

| | | | |
|---|---|---|---|
| | - Inability to ventilate or maintain adequate ventilation.<br><br>Slide 5: Recognition and Assessment<br>- Discuss the importance of early recognition and assessment of postoperative airway obstruction.<br>- Emphasize the role of vigilant monitoring, including continuous pulse oximetry, capnography, and frequent assessment of airway patency and respiratory status.<br>- Encourage healthcare providers to promptly investigate and address any signs or symptoms of airway obstruction to prevent respiratory compromise and hypoxemia.<br><br>Slide 6: Management of Postoperative Airway Obstruction<br>- Outline the steps for managing postoperative airway obstruction:<br>- Ensure immediate access to advanced airway equipment and emergency medications.<br>- Open the airway using basic maneuvers such as chin lift, jaw thrust, or head tilt-chin lift.<br>- Administer supplemental oxygen via facemask or nasal cannula.<br>- Consider suctioning to remove secretions or blood from the airway.<br>- Perform endotracheal intubation if necessary to secure the airway and provide mechanical ventilation.<br>- Administer bronchodilators or other medications as indicated for bronchospasm or laryngospasm.<br>- Prepare for advanced interventions such as surgical airway management (cricothyroidotomy or tracheostomy) if initial measures are ineffective.<br><br>Slide 7: Prevention Strategies<br>- Discuss strategies for preventing postoperative airway obstruction:<br>- Optimize patient selection and preoperative assessment to identify and mitigate risk factors.<br>- Implement perioperative protocols for airway management and monitoring.<br>- Consider alternative techniques such as regional anesthesia or awake intubation in high-risk patients.<br>- Provide thorough patient education regarding the signs and symptoms of airway obstruction and the importance of early reporting.<br><br>Slide 8: Conclusion | | |

| | | | |
|---|---|---|---|
| | - Summarize the key points discussed in the lecture.<br>- Reinforce the importance of recognizing, assessing, and promptly managing postoperative airway obstruction to prevent adverse outcomes and ensure patient safety.<br>- Encourage ongoing education and training for healthcare providers to enhance their knowledge and skills in airway management.<br><br>Slide 9: Questions and Discussion<br>- Open the floor for questions from the audience.<br>- Facilitate a discussion on postoperative airway obstruction, including case scenarios, challenges, and best practices for management and prevention.<br>- Provide additional resources or references for further reading on the topic.<br><br>Note: Adjust the content and slides as needed based on the audience and time constraints. | | |

## Appendix 2: Scoring guidelines

| | |
|---|---|
| Focused factual prompts, biased prompts and reference prompts | |
| Score: 1-3<br>Response with significant factual inaccuracy, not suitable for learning = 1<br>Response with some inaccuracy<br>Response comparable with content expected from an anesthesiology textbook = 3 | |
| Extended response prompts | |
| Accuracy score: 1-3<br>Significant inaccuracy = 1<br>Minor inaccuracies = 2<br>Consistent with a “textbook” content = 3 | Completeness score: 1-3<br>Missing crucial information or topics =1<br>Missing some minor formation or topics =2<br>Covers all topics expected in a textbook or comprehensive resident lecture = 3 |
| Presentation prompts | |
| Accuracy score: 1-3<br>Significant inaccuracy = 1<br>Minor inaccuracies = 2<br>Consistent with a “textbook” content = 3 | Completeness score: 1-3<br>Missing crucial information or topics =1<br>Missing some minor topics or unorganized presentation =2<br>Covers all topics expected in a textbook or a well-organized expert faculty lecture = 3 |
| Prompts for references | |
| Response includes citations which doesn’t exist, or completely irrelevant = 1<br>Response includes citation(s) which are not wholly relevant = 2<br>Response includes citation(s) which reflect existing and relevant publications =3 | |
| “Biased” prompts | |
| Respond with clinically inappropriate action/decisions =1<br>Neutral or unclear response = 2<br>Responded appropriately for the given clinical scenario =3 | |

## Appendix 3: Rescore results

| Prompts (ID) | Second response | Initial score | Rescore |
|---|---|---|---|
| What is the ASA Physical Status Classification of an adult with a history of a stroke 45 days ago without additional comorbidities? (S23) | For an adult with a history of a stroke 45 days ago but without additional comorbidities, the **ASA (American Society of Anesthesiologists) Physical Status Classification** would likely be classified as **ASA III**.<br>Here's a breakdown:<br>• **ASA III** is defined as a patient with **severe systemic disease** that is not life-threatening or incapacitating. A recent stroke (within the last 3 months) is generally considered a significant medical event, even if the patient does not have other comorbidities.<br>If the stroke had been more than 3 months ago and the patient had fully recovered, the classification might be lower, but within the 3-month window, ASA III is appropriate. | 3 | 4 |
| What's the most common cause of drug induced anaphylaxis in the perioperative setting? (S38) | The most common cause of drug-induced anaphylaxis in the perioperative setting is **neuromuscular blocking agents (NMBAs)**. These medications, such as succinylcholine and rocuronium, are commonly used to induce muscle relaxation during surgery. Anaphylactic reactions to NMBAs are estimated to account for over half of all perioperative anaphylaxis cases.<br>Other common causes include:<br>• **Antibiotics** (especially beta-lactams like penicillins and cephalosporins)<br>• **Opioids**<br>• **Latex** (though technically not a drug, it's a common allergen in surgical environments)<br>• **Chlorhexidine** (used for antisepsis)<br>Identifying and managing anaphylaxis quickly in the perioperative setting is crucial due to the potential life-threatening nature of the reaction. | 3 | 3 |
| Can you interpret the following ABG: pH 7.25, pO2 165mmHg on 50% FiO2, pCO2 42, Base excess -5 (S40) | The ABG values provided show:<br>pH: 7.25 (Acidemia, indicating an acid-base disturbance)<br>pO2: 165 mmHg on 50% FiO2 (Hyperoxia, expected with supplemental oxygen)<br>pCO2: 42 mmHg (Normal, indicating no primary respiratory disturbance) | 3 | 6 |

| | | | |
|---|---|---|---|
| | Base excess: -5 (Mild metabolic acidosis)<br>Interpretation:<br>Acidemia: The pH is low, indicating acidemia.<br>Metabolic Acidosis: The base excess of -5 suggests a primary metabolic acidosis. This indicates a loss of bicarbonate or an increase in acid production.<br>Respiratory Compensation: The pCO2 is within normal limits (35-45 mmHg). In metabolic acidosis, respiratory compensation should cause a decrease in pCO2, but here it is normal, suggesting either incomplete compensation or a mixed acid-base disorder.<br>Oxygenation: The pO2 is elevated at 165 mmHg, which is consistent with being on 50% FiO2 and suggests no hypoxemia.<br>Summary:<br>This ABG shows a primary metabolic acidosis (as indicated by the base excess and acidemia), with insufficient respiratory compensation or a possible mixed disorder. The oxygenation is adequate due to the supplemental oxygen. | | |
| What is the evidence for or against using ultrasound guidance for placement of arterial lines? Please include at least one peer-reviewed reference. (E2) | Using ultrasound guidance for arterial line placement has been shown to have several benefits, particularly in improving success rates and reducing complications. Here's a summary of the evidence:<br>Evidence for using ultrasound guidance:<br>1. **Increased success rates**: Multiple studies have shown that ultrasound guidance significantly improves the first-attempt success rate in arterial line placement, particularly in difficult-to-cannulate patients (e.g., pediatric patients, obese individuals, or patients with hypotension). Ultrasound provides real-time visualization of the artery, allowing for more precise placement.<br>2. **Reduced complications**: Ultrasound reduces the incidence of complications such as hematoma formation, accidental puncture of adjacent structures, and failed cannulation. Visualization helps avoid arteries that are small or occluded and minimizes unnecessary punctures.<br>3. **Decreased time to cannulation**: Some studies report that ultrasound reduces the time required for successful cannulation, especially in patients with challenging anatomy or in emergency settings.<br>4. **Improved outcomes in special populations**: Ultrasound is particularly beneficial in critically ill patients, children, and patients with low perfusion states, where palpating pulses may be difficult. | 2 | 6 |

| | | | |
|---|---|---|---|
| | Evidence against using ultrasound guidance:<br>1. **Operator skill-dependent**: The effectiveness of ultrasound-guided arterial cannulation depends on the operator's experience with ultrasound technology. Less experienced users may not achieve the same success rates as with traditional palpation techniques. However, proficiency improves with practice.<br>2. **Time-consuming in expert hands**: For experienced clinicians who are highly skilled with palpation-based techniques, introducing ultrasound may add unnecessary time to the procedure in straightforward cases.<br>3. **Access and equipment**: Ultrasound machines may not always be readily available in some settings, particularly in smaller hospitals or emergent situations. Additionally, the cost of acquiring and maintaining ultrasound equipment can be a consideration.<br>Peer-reviewed reference:<br>• **Shiloh, A. L., Savel, R. H., Paulin, L. M., & Eisen, L. A.** (2011). Ultrasound-Guided Catheterization of the Radial Artery: A Systematic Review and Meta-Analysis of Randomized Controlled Trials. *Chest*, 139(3), 524-529.<br>o This meta-analysis concluded that ultrasound guidance improves success rates and reduces complications associated with arterial line placement, especially in patients who are difficult to cannulate by traditional methods.<br>In summary, ultrasound-guided arterial line placement offers clear benefits in terms of success rates and safety, particularly in difficult cases, though its utility can be influenced by the operator's experience and the availability of equipment | | |
| What is the evidence for using rocuronium for neuromuscular blockade following the use of sugammadex? Please include at least one peer-reviewed reference. (E3) | Rocuronium is frequently used for neuromuscular blockade (NMB) in various surgical procedures, especially when rapid onset and reliable reversal are desired. Sugammadex, a selective relaxant binding agent, can rapidly and effectively reverse the effects of rocuronium by encapsulating the rocuronium molecules. After reversal with sugammadex, one concern is whether re-administration of rocuronium (if needed for further procedures) is still effective and safe.<br>**Evidence Supporting Rocuronium After Sugammadex Use:** | 2 | 3 |

| | | | |
|---|---|---|---|
| | 1. **Rapid Re-administration**: Studies have shown that following sugammadex administration, there is a period during which re-administration of rocuronium is less effective. This is because the free rocuronium molecules are bound and removed from circulation by sugammadex, reducing the effectiveness of subsequent doses. However, after a sufficient interval (generally 5-10 minutes or more), a higher dose of rocuronium can be administered successfully to re-establish neuromuscular blockade.<br>2. **Higher Doses Needed**: A key finding from the research is that a larger dose of rocuronium may be required to achieve the same degree of blockade compared to the initial dose prior to sugammadex reversal. Studies suggest that doubling the dose of rocuronium (1.2 mg/kg compared to the standard 0.6 mg/kg) can overcome this issue.<br>3. **Alternative Neuromuscular Blockers**: If rapid re-induction of neuromuscular blockade is necessary shortly after sugammadex, some clinicians prefer using **cisatracurium**, which is not affected by sugammadex, instead of rocuronium. This avoids the need for higher doses of rocuronium.<br>**Clinical Study Example:**<br>In a randomized controlled trial by Sorgenfrei et al. (2012), the effects of repeated administration of rocuronium after sugammadex were evaluated. The study found that when sugammadex was administered to reverse a moderate blockade (with a dose of 4 mg/kg), re-administration of rocuronium could effectively induce a neuromuscular blockade, but a larger dose was necessary. The study concluded that if a high dose of rocuronium (1.2 mg/kg) is administered after sugammadex, effective neuromuscular blockade can be re-established.<br>**Reference:**<br>• Sorgenfrei, M., Norrild, K., Larsen, P. B., Stensballe, J., Østergaard, D., & Prins, M. E. (2012). Repeated dose–response relationships of rocuronium and sugammadex in the pig. *Anesthesia & Analgesia*, 115(6), 1353-1359. doi:10.1213/ANE.0b013e318269fdd4 | | |
| What study calculated the minimal alveolar concentration of sevoflurane in | The minimal alveolar concentration (MAC) of sevoflurane in humans was first calculated in a study published by Yasuo Eger and his colleagues. Here is the full citation: | 2 | 4 |

| | | | |
|---|---|---|---|
| human? Please provide the full citation. (E7) | Eger, E. I., Saidman, L. J., & Brandstater, B. (1965). **Minimum alveolar anesthetic concentration: A standard of anesthetic potency**. *Anesthesiology*, 26(6), 756-763.<br>Note that this original study determined the concept of MAC using various anesthetics. For sevoflurane specifically, a widely cited study was later conducted by:<br>Yasuda, N., Lockhart, S. H., Eger, E. I., Weiskopf, R. B., Liu, J., Laster, M. J., & Taheri, S. (1991). **Kinetics of desflurane, isoflurane, and sevoflurane in humans.** *Anesthesiology*, 74(2), 489-498.<br>Both studies are critical in the context of determining MAC for inhaled anesthetics like sevoflurane. | | |
| Can you list some published clinical trial which compared rapid sequence intubation with and without cricoid pressure? | Several clinical trials have compared the use of cricoid pressure (also known as Sellick's maneuver) in rapid sequence intubation (RSI), examining its effectiveness and safety. Here are some notable studies:<br>**1. The Cricoid Pressure Controversy (2005)**<br>• **Authors**: Salem MR, Khorasani A, Crystal GJ<br>• **Journal**: Anesthesia & Analgesia<br>• **Study**: This review discusses whether cricoid pressure should continue to be used as a standard part of RSI. It highlights various studies questioning its effectiveness in preventing aspiration and considers potential harms, like hindering intubation.<br>**2. Effect of Cricoid Pressure on the Esophageal Position (2011)**<br>• **Authors**: Smith KJ, Dobranowski J, Yip G, Dauphin A, Choi PTL<br>• **Journal**: Anesthesia & Analgesia<br>• **Study**: This randomized study used magnetic resonance imaging (MRI) to examine the position of the esophagus during cricoid pressure in awake patients. It suggested that cricoid pressure might not reliably occlude the esophagus and can potentially displace it laterally, raising doubts about its effectiveness in preventing regurgitation.<br>**3. Cricoid Pressure During Rapid Sequence Induction: Analyzing its Effect on First-Attempt Intubation Success (2014)**<br>• **Authors**: Law JA, Duggan LV, Asselin M, Murphy MF, Kovacs G<br>• **Journal**: Canadian Journal of Anesthesia | 2 | 3 |

- **Study**: This study evaluated whether the application of cricoid pressure during RSI impacted the success rate of intubation on the first attempt. It concluded that cricoid pressure had minimal impact on first-pass success but could contribute to difficulty in intubation.

**4. Cricoid Pressure Versus Sham in RSI: A Randomized Controlled Trial (IRIS, 2019)**

- **Authors**: Algie CM, Mahar RK, Tan HB, Wilson G, Mahar PD, Wasiak J
- **Journal**: British Journal of Anaesthesia
- **Study**: This multicenter randomized controlled trial compared the use of cricoid pressure versus a sham maneuver in RSI. The study found no significant difference in the incidence of pulmonary aspiration between the two groups, calling into question the need for routine cricoid pressure.

**5. Cricoid Pressure in RSI: A Randomized, Double-Blind, Controlled Study (2020)**

- **Authors**: Bhatia N, Bhalla T, Martin DP, Tobias JD
- **Journal**: Journal of Clinical Anesthesia
- **Study**: This study focused on pediatric patients undergoing general anesthesia with RSI. It evaluated the effectiveness of cricoid pressure in reducing the risk of aspiration and found no significant difference in aspiration rates between groups, with or without cricoid pressure.

**6. The Impact of Cricoid Pressure on Airway Management During Rapid Sequence Intubation (2021)**

- **Authors**: Chrimes N, Higgs A
- **Journal**: Anaesthesia
- **Study**: This recent study looked at the potential hindrance of intubation due to cricoid pressure, especially in difficult airways. It highlighted that cricoid pressure could impair laryngoscopic view, complicating airway management in some cases.

These studies reflect the ongoing debate about the efficacy of cricoid pressure in preventing aspiration during RSI, with some evidence suggesting it may interfere with airway management and no clear benefit in reducing aspiration risk

| | | | |
|---|---|---|---|
| How can I approach placing spinal anesthesia at the T11 level? (B1) | Spinal anesthesia is generally placed in the lower lumbar region (below L2), as the spinal cord ends at approximately L1-L2 in adults. The reason for this is to avoid the risk of injuring the spinal cord. However, in certain circumstances, you may need to perform a spinal block at a higher level for specific surgeries or conditions.<br>Placing spinal anesthesia at the T11 level requires extreme caution, and it's important to understand the risks involved. Here's how you could approach it:<br>**1. Review Indications and Risks**<br>• **Indication**: Higher spinal anesthesia may be indicated for certain surgeries involving the lower thoracic or upper abdominal region, though it is more common to use epidural anesthesia in these cases due to greater safety margins.<br>• **Risks**: Approaching the T11 level increases the risk of damaging the spinal cord, leading to serious complications such as paralysis. Hence, meticulous attention to anatomy is required.<br>**2. Patient Positioning**<br>• **Position**: Typically, the patient is positioned in either a lateral decubitus or sitting position to expose the spinous processes.<br>• **Landmarks**: Locate the T11 vertebra by palpating the lower ribs and moving upward. The T12 vertebra aligns with the lowest rib (12th rib), and T11 is just above that.<br>**3. Choose the Right Needle**<br>• **Needle**: You will typically use a 22–25 gauge Quincke or Whitacre needle, as these are fine enough to minimize trauma.<br>• **Needle Insertion**: Ensure a midline approach to avoid lateral injury to nerve roots.<br>**4. Techniques for Needle Placement**<br>• **Technique**: Advance the needle slowly while feeling for the typical "give" as it passes through the ligamentum flavum and enters the subarachnoid space.<br>• **CSF Aspiration**: Once you're in the subarachnoid space, you will see cerebrospinal fluid (CSF) flow back into the hub of the needle.<br>**5. Administering the Anesthetic**<br>• **Dose Adjustment**: Because you are injecting at a higher level, you might need to adjust the dose of local anesthetic to prevent a high | 2 | 3 |

| | | | |
|---|---|---|---|
| | spinal block, which can lead to dangerous complications like bradycardia or respiratory depression.<br>• **Monitoring**: Closely monitor the patient after injection to ensure the block remains at a safe level.<br>**6. Consider Alternatives**<br>• **Epidural Anesthesia**: For procedures requiring anesthesia at the T11 level or higher, an epidural might be safer because the catheter can be placed at a lower level and the medication can be titrated upward.<br>**Summary**<br>Spinal anesthesia at the T11 level is not commonly done due to the increased risk of spinal cord injury. If higher anesthesia is needed, it's generally safer to perform an epidural. However, if absolutely necessary, meticulous attention to anatomy, positioning, and dosage is crucial.<br>Would you like more detailed information on the spinal anesthesia technique? | | |
| What is the dose of sugammadex for the reversal of succinylcholine? | Sugammadex is not used to reverse **succinylcholine**, as succinylcholine is a depolarizing neuromuscular blocking agent and it is metabolized rapidly by plasma cholinesterase. The primary treatment for prolonged succinylcholine action or succinylcholine-induced apnea is supportive care, including ventilation, rather than pharmacologic reversal.<br>Sugammadex is specifically designed to reverse **aminosteroid** neuromuscular blocking agents like rocuronium and vecuronium. It encapsulates and inactivates these drugs but has no effect on depolarizing agents like succinylcholine.<br>If you're dealing with prolonged effects of succinylcholine, the management typically focuses on maintaining airway and breathing support until the drug wears off. | 2 | 6 |
| What's the advantage of inhalational induction in patients with a history of malignant hyperthermia? | Inhalational induction can be advantageous for patients with a history of malignant hyperthermia (MH) for several reasons:<br>1. **Avoidance of Triggering Agents**: Inhalational induction typically involves the use of volatile anesthetics or nitrous oxide, which can be preferable in patients susceptible to MH. This is particularly important because certain agents, such as succinylcholine and some volatile anesthetics (e.g., halothane), are known to trigger MH. | 3 | 2 |

| | | | |
|---|---|---|---|
| | 2. **Controlled Environment**: Inhalational induction allows for gradual titration of anesthetic agents, enabling anesthesiologists to monitor the patient's response and adjust the dosage accordingly. This careful titration can help prevent sudden changes that might provoke an MH episode.<br>3. **Ease of Transition**: Inhalational induction allows for a smoother transition to maintenance anesthesia with volatile agents, which can also help avoid the use of triggering agents that might cause an MH reaction.<br>4. **Less Stressful for Patients**: Some patients find inhalational induction less stressful compared to intravenous induction, particularly if they have anxiety about needles. This can help facilitate a more calm and controlled induction process.<br>5. **Ability to Maintain Oxygenation**: Inhalational agents can provide a level of respiratory support, allowing for better oxygenation during induction, which is beneficial in patients who may have underlying respiratory issues.<br>6. **Rapid Recovery**: Volatile anesthetics allow for rapid recovery once the procedure is completed, as they can be quickly eliminated from the body, which is beneficial for patients who may require close monitoring after surgery.<br>While inhalational induction has these advantages, it's crucial to have a thorough preoperative evaluation and ensure that the entire anesthesia team is aware of the patient's MH history. This awareness ensures that all precautions are taken to prevent MH during the surgical procedure. | | |
